# Precision medicine for mood disorders: objective assessment, risk prediction, pharmacogenomics, and repurposed drugs

**DOI:** 10.1038/s41380-021-01061-w

**Published:** 2021-04-08

**Authors:** H. Le-Niculescu, K. Roseberry, S. S. Gill, D. F. Levey, P. L. Phalen, J. Mullen, A. Williams, S. Bhairo, T. Voegtline, H. Davis, A. Shekhar, S. M. Kurian, A. B. Niculescu

**Affiliations:** 1 Department of Psychiatry, Indiana University School of Medicine, Indianapolis, IN USA; 2 Stark Neuroscience Research Institute, Indiana University School of Medicine, Indianapolis, IN USA; 3 Indianapolis VA Medical Center, Indianapolis, IN USA; 4 Scripps Health and Department of Molecular Medicine, Scripps Research, La Jolla, CA USA; 5 Present Address: Department of Psychiatry, Yale University School of Medicine, New Haven, CT USA; 6 Present Address: VA Maryland Health Care System/University of Maryland School of Medicine, Baltimore, MD USA; 7 Present Address: Office of the Dean, University of Pittsburgh School of Medicine, Pittsburgh, PA USA

**Keywords:** Biomarkers, Genetics

## Abstract

Mood disorders (depression, bipolar disorders) are prevalent and disabling. They are also highly co-morbid with other psychiatric disorders. Currently there are no objective measures, such as blood tests, used in clinical practice, and available treatments do not work in everybody. The development of blood tests, as well as matching of patients with existing and new treatments, in a precise, personalized and preventive fashion, would make a significant difference at an individual and societal level. Early pilot studies by us to discover blood biomarkers for mood state were promising [1], and validated by others [2]. Recent work by us has identified blood gene expression biomarkers that track suicidality, a tragic behavioral outcome of mood disorders, using powerful longitudinal within-subject designs, validated them in suicide completers, and tested them in independent cohorts for ability to assess state (suicidal ideation), and ability to predict trait (future hospitalizations for suicidality) [3–6]. These studies showed good reproducibility with subsequent independent genetic studies [7]. More recently, we have conducted such studies also for pain [8], for stress disorders [9], and for memory/Alzheimer’s Disease [10]. We endeavored to use a similar comprehensive approach to identify more definitive biomarkers for mood disorders, that are transdiagnostic, by studying mood in psychiatric disorders patients. First, we used a longitudinal within-subject design and whole-genome gene expression approach to discover biomarkers which track mood state in subjects who had diametric changes in mood state from low to high, from visit to visit, as measured by a simple visual analog scale that we had previously developed (SMS-7). Second, we prioritized these biomarkers using a convergent functional genomics (CFG) approach encompassing in a comprehensive fashion prior published evidence in the field. Third, we validated the biomarkers in an independent cohort of subjects with clinically severe depression (as measured by Hamilton Depression Scale, (HAMD)) and with clinically severe mania (as measured by the Young Mania Rating Scale (YMRS)). Adding the scores from the first three steps into an overall convergent functional evidence (CFE) score, we ended up with 26 top candidate blood gene expression biomarkers that had a CFE score as good as or better than SLC6A4, an empirical finding which we used as a de facto positive control and cutoff. Notably, there was among them an enrichment in genes involved in circadian mechanisms. We further analyzed the biological pathways and networks for the top candidate biomarkers, showing that circadian, neurotrophic, and cell differentiation functions are involved, along with serotonergic and glutamatergic signaling, supporting a view of mood as reflecting energy, activity and growth. Fourth, we tested in independent cohorts of psychiatric patients the ability of each of these 26 top candidate biomarkers to assess state (mood (SMS-7), depression (HAMD), mania (YMRS)), and to predict clinical course (future hospitalizations for depression, future hospitalizations for mania). We conducted our analyses across all patients, as well as personalized by gender and diagnosis, showing increased accuracy with the personalized approach, particularly in women. Again, using SLC6A4 as the cutoff, twelve top biomarkers had the strongest overall evidence for tracking and predicting depression after all four steps: NRG1, DOCK10, GLS, PRPS1, TMEM161B, GLO1, FANCF, HNRNPDL, CD47, OLFM1, SMAD7, and SLC6A4. Of them, six had the strongest overall evidence for tracking and predicting both depression and mania, hence bipolar mood disorders. There were also two biomarkers (RLP3 and SLC6A4) with the strongest overall evidence for mania. These panels of biomarkers have practical implications for distinguishing between depression and bipolar disorder. Next, we evaluated the evidence for our top biomarkers being targets of existing psychiatric drugs, which permits matching patients to medications in a targeted fashion, and the measuring of response to treatment. We also used the biomarker signatures to bioinformatically identify new/repurposed candidate drugs. Top drugs of interest as potential new antidepressants were pindolol, ciprofibrate, pioglitazone and adiphenine, as well as the natural compounds asiaticoside and chlorogenic acid. The last 3 had also been identified by our previous suicidality studies. Finally, we provide an example of how a report to doctors would look for a patient with depression, based on the panel of top biomarkers (12 for depression and bipolar, one for mania), with an objective depression score, risk for future depression, and risk for bipolar switching, as well as personalized lists of targeted prioritized existing psychiatric medications and new potential medications. Overall, our studies provide objective assessments, targeted therapeutics, and monitoring of response to treatment, that enable precision medicine for mood disorders.

## Introduction

“How weary, stale, flat, and unprofitable

Seem to me all the uses of this world!”

– W. Shakeaspeare, *Hamlet*

“There are good and bad times, but our mood changes more often than our fortune.”

– Thomas Carlyle

Mood disorders affect up to 1 in 4 individuals in their lifetime. Depression in particular is the leading cause of disability for ages 15–44, a prime productive and reproductive age. Due to the lack of objective tests and the perceived presence of stigma, mood disorders are often underdiagnosed or misdiagnosed (depression instead of bipolar disorder). They are also sub-optimally treated, can lead to self-medication with alcohol and drugs, and may culminate in some cases with suicide.

Blood biomarkers are emerging as important tools in disorders where subjective self-report of an individual or clinical impression of a healthcare professional are not always reliable, and for predicting future risk before the disorder (re-)occurs. They also open the door to precise, personalized matching with medications, and objective monitoring of response to treatment. Pioneering early work by our group has identified candidate blood gene expression biomarkers for mood state using a case–case design and a visual analog scale (VAS) (Le-Niculescu et al.) [1]. Those biomarkers were also validated independently as tracking response to cognitive-behavioral therapy by another group [2]. Recent work by our group has identified blood gene expression biomarkers that track suicidality, a tragic outcome of mood disorders, using a new powerful within- subject longitudinal stepwise approach [4, 5, 11]. These studies show good reproducibility and provide a Rosetta Stone for recent multiple genetic studies of suicide (GWAS, family based) [7]. More recently, we have conducted such studies for pain [8], for stress disorders [9], and for memory/Alzheimer’s Disease [10].

**Fig. 1:**
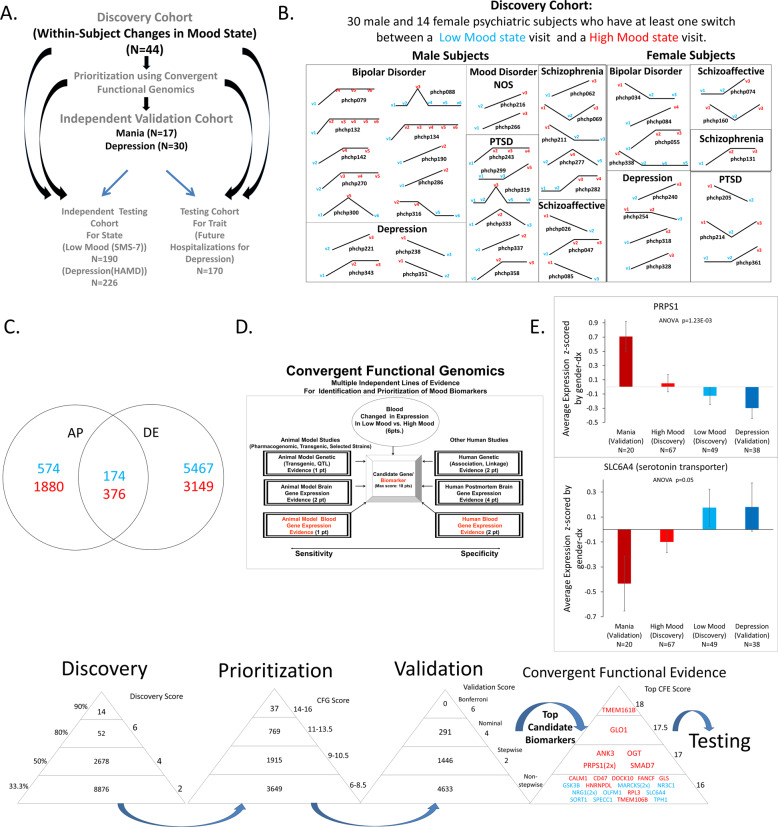
Steps 1–3: Discovery, Prioritization and Validation of Biomarkers for Mood. **A** Cohorts used in study, depicting flow of discovery, prioritization, and validation of biomarkers from each step. **B** Discovery cohort longitudinal within-subject analysis. Phchp### is study ID for each subject. V# denotes visit number. **C** Differential gene expression in the Discovery cohort- number of genes identified with differential expression (DE) and absent–present (AP) methods with an internal score of 2 and above. Red increased in expression in high mood, blue decreased in expression in high mood. At the discovery step probesets are identified based on their score for tracking mood with a maximum of internal points of 6 (33% (2pt), 50% (4pt) and 80% (6pt)). **D** Prioritization with CFG for prior evidence of involvement in mood disorders. In the prioritization step probesets are converted to their associated genes using Affymetrix annotation and GeneCards. Genes are prioritized and scored using CFG for mood evidence with a maximum of 12 points. Genes scoring at least 6 points out of a maximum possible of 18 total discovery  and prioritization points are carried to the validation step. **E** Validation in two independent cohort of psychiatric patients with clinically severe depression (HAMD ≥ 22) and clinically severe mania (YMRS ≥ 20). In the validation step biomarkers are assessed for stepwise change from the validation group with mania, to the discovery groups of subjects with high mood, low mood, to the validation group with depression, using ANOVA. *N* number of testing visits. Two hundred ninety-one biomarkers were nominally significant, and 1446 biomarkers were stepwise changed. PRPS1 and SLC6A4  are examples of significantly increased, respectively, decreased, biomarkers in validation. There were 26 markers that had an overall Convergent Functional Evidence (CFE) score from Steps 1–3 that was at least as good as SLC6A4, which serves as a de facto positive control and that we decided to use as a cutoff. The markers in red are increased in high mood, the markers in blue are decreased in high mood/increased in depression (color figure online).

**Fig. 2:**
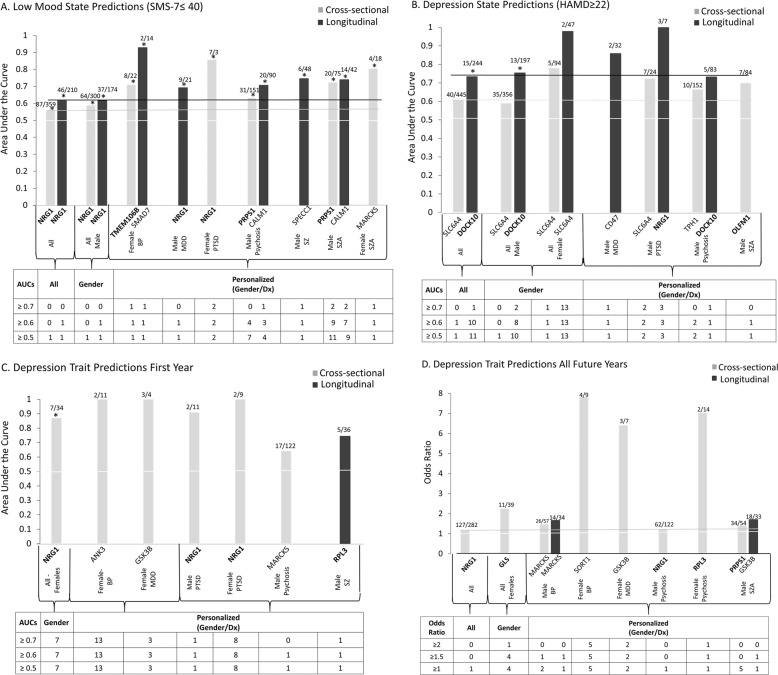
Best single biomarkers predictors for depression, state and trait. From top candidate biomarkers after Steps 1–3 (discovery, prioritization, validation-bold) (*n* = 26). Bar graph shows best predictive biomarkers in each group. All markers are nominally significant *p* < 0.05. Table underneath the figures displays the actual number of biomarkers for each group whose ROC AUC *p* values (**A**–**C**,) and Cox odds ratio (OR) *p* values (**D**) are at least nominally significant. Some gender and diagnosis group are missing from the graph as they did not have any significant biomarkers, or sufficient timepoints in the case of longitudinal predictions. Cross-sectional is based on levels at one visit. Longitudinal is based on levels at multiple visits (integrates levels at most recent visit, maximum levels, slope into most recent visit, and maximum slope). Dividing lines represent the cutoffs for a test performing at chance levels (white), and at the same level as the best biomarkers for all subjects in cross-sectional (gray) and longitudinal (black) based predictions. All biomarkers perform better than chance. Biomarkers performed better when personalized by gender and diagnosis, particularly in females. * survived Bonferroni correction for the number of candidate biomarkers tested (*n* = 26).

We endeavored to use a similar comprehensive approach to identify more definitive biomarkers for mood disorders in general, and depression in particular. Psychiatric patients may have an increased vulnerability to mood disorders, regardless of their primary diagnosis, as well as increased reasons for mood disorders, due to their often-adverse life trajectory. As such, they may be a particularly suitable population in which to try to identify blood biomarkers for mood disorders, that are generalizable and transdiagnostic. First, we used a powerful longitudinal within-subject design (Fig. 1 and Table 1) in individuals with psychiatric disorders to discover blood gene expression changes between self-reported low mood and high mood states, measured by a VAS, called the Simplified Affective State Scale (SASS), previously described by us [4, 5, 11, 12], which has a subscale of seven items related to mood (SMS-7) (Fig. S1). Second, we prioritized this list of candidate biomarkers with a Bayesian-like CFG approach, comprehensively integrating previous human and animal model evidence in the field. Third, we validated our top candidate biomarkers for mood from discovery and prioritization in an independent cohort of psychiatric subjects with clinically severe depression (as measured by HAMD) or with clinically severe mania (as measured by YMRS). We also analyzed the biological pathways and networks they are involved in (Table 2). Fourth, we tested if the top candidate biomarkers from the first three steps are able to predict low mood state, clinical depression state, and future hospitalizations with depression, in another independent cohort of psychiatric subjects. We tested the biomarkers in all subjects in the test cohort, as well as in a more personalized fashion by gender and psychiatric diagnosis (Fig. 2A–D). We also conducted similar analyses for predictions of high mood, clinical mania state, and future hospitalizations with mania (Table 3B, C, and Supplementary Information—Pathways, Predictions and Reproducibility). Next, we identified which of our biomarkers are targets of existing drugs and thus can be used for pharmacogenomic population stratification and measuring of response to treatment for depression. We also used the biomarker gene expression signatures to interrogate the Connectivity Map database from Broad/MIT, and the NIH LINCS database, in order to identify drugs and natural compounds that can be repurposed for treating and preventing depression, including bipolar depression. Finally, we provide an example of how a personalized patient report can be generated for clinicians to use, reflecting the objective assessment of depression state, future risk of severe depression, risk of bipolarity, matching with existing psychiatric medications, matching with non-psychiatric/repurposed medications, and monitoring response to treatment.

**Table 1:** Demographics of cohorts used.

	Number of subjects	Gender	Diagnosis	Ethnicity	Age Mean (SD)	*T*-test for age
**Discovery**						
Discovery cohort -within-subject changes in mood (SMS-7)	44 (with 134 visits)	Male = 30 Female = 14	BP = 14 MDD = 8 SZA = 5 SZ = 6 PTSD = 8 MOOD = 2	EA = 33 AA = 9 Asian = 1 Hispanic=1	All = 50.76 (6.48)	
**Validation**						
Independent validation cohort -clinically severe depression (HAMD ≥ 22)	30	Male = 23 Female = 7	BP = 12 MDD = 12 SZA = 2 PTSD = 3 PSYCH = 1	EA = 27 AA = 2 Asian = 1	All = 49.42 (7.06)	
Independent validation cohort -clinically severe mania (YMRS ≥ 20)	17	Male = 16 Female = 1	BP = 8 SZA = 6 SZ = 2 PSYCH = 1	EA = 11 AA = 6	All = 48.25 (8.21)	
**Testing**						
Independent testing cohort State predictions Low Mood (SMS-7 ≤ 40)	190	Male = 153 Female = 37	BP = 52 MDD = 30 SZA = 48 SZ = 36 PTSD = 16 MOOD = 5 PSYCH = 3	EA = 118 AA = 69 Hispanic= 2 Mixed = 1	All = 50.52 (8.58) Low mood = 49 (*n* = 87) Others = 50.88 (*n* = 359	*T*-test for age between low mood vs. Others 0.10469
Independent testing cohort State predictions Clinical Depression(HAMD ≥ 22)	226	Male = 181 Female = 45	BP = 74 MDD = 39 SZA = 48 SZ = 36 PTSD = 21 MOOD = 5 PSYCH = 3	EA = 156 AA = 66 Asian = 1 Hispanic= 2 Mixed = 1	All = 46.71 (9.42) Clinical depression = 44.4 (*n* = 40) Others = 46.9 (*n* = 445)	*T*-test for age between clinical depression vs. others 0.177087984
Independent testing cohort Trait predictions Hospitalizations with Depression First year following initial visit	147	Male = 130 Female = 17	BP = 37 MDD = 27 SZA = 32 SZ = 33 PTSD = 13 MOOD = 3 PSYCH = 2	EA = 90 AA = 54 Mixed = 1 Hispanic = 2	All = 47.13 (9.38) Hosp with Depression = 46.58 (*n* = 50) Others = 47.23 (*n* = 282)	*T*-test for age between hosp with depression vs. others 0.701909278
Independent testing cohort Trait predictions Hospitalizations with Depression All future years following initial visit	170	Male = 150 Female = 20	BP = 41 MDD = 29 SZA = 40 SZ = 39 PTSD = 14 MOOD = 5 PSYCH = 2	EA = 102 AA = 65 Mixed = 1 Hispanic = 2	All = 49.4 (9.78) Hosp with depression = 49.3 (*n* = 127) Others = 49.4 (*n* = 282)	*T*-test for age between hosp with depression vs. others 0.93467396
Independent testing cohort State predictions High Mood (SMS-7 ≥ 60)	190	Male = 153 Female = 37	BP = 52 MDD = 30 SZA = 48 SZ = 36 PTSD = 16 MOOD = 5 PSYCH = 3	EA = 118 AA = 69 Hispanic = 2 Mixed = 1	All = 50.52 (8.58) High mood = 50.6 (*n* = 185) Others = 50.5 (*n* = 261)	*T*-test for age between high mood vs. others 0.877948
Independent testing cohort State predictions Clinical Mania (YMRS ≥ 20)	97	Male = 73 Female = 24	BP = 37 MDD = 13 SZA = 18 SZ = 18 PTSD = 10 MOOD = 1	EA = 72 AA = 22 Hispanic = 2 Mixed = 1	All = 39.4 (8.83) Clinical Mania=38.9 (*n* = 13) = 38.9 (*n* = 13) Others=39.4(*n* = 197)	*T*-test for age between mania vs. others 0.883113775
Independent testing cohort Trait predictions Hospitalizations with Mania First year following initial visit	147	Male = 130 Female = 17	BP = 37 MDD = 27 SZA = 32 SZ = 33 PTSD = 13 MOOD = 3 PSYCH = 2	EA = 90 AA = 54 Mixed = 1 Hispanic = 2	All = 47.13 (9.38) Hosp with Mania = 45.5 (*n* = 11) Other = =47.2 (*n* = 321)s = 47.2 (*n* = 321)	*T*-test for age between hosp with mania vs. others 0.588179
Independent testing cohort Trait predictions Hospitalizations with Mania All future years following initial visit	117	Male = 102 Female = 15	BP = 34 MDD = 17 SZA = 26 SZ = 26 PTSD = 11 MOOD = 2 PSYCH = 1	EA = 74 AA = 40 Mixed = 1 Hispanic = 2	All=44.39 (9.01) Hosp with Mania = 43.7(*n* = 37) Others = 44.5 (*n* = 220)	*T*-test for age between hosp with mania vs. others 0.692290398

*BP* bipolar, *MDD* major depressive disorder, *SZA* schizoaffective disorder, *SZ* schizophrenia, *PTSD* post-traumatic stress disorder, *MOOD* mood disorder nos, *PSYCH* psychosis nos.

**Table 2:** Biology of mood biomarkers. **A** Pathway analyses. **B** Diseases.

A.	DAVID GO functional annotation biological processes					KEGG pathways				Ingenuity pathways		
	#	Term	Count	%	*P* value	Term	Count	%	*P* value	Top canonical pathways	*P* value	Overlap
Top candidate biomarkers (*n* = 26 probesets, 23 genes)	1	**Regulation of cell differentiation**	9	39.1	5.20E−04	**Neurotrophin signaling pathway**	3	13	3.10E−02	**Serotonin receptor signaling**	8.62E−04	4.7% 2/43
	2	**Rhythmic process**	5	21.7	6.80E−04					**Glutamate receptor signaling**	1.51E−03	3.5% 2/57
	3	Regulation of peptidyl-threonine phosphorylation	3	13	1.10E−03					ErbB2-ErbB3 Signaling	1.96E−03	3.1% 2/65
	4	Mesenchymal cell development	4	17.4	1.30E−03					Glutamine Degradation I	2.02E−03	50.0% 1/2
	5	**Circadian rhythm**	4	17.4	1.40E−03					Cell Cycle: G1/S Checkpoint Regulation	2.08E−03	3.0% 2/67

B.	David					Ingenuity pathways disease		
	#	Term	Count	%	*P* value	Diseases and disorders	*P* value	# Molecules
Top candidate biomarkers (*n* = 26 probesets, 23 genes)	1	**Weight gain**	5	21.7	2.90E−05	Neurological disease	2.85E−03 to 5.36E−09	18
	2	**Major depressive disorder**	4	17.4	4.00E−05	Psychological disorders	1.41E−03 to 1.14E−08	14
	3	Schizophrenia	8	34.8	5.10E−05	Organismal injury and abnormalities	3.03E−03 to 1.91E−07	23
	4	Depression	5	21.7	5.40E−05	Skeletal and muscular disorders	2.70E−03 to 1.44E−06	11
	5	Psychosis	3	13	1.60E−04	Metabolic disease	2.02E−03 to 1.51E−06	11

Bold highlights top results of interest.

**Table 3:** Convergent functional evidence (CFE): **A** Top biomarkers for low mood/ depression. *n* = 12 genes, 13 probesets, using as a cutoff the score for SLC6A4; **B** Top biomarkers for bipolar mood disorders. *n* = 6 genes, using as a cutoff the score for SLC6A4. These genes are contained in the list of top biomarkers for depression in **A**. **C** Top Biomarkers for High Mood/ Mania. *n* = 2 genes, using as a cutoff the score for SLC6A4. RPL3 is not overlapping with the list of top biomarkers for depression in **A**.

A. DEPRESSION											
Gene symbol/gene name	Probesets	Step 1 Discovery (direction of change in high mood) method/score/% 6 pts	Step 2 External convergent functional genomics (CFG) evidence for involvement in mood disorders score 12 pts	Step 3 Validation ANOVA *p* value/ score 6 pts	Step 4 Significant predictions of low mood state ROC AUC/*p* value 3 pts all 2pts gender 1pts gender /dx	Step 4 Significant predictions of depression state ROC AUC/*p* value 3 pts all 2pts gender 1pts gender /dx	Step 4 Significant predictions of first year hosp for depression ROC AUC/ *p* value 3 pts All 2pts gender 1pts gender /dx	Step 4 Significant predictions of all future hosp for depression OR/OR *p* value 3 pts all 2pts gender 1pts gender /dx	Other psychiatric and related disorders evidence	Drugs that modulate the biomarker in same direction as high mood	CFE polyevidence score for involvement in depression (based on Steps 1–4)
**NRG1** Neuregulin 1	208230_s_at	(D) DE/2 33.7%	10	2.80E−03/4 Nominal	**All** **C:**(87/446) 0.56/4.03E−02 **L:**(46/256) 0.62/6.78E-03 **Gender** **Males** **C:**(64/364) 0.59/1.30E−02 **L:**(37/211) 0.62/1.29E-02 **Gender/Dx** M-MDD **L:**(9/30) 0.69/4.93E−02	**Gender** **Females** **L:**2/49 0.87/3.85E−02 **Gender /Dx** **M-PTSD** **L:**3/10 1/8.35E−03	**Gender** **Females** **C:**(7/41) 0.87/1.15E-03 **Gender /Dx** F-MDD **C:**(3/7) 1/1.69E−02 **F-PTSD** **C:**(2/11) 1/1.69E-02 **M-PTSD** **C:**(2/13) 0.91/3.78E−02	**All** **C:**(127/409) 1.17/2.51E-02 **Gender** **Females** **C:**(11/50) 1.59/4.99E−02 **Gender /Dx** **M-PSYCHOSIS** **C:**(62/184) 1.22/2.36E-02 **M-SZA** **C:**(34/88) 1.34/2.99E−03	Aging Anxiety Dementia Memory Pain Psychosis Stimulants Stress Suicide SZ	Antidepressants Antipsychotic Antipsychotics Escitalopram Lithium	26
**DOCK10** dedicator of cytokinesis 10	219279_at	(I) DE/2 41.5%	10	4.95E-02/4 Nominal	**Gender /Dx** **M-** **PSYCHOSIS** **C:**31/182 0.63/1.24E−02 **M-SZA** **C:**20/95 0.7/2.92E-03 **L:**14/56 0.65/4.79E−02	**All** **L:**15/259 0.73/1.17E-03 **Gender** **Males** **L:**13/210 0.75/1.05E−03 **Gender /Dx** **M-PSYCHOSIS** **L:**5/88 0.73/4.10E-02 **M-PTSD** **L:**3/10 0.95/1.52E−02	**Gender** **Females** **C:**7/41 0.71/4.48E-02 **Gender /Dx** **F-BP** **C:**2/13 0.91/3.78E−02 **F-PTSD** **C:**2/11 0.94/2.97E-02	**Gender** **Females** **C:**11/50 1.9/3.93E−02	Aging Alcohol BP Dementia Suicide Social defeat Stress SZ	Ketamine Physical and Cognitive stimulation	24
**GLS** glutaminase	203159_at	(I) DE/4 53.7%	8	1.90E-02/4 Nominal	**Gender /Dx** **F-PTSD** **C:**7/10 0.86/4.37E−02 **Gender /Dx** **M-PSYCHOSIS** **L:**20/110 0.63/3.43E−02 **Gender /Dx** **M-SZA** **C:**20/95 0.63/3.26E−02 **Gender /Dx** **M-SZA** **L:**14/56 0.72/7.72E−03	**All** **L:**15/259 0.64/3.04E−02	**Gender** **Females** **C:**7/41 0.82/4.23E-03 **Gender /Dx** **F-BP** **C:**2/13 0.95/2.42E−02	**Gender** **Females** **C:**11/50 2.25/9.70E-03 **Gender /Dx** **F-BP** **C:**4/13 6.25/2.93E−02	Aging Alcohol Anxiety ASD Dementia Pain Stress Suicide SZ	Clozapine Omega-3 fatty acids Risperidone	24
**PRPS1** phosphoribosyl pyrophosphate synthetase 1	209440_at	(I) DE/4 57.3%	9	1.23E-03/4 Nominal	**Gender/Dx** **M-PSYCHOSIS** **C:**31/182 0.63/1.05E−02 **Gender /Dx** **M-SZA** **C:**20/95 0.72/1.11E-03	**All** **L:**15/259 0.63/4.48E−02 **Gender** **Males** **L:**13/210 0.64/4.93E-02	**Gender /Dx** **F-PTSD** **C:**2/11 0.94/2.97E−02	**Gender** **Females** **C:**11/50 1.85/3.28E-02 **Gender /Dx** **M-SZA** **C:**34/88 1.41/1.94E−02	Aging ASD Dementia Suicide Stress SZ	Lithium	24
**TMEM161B** transmembrane protein 161B	227861_at	(I) AP/4 62.1%	10	7.11E-03/4 Nominal	**Gender /Dx** **M-SZA** **C:**20/95 0.64/2.65E−02	**All** **L:**15/259 0.63/4.48E-02 **Gender** **Males** **L:**13/210 0.66/3.02E−02 **Gender /Dx** **M-PTSD** **L:**3/10 0.86/4.37E-02	**Gender** **Females** **C:**7/41 0.79/8.41E−03 **Gender /Dx** **F-BP** **C:**2/13 0.91/3.78E-02 **Gender /Dx** **F-PTSD** **C:**2/11 0.89/4.95E−02		Alcohol ASD Suicide Neurological Sleep Stress		24
**GLO1** glyoxalase I	200681_at	(I) DE/2 41.5%	12	2.11E-02/4 Nominal	**Gender /Dx** **M-SZA** **C:**20/95 0.66/1.33E−02 **Gender /Dx** **M-SZA** **L:**14/56 0.66/4.09E-02	**Gender** **Males** **L:**13/210 0.64/4.69E−02 **Gender /Dx** **M-PTSD** **C:**7/24 0.72/4.62E-02	**Gender /Dx** **F-BP** **C:**2/13 0.91/3.78E−02	**Gender /Dx** **F-BP** **C:**4/13 3.32/4.97E-02	Anxiety ASD Dementia Panic Sleep Stress SZ	Omega-3 fatty acids	22.5
**FANCF** Fanconi anemia complementation group F	218689_at	(I) DE/4 54.9%	8	3.46E−02/4 Nominal	**Gender /Dx** **M-SZA** **C:**20/95 0.64/3.13E-02	**All** **L:**15/259 0.67/1.37E−02 **Gender** **Males** **L:**13/210 0.66/2.57E-02	**Gender** **Females** **C:**7/41 0.72/3.58E−02 **Gender /Dx** **F-BP** **C:**2/13 1/1.50E-02 **Gender /Dx** **F-PTSD** **C:**2/11 0.89/4.95E−02		Stress		22
**HNRNPDL** heterogeneous nuclear ribonucleoprotein D like	212454_x_at	(I) DE/2 35.4%	10	3.57E-02/4 Nominal	**Gender /Dx** **M-PSYCHOSIS** **C:**31/182 0.6/4.62E−02 **Gender /Dx** **M-PSYCHOSIS** **L:**20/110 0.64/2.78E-02 **Gender /Dx** **M-SZA** **C:**20/95 0.66/1.67E−02 **Gender /Dx** **M-SZA** **L:**14/56 0.67/2.80E-02	**All** **L:**15/259 0.63/4.97E−02 **Gender** **Males** **L:**13/210 0.65/3.39E-02	**Gender /Dx** **F-BP** **C:**2/13 0.95/2.42E−02	**Gender /Dx** **F-BP** **C:**4/13 3.83/4.89E-02 **Gender /Dx** **M-SZA** **C:**34/88 1.39/3.19E−02	Aging Anxiety ASD Stress Dementia Hallucinogens Suicide Mood Stabilizers SZ	Benzodiazepines Omega-3 fatty acids	22
**CD47** CD47 molecule	213856_at	(I) AP/4 66.7%	8	1.88E-02/4 Nominal		**All** **L:**15/259 0.68/9.55E−03 **Gender** **Males** **L:**13/210 0.73/3.30E-03 **Gender /Dx** M-MDD **L:**2/34 0.86/4.61E−02	**Gender** **Females** **C:**7/41 0.71/4.48E-02 **Gender /Dx** **F-BP** **C:**2/13 0.91/3.78E−02		Aging Stress Dementia Suicide Pain Sleep SZ	Clozapine Lithium Omega-3 fatty acids Venlafaxine	21
**OLFM1** olfactomedin 1	210924_at	(D) DE/2 33.7%	10	4.75E-02/4 Nominal	**Gender /Dx** **M-PSYCHOSIS** **C:**31/182 0.59/4.85E−02 **Gender /Dx** **M-SZA** **L:**14/56 0.68/2.45E-02	**All** **L:**15/259 0.66/1.69E−02 **Gender** **Females** **L:**2/49 0.85/4.77E-02 **Gender /Dx** **M-PSYCHOSIS** **C:**10/162 0.66/4.41E−02 **Gender /Dx** **M-SZA** **C:**7/84 0.7/4.09E-02	**Gender /Dx** **F-PTSD** **C:**2/11 0.89/4.95E−02		Aging Alcohol Hallucinogens Stress Suicide SZ	Valproate	21
**SMAD7** SMAD family member 7	204790_at	(I) DE/2 42.7% (I) AP/4 54.0%	9	4.57E-02/4 Nominal	**Gender /Dx** **F-BP** **L:**2/16 0.93/2.84E−02 **Gender /Dx** **M-PSYCHOSIS** **C:**31/182 0.61/2.90E-02 **Gender /Dx** **M-SZA** **C:**20/95 0.64/3.13E−02	**All** **L:**15/259 0.65/2.26E-02 **Gender** **Males** **L:**13/210 0.66/2.83E−02			Aging Anxiety Dementia Female Suicide Stress SZ	Antidepressants	21
**SLC6A4** solute carrier family 6 (neurotransmitter transporter), member 4	242009_at	(D) DE/4 64.1%	10	5.28E-02/2 Stepwise	**Gender /Dx** **M-SZA** **L:**14/56 0.68/2.05E−02	**All** **C:**40/485 0.61/1.07E-02 **L:**15/259 0.66/1.78E−02 **Gender** **Females** **C:**5/94 0.78/1.80E-02 **Females** **L:**2/49 0.98/1.15E−02 **Males** **C:**35/391 0.59/3.93E-02 **Gender /Dx** **M-PTSD** **C:**7/24 0.72/4.61E−02			Aging Alcohol Antipsychotics Anxiety ASD Hallucinogens OCD Pain Panic Personality Stress Suicide SZ	Antidepressants Exposure therapy Lithium Omega-3 fatty acids Remifentanil	20
**Reproducibility of previous findings by us**											
Top finding from Niculescu et al. [30]											
**GRK3** G Protein-Coupled Receptor Kinase 3	204183_s_at	(D) DE/2 40.2%	7	2.27E-02/4 Nominal		**Gender** **Females** **C:** (5/94) 0.78/1.95E−02 **Gender/Dx** **F-BP** **C:** (3/40) 0.91/9.74E-03	**Gender** **Females** **C:** (7/41) 0.74/2.22E−02 **Gender/Dx** **F-MDD** **C:** (3/7) 1/1.69E-02 **M-MDD** **L:** (6/31) 0.73/4.01E−02	**Gender/Dx** **F-BP** **C:** (4/13) 3.96/2.02E-02 **M-BP** **C:** (26/83) 1.36/4.35E−02 **M-MDD** **L:** (10/33) 2.02/1.26E-02	Addictions Suicide Hallucinations Pain Panic Disorder	Haloperidol	18
Top Finding from LE−Niculescu et al. [1]											
**FGFR1** Fibroblast Growth Factor Receptor 1	211535_s_at	(D) DE/4 56.5%	10	NS	**Gender/Dx** **M-PTSD** **C:** (6/19) 0.81/1.76E-02 **L:** (2/9) 1/2.02E−02	**ALL** **C:** (40/485) 0.58/4.55E-02 **L:** (15/259) 0.64/3.11E−02 **Gender** **Males** **C:** (35/391) 0.6/2.72E-02 **L:** (13/210) 0.68/1.55E−02 **Gender/Dx** **M-PTSD** **L:** (3/10) 0.95/1.52E-02 **M-MOOD** **L:** (2/6) 1/3.2E−02	**Gender/Dx** **M-SZA** **C:** (6/67) 0.89/9.10E-04		Alcohol Aging Alzheimer’s Disease Memory ASD SZ Cocaine Stress Suicide	Lithium Clozapine Fluoxetine	19
Top Finding from Patel et al. 20010											
**ARNTL** Aryl Hydrocarbon Receptor Nuclear Translocator Like	209824_s_at	(I) DE/2 43.9%	10	1.92E−01 /2 Stepwise		**Gender/Dx** **M-MOOD** **C:** (3/11) 0.96/1.24E-02 **L:** (2/6) 1/3.2E−02	**Gender/Dx** **F-PTSD** **C:** (2/11) 0.94/2.97E-02 **F-BP** **C:** (2/13) 0.91/3.787E−02		Alcohol Suicide Stress	Lithium Ketamine Quetiapine Fluoxetine Risperidone insulin-like growth factor-1	16

B. BIPOLAR														
Gene symbol/Gene name	ProbeSet ID	Step 1 Discovery (direction of change in high mood) method/score/% 6 pts.	Step 2 External convergent functional genomics (CFG) evidence for involvement in moodscore 12 pts.	Step 3 Validation ANOVA *p* value/ Score 6 pts.	Step 4 Significant predictions of Low Mood State ROC AUC/*p* value 3 pts all 2pts gender 1 pts Gender/Dx	Step 4 Significant predictions of depression state ROC AUC/*p* value 3 pts all 2pts gender 1pts Gender/Dx	Step 4 Significant predictions of first year hosp for depression ROC AUC/*p* value 3 pts all 2pts gender 1pts gender /Dx	Step 4 Significant predictions of all future hosp for depression Cox OR/*p* value 3 pts all 2pts gender 1pts gender /Dx	Step 4 Significant predictions of High Mood State ROC AUC/p value 3 pts all 2pts gender 1pts gender /Dx	Step 4 Significant predictions of mania state ROC AUC/*p* value 3 pts all 2pts gender 1pts gender /Dx	Step 4 Significant predictions of first year hosp for mania ROC AUC/p value 3 pts all 2pts gender 1pts gender /Dx	Step 4 Significant predictions of all future hosp for mania Cox OR/*p* value 3 pts all 2pts gender 1pts gender /Dx	Drugs that modulate the biomarker in same direction as high mood	CFE polyevidence score
**NRG1** Neuregulin 1	208230_s_at	(D) DE/2 33.7%	10.00	2.80E-03/4 Nominal	**ALL** **C:** (87/446) 0.56/4.03E−02 **L:** (46/256) 0.62/6.78E-03 **Gender** **Males** **C:** (64/364) 0.59/1.30E−02 **L:** (37/211) 0.62/1.29E-02 **Gender/Dx** **M-MDD** **L:** (9/30) 0.69/4.93E−02	**Gender** **Females** **L:** (2/49) 0.87/3.85E-02 **Gender/Dx** **M-PTSD** **L:** (3/10) 1/8.35E−03	**Gender** **Females** **C:** (7/41) 0.87/1.15E-03 **Gender/Dx** **F-MDD** **C:** (3/7) 1/1.69E−02 **F-PTSD** **C:** (2/11) 1/1.69E-02 **M-PTSD** **C:** (2/13) 0.91/3.78E−02	**ALL** **C:** (127/409) 1.17/2.51E-02 **Gender** **Females** **C:** (11/50) 1.59/4.99E−02 **Gender/Dx** **M-PSYCHOSIS** **C:** (62/184) 1.22/2.36E-02 **M-SZA** **C:** (34/88) 1.34/2.99E−03	**All** **L:**(109/254) 0.58/1.39E-02 **Gender** Males **L:**(99/209) 0.59/1.38E−02			**Gender/Dx** M-PSYCHOSIS **L:**(7/55) 2.67/3.27E-02 M-SZ **L:**(4/31) 3.76/3.54E−02	Mood Stabilizers Antidepressants Antipsychotics	30
**DOCK10** Dedicator Of Cytokinesis 10	219279_at	(I) DE/2 41.5%	10.00	4.95E-02/4 Nominal	**Gender/Dx** **M-PSYCHOSIS** **C:** (31/182) 0.63/1.24E−02 **M-SZA** **C:** (20/95) 0.7/2.92E-03 **L:** (14/56) 0.65/4.79E−02	**ALL** **L:** (15/259) 0.73/1.17E-03 **Gender** **Males** **L:** (13/210) 0.75/1.05E−03 **Gender/Dx** **M-PSYCHOSIS** **L:** (5/88) 0.73/4.10E-02 **M-PTSD** **L:** (3/10) 0.95/1.52E−02	**Gender** **Females** **C:** (7/41) 0.71/4.48E-02 **Gender/Dx** **F-BP** **C:** (2/13) 0.91/3.78E−02 **F-PTSD** **C:** (2/11) 0.94/2.97E-02	**Gender** **Females** **C:** (11/50) 1.9/3.93E−02	**Gender** Females **L:** (10/45) 0.70/ 2.63E-02 **Gender/Dx** F-BP **C**: (9/30) 0.73/ 2.45E−02 F-BP **L:** (5/16) 1.0/ 9.18E-04				Physical and Cognitive stimulation	26
**GLS** Glutaminase	203159_at	(I) DE/4 53.7%	8.00	1.90E−02/4 Nominal	**Gender/Dx** **F-PTSD** **C:** (7/10) 0.86/4.37E-02 **M-PSYCHOSIS** **L:** (20/110) 0.63/3.43E-02 **Gender/Dx** **M-SZA** **C:** (20/95) 0.63/3.26E−02 **L:** (14/56) 0.72/7.72E-03	**ALL** **L:** (15/259) 0.64/3.04E−02	**Gender** **Females** **C:** (7/41) 0.82/4.23E-03 **Gender/Dx** **F-BP** **C:** (2/13) 0.95/2.42E−02	**Gender** **Females** **C:** (11/50) 2.25/9.70E-03 **Gender/Dx** **F-BP** **C:** (4/13) 6.25/2.93E-02	**Gender** Females **C**:(19/82) 0.64/ 3.20E−02 **Gender/Dx** F-BP **C**: (9/30) 0.79/ 5.28E-03 **L:** (5/16) 0.85/ 1.36E−02 M-Psychosis **L:** (48/110) 0.61/ 2.27E-02 M-SZ **L:** (24/54) 0.72/ 2.98E−03				Omega-3 fatty acids Antipsychotics	26
**PRPS1** Phosphoribosyl Pyrophosphate Synthetase 1	209440_at	(I) DE/4 57.3%	9.00	1.23E−03/4 Nominal	**Gender/Dx** **M-PSYCHOSIS** **C:** (31/182) 0.63/1.05E-02 **M-SZA** **C:** (20/95) 0.72/1.11E−03	**ALL** **L:** (15/259) 0.63/4.48E-02 **Gender** **Males** **L:** (13/210) 0.64/4.93E−02	**Gender/Dx** **F-PTSD** **C:** (2/11) 0.94/2.97E-02	**Gender** **Females** **C:** (11/50) 1.85/3.28E−02 **Gender/Dx** **M-SZA** **C:** (34/88) 1.41/1.94E-02	**Gender:** Females **C:** (19/82) 0.64/ 3.45E−02 **L:** (10/45) 0.74/1.02E−02 **Gender/Dx** F-BP **C:** (9/30) 0.75/ 1.58E-02 **L:** (5/16) 0.96/ 1.93E−03					26
**TMEM161B** Transmembrane Protein 161B	227861_at	(I) AP/4 62.1%	10.00	7.11E-03/4 Nominal	**Gender/Dx** **M-SZA** **C:** (20/95) 0.64/2.65E−02	**ALL** **L:** (15/259) 0.63/4.48E-02 **Gender** **Males** **L:** (13/210) 0.66/3.02E−02 **Gender/Dx** **M-PTSD** **L:** (3/10) 0.86/4.37E-02	**Gender** **Females** **C:** (7/41) 0.79/8.41E−03 **Gender/Dx** **F-BP** **C:** (2/13) 0.91/3.78E-02 **F-PTSD** **C:** (2/11) 0.89/4.95E−02		**Gender/Dx** F-BP **C:** (9/30) 0.69/ 4.93E-02 **L:** (5/16) 0.82/ 2.37E−02					25
**SLC6A4** Solute Carrier Family 6 Member 4	242009_at	(D) DE/4 64.1%	10	5.28E-02/2 Stepwise	**Gender/Dx** **M-SZA** **L:** (14/56) 0.68/2.05E−02	**ALL** **C:** (40/485) 0.61/1.07E-02 **L:** (15/259) 0.66/1.78E−02 **Gender** **Females** **C:** (5/94) 0.78/1.80E-02 **L:** (2/49) 0.98/1.15E−02 **Gender** **Males** **C:** (35/391) 0.59/3.93E-02 **Gender/Dx** **M-PTSD** **C:** (7/24) 0.72/4.61E−02			**Gender/Dx** F-BP **C:** (9/30) 0.73/2.45E-02 **L:** (5/16) 0.85/1.36E−02	**Gender/Dx** M-Psychosis **L:** (1/27) 1/4.76E-02	**All:** **C:** (11/332) 0.74/ 3.33E−03 **Gender:** Males **C:** (10/291) 0.72/ 8.33E-03 **Gender/Dx** M-BP **C:** (6/71) 0.77/1.35E−02 M-MDD **C:** (1/55) 1/4.45E-02		Remifentanil Omega-3 fatty acids Mood Stabilizers Antidepressants	25
**Reproducibility of previous findings by us**														
Top finding from Niculescu et al. [30]														
**GRK3** G Protein-Coupled Receptor Kinase 3	204183_s_at	(D) DE/2 40.2%	7.00	2.27E−02/4 Nominal		**Gender** **Females** **C:** (5/94) 0.78/1.95E-02 **Gender/Dx** **F-BP** **C:** (3/40) 0.91/9.74E−03	**Gender** **Females** **C:** (7/41) 0.74/2.22E-02 **Gender/Dx** **F-MDD** **C:** (3/7) 1/1.69E−02 **M-MDD** **L:** (6/31) 0.73/4.01E-02	**Gender/Dx** **F-BP** **C:** (4/13) 3.96/2.02E−02 **M-BP** **C:** (26/83) 1.36/4.35E-02 **M-MDD** **L:** (10/33) 2.02/1.26E−02	**Gender/Dx** **M-PTSD** **L:** (3/9) 1/1.01E-02			**Gender/Dx** **M-SZ** **C:** (6/55) 2.58/2.82E−02 **L:** (4/31) 3.58/3.82E-02	Haloperidol	20
Top Finding from LE−Niculescu et al. [1]														
**FGFR1** Fibroblast Growth Factor Receptor 1	211535_s_at	(D) DE/4 56.5%	10	NS	**Gender/Dx** **M-PTSD** **C:** (6/19) 0.81/1.76E-02 **L:** (2/9) 1/2.02E−02	**ALL** **C:** (40/485) 0.58/4.55E-02 **L:** (15/259) 0.64/3.11E−02 **Gender** **Males** **C:** (35/391) 0.6/2.72E-02 **L:** (13/210) 0.68/1.55E−02 **Gender/Dx** **M-PTSD** **L:** (3/10) 0.95/1.52E-02 **M-MOOD** **L:** (2/6) 1/3.2E−02	**Gender/Dx** **M-SZA** **C:** (6/67) 0.89/9.10E-04		**Gender/Dx** **M-PTSD** **C:** (6/19) 0.74/4.78E−02 **L:** (3/9) 0.94/1.94E-02				Lithium Clozapine Fluoxetine	20
Top Finding from Patel et al. 2010														
**ARNTL** Aryl Hydrocarbon Receptor Nuclear Translocator Like	209824_s_at	(I) DE/2 43.9%	10	1.92E−01 /2 Stepwise		**Gender/Dx** **M-MOOD** **C:** (3/11) 0.96/1.24E-02 **L:** (2/6) 1/3.2E−02	**Gender/Dx** **F-PTSD** **C:** (2/11) 0.94/2.97E-02 **F-BP** **C:** (2/13) 0.91/3.787E−02		**Gender/Dx** **F-BP** **L:** (5/16) .87/1.01E-02				Lithium Ketamine Quetiapine Fluoxetine Risperidone insulin-like growth factor-1	17

C. MANIA											
Gene symbol/Gene name	Probesets	Step 1 Discovery (direction of change in high mood) method/score/% 6 pt	Step 2 External convergent functional genomics (CFG) evidence for involvement in mood score 12pts	Step 3 Validation ANOVA *p* value/score 6 pts	Step 4 Significant predictions of high mood state ROC AUC/ p value 3 pts all 2pts gender 1pts gender /Dx	Step 4 Significant predictions of mania state ROC AUC/*p* value 3 pts all 2pts gender 1pts gender /Dx	Step 4 Significant predictions of first year hosp for mania ROC AUC/*p* value 3 pts all 2pts gender 1pts gender /Dx	Step 4 Significant predictions of all future hosp for mania OR/OR p value 3 pts all 2pts gender 1pts gender /Dx	Other psychiatric and related disorders evidence	Drugs that modulate the biomarker in opposite direction to high mood	CFE Polyevidence scorefor involvement in mania (Based on Steps 1–4)
**RPL3** Ribosomal Protein L3	212039_x_at	(I) DE/4 50%	8	3.32E−02/4 Nominal	**Gender** Females **C:**(19/82) 0.66/1.86E−02 **Gender/Dx** F-BP **C:** (9/30) 0.82/3.54E-03 **L:** (5/16) 0.85/1.36E−02		**All:** **C:** (11/332) 0.68/ 2.18E-02 **Gender:** Males **C:** (10/291) 0.66/ 3.99E−02			anisomycin	21
**SLC6A4** solute carrier family 6 (neurotransmitter transporter), member 4	242009_at	(D) DE/4 64.1%	10	5.28E-02/2 Stepwise	**Gender/Dx** F-BP **C:** (9/30) 0.73/2.45E−02 **L:** (5/16) 0.85/1.36E-02	**Gender/Dx** M-Psychosis **L:** (1/27) 1/4.76E−02	**All:** **C:** (11/332) 0.74/ 3.33E-03 **Gender:** Males **C:** (10/291) 0.72/ 8.33E−03 **Gender/Dx** M-BP **C:** (6/71) 0.77/1.35E-02 M-MDD **C:** (1/55) 1/4.45E−02	**Gender/Dx** F-BP **C:** (9/30) 0.73/2.45E−02 **L:** (5/16) 0.85/1.36E-02			21
**Reproducibility of previous findings by us**											
Top Finding from Niculescu et al. [30]											
**GRK3** G Protein-Coupled Receptor Kinase 3	204183_s_at	(D) DE/2 40.2%	7	2.27E−02/4 Nominal	**Gender/Dx** **M-PTSD** **L:** (3/9) 1/1.01E-02			**Gender/Dx** **M-SZ** **C:** (6/55) 2.58/2.82E−02 **L:** (4/31) 3.58/3.82E-02		Carbamazepine Lithium Clozapine	15
Top Finding from LE−Niculescu et al. [1]											
**FGFR1** Fibroblast Growth Factor Receptor 1	211535_s_at	(D) DE/4 56.5%	10	NS	**Gender/Dx** **M-PTSD** **C:** (6/19) 0.74/4.78E-02 **L:** (3/9) 0.94/1.94E−02					Haloperidol	15
Top Finding from Patel et al. 2010											
**ARNTL** Aryl Hydrocarbon Receptor Nuclear Translocator Like	209824_s_at	(I) DE/2 43.9%	10	1.92E-01 /2 Stepwise	**Gender/Dx** **F-BP** **L:** (5/16) .87/1.01E−02					Ketamine Risperidone Fluoxetine Lithium	15

Based on the totality of evidence from our studies (discovery, prioritization, validation, and testing for low mood/depression and for high mood/mania). We also show what the evidence/reproducibility looks like for 3 other top candidate genes from previous publications by us

*DE* differential expression, *AP* absent/present, *NS* non-stepwise in validation. For Step 4 predictions, *C* cross-sectional (using levels from one visit), *L* longitudinal (using levels and slopes from multiple visits). In All, by gender, and personalized by gender and diagnosis. Score for predictions: 3 pts if in all, 2 pts gender, 1 pts gender/Dx. Underlined—best predictive biomarker for that phenotype and population. *M* Males, *F* Females. *MDD* depression, *BP* bipolar, *SZ* schizophrenia, *SZA* schizoaffective, *PSYCHOSIS* schizophrenia and schizoaffective combined, *PTSD* post-traumatic stress disorder.

## Materials and methods

### Cohorts

We used three independent cohorts: (1) discovery (a longitudinal psychiatric subjects cohort with diametric changes in mood state from at least two consecutive testing visits); (2) validation (an independent psychiatric subjects cohort with clinically severe depression or mania); and (3) testing (an independent psychiatric subjects test cohort for predicting mood state, clinical depression or mania, and for predicting future hospitalizations for depression or mania) (Fig. 1A and Table 1).

Similar to our previous studies in suicide [3–5], the live psychiatric subjects are part of a larger longitudinal cohort of adults that we are continuously collecting. Subjects are recruited primarily from the patient population at the Indianapolis VA Medical Center. All subjects understood and signed informed consent forms detailing the research goals, procedure, caveats and safeguards, per IRB approved protocol. Subjects completed diagnostic assessments by structured clinical interviews. They had an initial testing visit in the lab or on the inpatient psychiatric unit, followed by up to six testing visits, 3–6 months apart or whenever a new psychiatric hospitalization occurred. At each testing visit, they received a series of psychiatric rating scales, and their blood was drawn. The rating scales included the Hamilton Rating Scale for Depression-17 (HAMD), the Young Mania Rating Scale (YMRS), and a visual analog scale for assessing mood state (SMS-7), which provides a score that is the average of seven items (Fig. S1A), and is part of the SASS (Niculescu et al. [12], Niculescu et al. [4], Levey et al. [5], Niculescu et al. [6]). SMS-7 integrates on a continuum in a quantitative fashion clinical symptoms for depression and mania, and provides a score for mood state at a particular moment in time. This is a state measure, related to how people feel in the present. It has good face validity based on DSM criteria, and correlates inversely with HAMD [12] (Fig. S1B). SASS, in addition to seven items measuring mood (SMS-7), also has four items measuring anxiety (SAS-4). We also used the PANSS Positive scale, that measures positive psychotic symptoms. These last two measures (SAS-4 and PANSS Positive) may define subtypes of low mood, as shown in the Discovery cohort (Fig. S1E).

We also created and used a checklist/measure of clinical severity of bipolar disorder, based on past history, called Convergent Functional Information for Bipolar Disorder Severity (CFI-BP) scale, ranking patients with mood disorders on a scale of 1–10. This is a trait measure, related to how people behaved in their past (Fig. S2).

At each visit, we collected whole blood (5 ml) in two RNA-stabilizing PAXgene tubes, labeled with an anonymized study ID number, and stored at −80 °C in a locked freezer until the time of future processing. Whole-blood RNA was extracted for microarray gene expression studies from the PAXgene tubes, as detailed below.

For this study, our within-subject discovery cohort, from which the biomarker data were derived, consisted of 44 subjects (30 males, 14 females) with psychiatric disorders and multiple testing visits, who each had at least one diametric change in SMS-7 mood scores from low mood (SMS-7 ≤ 40) to high mood (SMS-7 ≥ 60), or vice versa, from one testing visit to another. There were 4 subjects with 6 visits each, 6 subjects with 4 visits each, 18 subjects with 3 visits each, and 16 subjects with 2 visits each resulting in a total of 134 blood samples for subsequent gene expression microarray studies (Fig. 1, Tables 1 and S1).

Our independent validation cohort, in which the top biomarker findings were validated for being even more changed in expression, consisted of 39 male and 8 female subjects with a clinically severe mood disorder (*n* = 30 depression as measured by HAMD scores ≥22, and *n* = 17 mania as measured by YMRS scores ≥20), and concordant low mood, respectively high mood, SMS-7 scores (Tables 1 and S1).

Our independent test cohort for predicting low-mood state (SMS-7 ≤ 40) and high-mood state (SMS-7 ≥ 60) consisted of 153 male and 37 female subjects with psychiatric disorders, demographically matched with the discovery cohort, with one or multiple testing visits in our study, with either low mood, intermediate mood, or high mood states (Fig. 1 and Table 1).

Our independent test cohort for predicting clinical depression state (HAMD ≥ 22) consisted of 181 male and 45 female subjects with psychiatric disorders, demographically matched for age, with one or multiple testing visits in our study, with either low, intermediate, or high HAMD scores. Our independent test cohort for predicting clinical mania state (YMRS ≥ 20) consisted of 73 males and 24 female subjects with psychiatric disorders, demographically matched for age, with one or multiple testing visits in our study, with either low, intermediate, or high YMRS scores (Fig. 1 and Table 1).

Our test cohorts for predicting future hospitalizations with depression, and future hospitalizations with mania (Fig. 1 and Table 1), are a subset of the independent test cohort for which we had longitudinal follow-up with electronic medical records. The subjects’ subsequent number of hospitalizations with depression, and with mania, was tabulated from electronic medical records.

#### Medications

The subjects in the discovery cohort were all diagnosed with various psychiatric disorders (Table 1), and had various medical co-morbidities. Their medications were listed in their electronic medical records, and documented by us at the time of each testing visit. Medications can have a strong influence on gene expression. However, there was no consistent pattern of any particular type of medication, as our subjects were on a wide variety of different medications, psychiatric and non-psychiatric. Furthermore, the independent validation and testing cohort’s gene expression data were *Z*-scored by gender and by diagnosis before being combined, to normalize for any such effects. Some subjects may be non-compliant with their treatment and may thus have changes in medications or drug of abuse not reflected in their medical records. That being said, our goal is to find biomarkers that track mood, regardless if the reason for it is endogenous biology or it is driven by medications or drugs. In fact, one would expect some of these biomarkers to be targets of medications, as we show in this paper. Moreover, the prioritization step that occurs after discovery is based on a field-wide convergence with literature that includes genetic data and animal model data, that are unrelated to medication effects. Overall, the discovery, validation, and replication by testing in independent cohorts of the biomarkers, with our design, occurs despite the subjects having different genders, diagnoses, being on various different medications, and other lifestyle variables.

### Blood gene expression experiments

#### RNA extraction

Whole blood (2.5 ml) was collected into each PaxGene tube by routine venipuncture. PaxGene tubes contain proprietary reagents for the stabilization of RNA. RNA was extracted and processed as previously described [3–5].

#### Microarrays

Microarray work was carried out using previously described methodology [3–6].

Of note, all genomic data were normalized (RMA for technical variability, then *z*-scoring for biological variability), by gender and psychiatric diagnosis, before being combined and analyzed.

### Biomarkers

#### Step 1: Discovery

We have used the subject’s score from a visual-analog scale (SMS-7), assessed at the time of blood collection (Fig. 1). We analyzed gene expression differences between visits with low mood (defined as a score of 0–40) and visits with high mood (defined as a score of 60–100), using a powerful within-subject design, then an across-subjects summation (Fig. 1).

We analyzed the data in two ways: an absent–present (AP) approach, and a differential expression (DE) approach, as in previous work by us on suicide biomarkers [3–5]. The AP approach may capture turning on and off of genes, and the DE approach may capture gradual changes in expression. Analyses were performed as previously described [4–6]. In brief, we imported all Affymetrix microarray data as CEL. files into Partek Genomic Suites 6.6 software package (Partek Incorporated, St Louis, MI, USA). Using only the perfect match values, we ran a robust multi-array analysis (RMA) by gender and diagnosis, background corrected with quantile normalization and a median polish probeset summarization of all chips, to obtain the normalized expression levels of all probesets for each chip. Then, to establish a list of differentially expressed probesets we conducted a within-subject analysis, using a fold change in expression of at least 1.2 between consecutive high- and low-mood visits within each subject. Probesets that have a 1.2-fold change are then assigned either a 1 (increased in high mood) or a −1 (decreased in high mood) in each comparison. Fold changes between 1.1 and 1.2 are given 0.5, and fold changes less than 1.1 are given 0. These values were then summed for each probeset across all the comparisons and subjects, yielding a range of raw scores. The probesets above the 33.3% of raw scores were carried forward in analyses (Fig. 1), and received an internal score of 2 points; those above 50% 4 points, and those above 80% 6 points [4–6]. We have developed in our labs R scripts to automate and conduct all these large dataset analyses in bulk, checked against human manual scoring [6].

Gene Symbol for the probesets were identified using NetAffyx (Affymetrix) for Affymetrix HG-U133 Plus 2.0 GeneChips, followed by GeneCards to confirm the primary gene symbol. In addition, for those probesets that were not assigned a gene symbol by NetAffyx, we used GeneAnnot (https://genecards.weizmann.ac.il/geneannot/index.shtml), or if need be UCSC (https://genome.ucsc.edu), to obtain gene symbol for these uncharacterized probesets, followed by GeneCard. Genes were then scored using our manually curated convergent functional genomics (CFG) databases as described below (Fig. 1D).

#### Step 2: Prioritization using CFG

##### Databases

We have established in our laboratory (Laboratory of Neurophenomics, www.neurophenomics.info) manually curated databases of the human gene expression/protein expression studies (postmortem brain, peripheral tissue/fluids: CSF, blood and cell cultures), human genetic studies (association, copy number variations and linkage), and animal model gene expression and genetic studies, published to date on psychiatric disorders. Only findings deemed significant in the primary publication, by the study authors, using their particular experimental design and thresholds, are included in our databases. Our databases include only primary literature data and do not include review papers or other secondary data integration analyses to avoid redundancy and circularity. We also favored unbiased discovery studies over candidate genes hypothesis-driven studies. These large and constantly updated databases have been used in our CFG cross validation and prioritization platform (Fig. 1D). For this study, data from 1600 papers on mood disorders were present in the databases at the time of the CFG for mood disorders analyses (June 2018) (human genetic studies-759, human brain studies-246, human peripheral tissue/fluids- 359, non-human genetic studies-47, non-human brain/studies-167, non-human peripheral tissue/fluids- 22). We have developed in our lab a computerized CFG Wizard to automate and score in bulk large lists of genes by integrating evidence from these large databases, checked against manual scoring [6]. Analyses were performed as previously described [4, 5].

#### Step 3: Validation analyses

We examined which of the top candidate genes (score of 6 or above after the first two steps) were stepwise changed in expression from the clinically depressed validation group to the low-mood discovery group to the high-mood discovery group to the clinically manic validation group. A total score of 6 or above after the first two steps permits the inclusion of potentially novel genes with maximal internal score of 6 from discovery but no external evidence CFG score from prioritization. Subjects with low mood as well as subjects with high mood from the discovery cohort who did not have clinically severe depression or mania were used, along with the independent validation cohort (*n* = 47).

The AP-derived and DE-derived lists of genes were combined, and the gene expression data corresponding to them was used for the validation analysis. The four groups (clinical depression, low mood, high mood, clinical mania) were assembled out of Affymetrix.cel data that were RMA normalized by gender and diagnosis. We transferred the log transformed expression data to an Excel sheet, and non-log transformed the data by taking 2 to the power of the transformed expression value. We then *Z*-scored the values by gender and diagnosis. We then imported the excel sheets with the *Z*-scored by gender and diagnosis expression data into Partek, and statistical analyses were performed using a one-way ANOVA for the stepwise changed probesets, and also did a stringent Bonferroni correction for all the probesets tested in ANOVA (Fig. 1E).

### Top candidate biomarkers (after the first 3 steps)

Adding the scores from the first three steps into an overall convergent functional evidence (CFE) score (Fig. 1), we ended up with a list of 26 top candidate biomarkers (26 probesets in *n* = 23 genes), that had evidence, i.e., a CFE score, as good as or better than SLC6A4 (the serotonin transporter) (see also Supplementary Information- Pathways, Predictions and Reproducibility). SLC6A4 is arguably the most well studied molecular underpinning of mood disorders in biological psychiatry, and the target of the majority of antidepressant medications. We discovered it empirically as a blood biomarker as part of our work, and used it as a de facto positive control and cutoff. These 26 top candidate biomarkers were carried forward into additional analyses for biological understanding and for clinical utility.

### Biological understanding

#### Clock gene database

We compiled a database of genes associated with circadian function, by using a combination of review papers [13, 14] and searches of existing databases CircaDB (http://circadb.hogeneschlab.org), GeneCards (http://www.genecards.org), and GenAtlas (http://genatlas.medecine.univ-paris5.fr). Using the data we compiled from these sources we identified a total of 1468 genes that show circadian functioning. We further classified genes into “core” clock genes, i.e., those genes that are the main engine driving circadian function (*n* = 18), “immediate” clock genes, i.e., the genes that directly input or output to the core clock (*n* = 331), and “distant” clock genes, i.e., genes that directly input or output to the immediate clock genes (*n* = 1119).

#### Pathway analyses

IPA (Ingenuity Pathway Analysis, version 24390178, Qiagen), David Functional Annotation Bioinformatics Microarray Analysis (National Institute of Allergy and Infectious Diseases) version 6.7 (August 2016), and Kyoto Encyclopedia of Genes and Genomes (KEGG) (through DAVID) were used to analyze the biological roles, including top canonical pathways and diseases (Table 2). We performed the pathway analyses for the 26 biomarkers (23 unique genes) that were the top candidate biomarkers after the discovery, prioritization, and validation.

#### Networks

For network analyses we performed STRING Interaction network (https://string-db.org) by inputting the genes into the search window, and performed Multiple Proteins Homo sapiens analysis (Fig. S3).

#### CFG beyond mood: evidence for involvement in other psychiatric and related disorders

We also used a CFG approach to examine evidence from other psychiatric and related disorders, as exemplified for the list of top biomarkers after Step 4 testing (Table S3). This was not used to prioritize genes, but rather to understand the molecular basis of clinical co-morbidities.

### Testing for clinical utility in independent cohorts

We tested in independent cohorts of psychiatric patients the ability of each of the top candidate biomarkers (n = 26) to assess state severity (mood (measured by SMS-7), depression (measured by HAMD), mania (measured by YMRS)), and predict trait risk (future hospitalizations with depression, future hospitalizations with mania). We conducted our analyses across all patients, as well as personalized by gender and diagnosis. We then predict with the biomarkers from the list in independent cohorts state (low-mood SMS-7 ≤ 40, depression HAMD ≥ 22), and trait (Future Hospitalizations with Depression) in the first year of follow-up, and in all future years of follow-up. We also conducted similar analyses for predicting high mood, mania, and future hospitalizations for mania.

The test cohort for predicting low mood/depression(state), and the test cohort for predicting future Hospitalizations with Depression (trait), was assembled out of data that were RMA normalized by gender and diagnosis. The cohort was completely independent from the discovery and validation cohorts, there was no subject overlap with them. Individual markers used for predictions were *Z*-scored by gender and diagnosis, to be able to combine different biomarkers into panels and to avoid potential artefacts due to different ranges of expression in different gender and diagnoses. For panels, biomarkers were combined by simple summation of the increased risk biomarkers minus the decreased risk biomarkers. Predictions were performed using R-studio. For cross-sectional analyses, we used biomarker expression levels, *z*-scored by gender and diagnosis. For longitudinal analyses, we combined four measures: biomarker expression levels, slope (defined as ratio of levels at current testing visit vs. previous visit, divided by time between visits), maximum levels (at any of the current or past visits), and maximum slope (between any adjacent current or past visits). For decreased biomarkers, we used the minimum rather than the maximum for level calculations. All four measures were *Z*-scored, then combined in an additive fashion into a single measure. The longitudinal analysis was carried out in a sub-cohort of the testing cohort consisting of subjects that had at least two visits (timepoints).

#### Predicting state- low mood, clinical depression

Receiver-operating characteristic (ROC) analyses between marker levels and mood state were performed by assigning subjects visits with a mood SMS-7 score of ≤40 into the low mood category, and subjects with HAMD scores ≥22 in the clinically depressed category. We used the pROC package of R (Xavier Robin et al. BMC Bioinformatics 2011). (Table 3 and Fig. 2). In addition, a one-tailed t-test was performed between low mood group vs. the rest, and Pearson R (one-tail) was calculated between mood scores and biomarker levels.

Similar analyses were conducted for high mood state (SMS-7 score of ≥60) and clinical mania state (YMRS ≥ 20).

#### Predicting trait- future psychiatric hospitalization with depression as a symptom/reason for admission

We conducted analyses for predicting future psychiatric hospitalizations with depression as a symptom/reason for admission in the first year following each testing visit, in subjects that had at least 1 year of follow-up in the VA system, in which we have access to complete electronic medical records. ROC analyses between biomarkers measures (cross-sectional, longitudinal) at a specific testing visit and future hospitalizations were performed as described above, based on assigning if subjects had been admitted to the hospital with depression or not. In addition, a one tailed t-test with unequal variance was performed between groups of subject visits with and without future hospitalization with depression. Pearson R (one-tail) correlation was performed between hospitalization frequency (number of hospitalizations with depression divided by duration of follow-up) and marker levels. A Cox regression was performed using the time in days from the testing visit date to first hospitalization date in the case of patients who had been hospitalized, or 365 days for those who did not. The odds ratio (OR) was calculated such that a value greater than 1 always indicates increased risk for hospitalization, regardless if the biomarker is increased or decreased in expression.

We also conducted Cox regression and Pearson *R* analyses for all future hospitalizations with depression, including those occurring beyond 1 year of follow-up, in the years following testing (on average 5.12 years per subject, range 0.07–11.27 years), as these calculations, unlike the ROC and t-test, account for the actual length of follow-up, which varied from subject to subject. The ROC and *t*-test might in fact, if used, under-represent the power of the markers to predict, as the more severe psychiatric patients are more likely to move geographically and/or be lost to follow-up. The Cox regression was performed using the time in days from visit date to first hospitalization date in the case of patients who had hospitalizations with depression, or from visit date to last note date in the electronic medical records for those who did not.

Similar analyses were conducted for future hospitalizations with mania as a symptom/reason for hospitalization.

### Therapeutics

#### Pharmacogenomics

We analyzed which of the top biomarkers for depression and for mania after Steps 1–4 are known to be changed in expression by existing drugs in a direction opposite to the one in disease, using our CFG databases, and using Ingenuity Drugs analyses (Table 3 and Table S4).

#### New drug discovery/repurposing

**Fig. 3:**
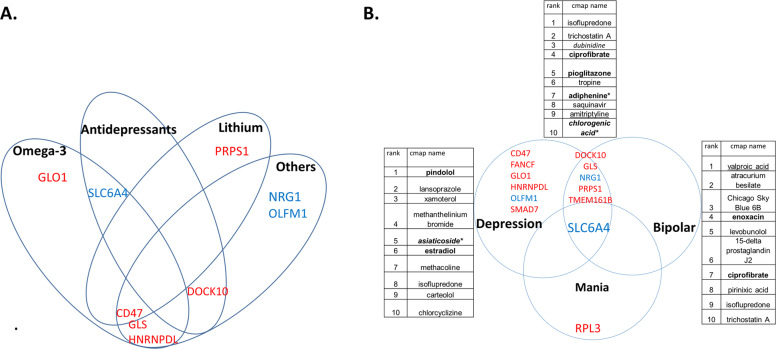
Therapeutics: matching with medications. **A** Pharmacogenomics. See also Tables 3 and S4. **B** New repurposed drugs using the panels of markers. See also Table 4. There is overlap between depression, bipolar and mania biomarkers. RPL3 could be targeted to treat mania with less risk of inducing depression. Six biomarkers (CD47, FANCF, GLO1, HNRNPDL, OLFM1, SMAD7) could be targeted to treat depression with less risk of inducing mania. Other six biomarkers (DOCK10, GLS, NRG1, PRPS1, TMEM161B, SLC6A4) could be targeted to treat depression fast/powerfully, but may induce mania, so need to be coupled with a mood stabilizer or antipsychotic. An example of the latter is SLC6A4. SSRIs should thus be used cautiously in monotherapy to treat depression, and clinicians should have a low threshold for adding mood stabilizers.

We also analyzed which drugs and natural compounds are an opposite match for the gene expression signatures of our top biomarkers, using the Connectivity Map (https://portals.broadinstitute.org, Broad Institute, MIT) (Fig. 3 and Table 4). Of note, not all the probesets from the HG-U133 Plus 2.0 array we used were present in the HGU-133A array used for the Connectivity Map. We stayed with exact probeset level matches, not gene level imputation. We also used the NIH LINCS database to conduct similar analyses, at a gene level.

**Table 4:** Therapeutics: drug repurposing for depression.

A. Connectivity Map (CMAP) analyses			
Rank	CMAP name	Score	Role
**A1. Drugs identified using gene expression panels of biomarkers with highest evidence (CFE) for involvement in depression (BioM12 depression—12 genes—NRG1, PRPS1, GLS, DOCK10, TMEM161B, GLO1, HNRNPDL, FANCF, CD47, SMAD7, OLFM1, SLC6A4). See Table** 3A**and Fig.** 3**. Direction of expression in high mood (CMAP).**			
1	Isoflupredone	1	Synthetic glucocorticoid that may be considered as an alternative to traditional corticosteroids. Isoflupredone is the only corticosteroid approved by the U.S. Food and Drug Administration for use exclusively in large animals, including lactating cattle.
2	Trichostatin A	0.963	HDAC inhibitor
3	*Dubinidine*	0.943	Anticonvulsant which reduces motor activity, enhances the effects of alcohol, ether and barbiturates. Quinoline alkaloid, from plants of the Rutaceae Family.
4	**Ciprofibrate**	0.939	PPAR-alpha activator, lipid lowering agent
5	**Pioglitazone**	0.931	PPAR-γ activator, anti-diabetic (*also in our work on Alzheimer [10])
6	tropine	0.93	Alkaloid
7	Adiphenine*	0.907	Anticholinergic, antispasmodic (*also in our work on suicidality [6])
8	Saquinavir	0.903	Anti-retroviral medication
9	Amitriptyline	0.902	Tricyclic antidepressant.
10	Chlorogenic acid*	0.897	Antioxidant, polyphenol found in coffee (*also in our work on suicidality [6])

**A2. Drugs identified using gene expression panels of biomarkers with highest evidence (CFE) for involvement in depression specific without overlap with bipolar (BioM6 Depression-specific—6 genes—GLO1, HNRNPDL, FANCF, CD47, SMAD7, OLFM1). Direction of expression in high mood (CMAP). See Fig.** **3**.			
1	**Pindolol**	1	β-blocker, and is also a potent serotonin 5HT_1A_ presynaptic receptor antagonist
2	Lansoprazole	0.977	Proton pump inhibitor (PPI), that works by decreasing the amount of acid produced by the stomach.
3	Xamoterol	0.975	Cardiac stimulant, that works by binding to the β_1_ adrenergic receptor. It is a 3rd generation adrenergic β receptor partial agonist. It provides cardiac stimulation at rest but it acts as a blocker during exercise.
4	Methanthelinium bromide	0.953	Muscarinic receptor antagonist (anticholinergic, parasympatholytic agent). Spasmolytic agent. Gastric acid secretion inhibitor.
5	Asiaticoside*	0.927	Triterpenoid component derived from Centella asiatica (L.) and widely used in antioxidant, anti-inflammatory, immunomodulatory, and wound healing applications. (*also in our work on suicidality [6])
6	**Estradiol**	0.924	Female sex hormone
7	Methacholine	0.923	Muscarinic agonist
8	Isoflupredone	0.916	Steroid
9	Carteolol	0.913	Beta blocker
10	Chlorcyclizine	0.911	First-generation antihistamine. It is used primarily to treat allergy symptoms such as rhinitis, urticaria, and pruritus, and may also be used as an antiemetic.

**A3. Drugs identified using gene expression panels of biomarkers overlapping between depression and bipolar (BioM6 bipolar depression—6 genes—NRG1, DOCK10, GLS, PRPS1, TMEM161B, and SLC6A4). Direction of expression in high mood. (CMAP). See Table** 3B **and Fig**. 3.			
1	Valproic acid	1	HDAC inhibitor, mood stabilizer
2	Atracurium besilate	0.991	Nicotinic antagonist muscle relaxant
3	Chicago Sky Blue 6B	0.98	Histological stain that also is a vesicular glutamate transporters inhibitor, attenuating methamphetamine-induced hyperactivity and behavioral sensitization in animal models
4	**Enoxacin**	0.972	Fluoroquinolone antibiotic that also elevates microRNA levels and prevents learned helplessness in animal models
5	Levobunolol	0.969	Beta-blocker
6	15-delta prostaglandin J2	0.95	Anti-inflammatory lipid mediator and PPAR-γ activator. It is made from prostaglandin D2. Decreased Prostaglandin D2 Levels in Major Depressive Disorder Are Associated with Depression-Like Behaviors in human and animal model studies.
7	**Ciprofibrate**	0.949	PPAR-alpha activator, lipid lowering agent
8	Pirinixic acid	0.949	PPAR-alpha activator, anti-lipid agent
9	Isoflupredone	0.947	Synthetic glucocorticoid
10	Trichostatin A	0.946	HDAC inhibitor

B. NIH LINCS L1000 characteristic direction signature search engine analyses			
Rank	Score	Drug	Description
**B1. Drugs identified using gene expression panels of biomarkers with highest evidence (CFE) for involvement in depression (BioM12 Depression- 12 genes). See Table** 3A**and Fig.** 3**. Direction of expression in high mood (9 increased and 3 decreased).**			
1	0.3	NNC 55–0396 dihydrochloride	T-type calcium channel blocker
2	0.3	**Nadolol**	Beta blocker
3	0.3	MLN4924	Inhibitor of Nedd8-Activating Enzyme
4	0.2	U0126	MEK ½ inhibitor
5	0.2	Nortryptiline	Tricyclic antidepressant
6	0.2	Amcinonide	Synthetic glucocorticoid
7	0.2	Iopanic acid	Iodine-containing radiocontrast medium, thyroid inhibitor
8	0.2	Paroxetine	SSRI antidepressant
9	0.2	**Rosuvastatin**	Statin
10	0.2	trichostatin A	HDAC inhibitor

Drugs that have *opposite* gene expression effects to the gene expression signature of our nominally significant predictive biomarkers for depression(A1–A2) and for bipolar depression(A3), using the Connectivity Map [36] (CMAP), and for depression (B1) using the NIH LINCS database. Bold—new drugs of immediate interest. Italic—natural compound. Underlined—known drugs that serve as a de facto positive control.

### Report generation

**Fig. 4:**
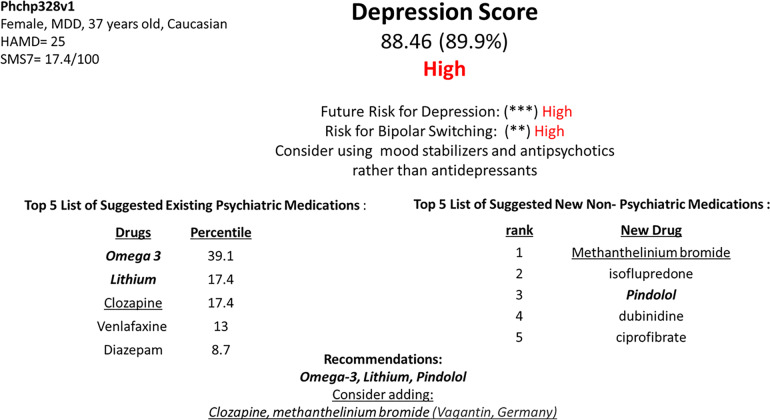
Example of report for physicians. Using the panel of the top biomarkers BioM12 + 1: Depression (*n* = 12 genes), as well as RPL3 for mania risk. This subject (Phchp328) was previously described by us in a suicidality biomarker study (Levey et al. [5]), as high risk for suicide, and died by suicide a year after completing our study. No information was provided to her clinicians by us at that time due to anonymity and privacy rules in research studies. The raw expression values of the biomarkers were *Z*-scored by gender and diagnosis. The *Z*-scored expression value of each increased biomarker was compared to the average value for the biomarker in the severely depressed group (HAMD ≥ 22), and the average value of the non-depressed group (HAMD ≤ 7), resulting in scores of 1 or 0, respectively, and 0.5 if it was in between. The reverse was done for decreased biomarkers. The “digitized” biomarkers were then added into a polygenic risk score. The subject had a BioM12 polygenic depression score of 88.46, being at the 90% of the 794 subjects in our database. Three out of the three biomarkers for future risk for depression hospitalizations (NRG1, PRPS1, SMAD7) had a score of 1 in this patient (***). More than 50% of the 6 bipolar biomarkers that are part of the BioM12 (Table 3A and B) (*), as well as the mania marker RPL3 (Table 3C) (*), had a score of 1 in this patient, resulting in increased risk for bipolar switching (**). This subject’s clinical diagnosis was major depressive disorder (MDD), but it is likely she had bipolar disorder. The “digitized” biomarkers were also used for matching with existing psychiatric medications. Biomarkers were matched based on our CFG databases with existing psychiatric medications that had effects on gene expression opposite to depression, in the direction of high mood. Each medication matched to a biomarker got a score of 1 that was then multiplied with the biomarker score of 1, 0.5 or 0. The scores for the medications were added, and medications prioritized by this score. In addition, the signature of the biomarkers in the panel that had a score of 1, and their direction of change, was used to interrogate the CMAP and LINCS databases for new repurposed medications that would treat depression in this patient.

We present an example of how a report to doctors might look, using the above insights. We used a panel of top biomarkers after Steps 1–4 (Fig. 3 and Table 3): BioM12 + 1: *n* = 12 genes to generate a score for depression severity, as well as the mania biomarker RLP3 to inform risk for bipolar switching. Out of a dataset of 794 subject visits, we chose as a case study a visit from a female patient with depression who had died by suicide, a case previously discussed in a suicide biomarker paper of ours (Levey et al. [5]) (Fig. 4).

First, we removed that patient from the dataset, and divided the remaining dataset into three populations: those who had a high HAMD score ≥22 (concordant with a low SMS-7 mood score ≤ 40), those who had a low HAMD score ≤7, and those who had an intermediate HAMD score. For the first two groups, we calculated the average *Z*-scored expression values for each biomarker in the panel. We then compared the levels of each biomarker, in each subject in the cohort, including the subject of interest, to these reference levels. If a biomarker was higher than the average of the high HAMD subjects it got a 1, if it was below the average of the low HAMD subjects it got a 0, and if it was in between it got a 0.5. Next, we averaged the biomarkers in the panel and multiplied by 100, to generate a score between 0 and 100 for the BioM12 for each of the 794 subjects, including the case study subject. This digitalization of the scores was done to avoid overfitting to our particular cohort, and provide an easily understandable and interpretable readout for clinicians. The score of the BioM12 is compared to the average score of BioM12 for the high HAMD subjects and the low HAMD subjects, generating 3 risk categories: high (red), intermediate (yellow), and low (green) for current depression severity. This rank percentile of the score of the patient compared to the distribution of scores of subjects in the database is also provided in the report (Fig. 4).

Second, future risk is assessed by looking how many of three biomarkers in the panel, that are good predictors of future hospitalizations for depression (NRG1, PRPS1, SMAD7), were a 1, generating 0 to 3 asterisks.

Third, we examined how many of the bipolar biomarkers (*n* = 6) in BioM12 were a score of 1. If more than 50% of them (more than 3 out of 6) were a 1, than the patient gets an asterisk for bipolar risk. If the mania biomarker RLP3 is also 1, then the patient gets another asterisk for risk of bipolarity, i.e., risk of switch if treated for depression. In those with asterisks for risk of bipolarity, it is advisable to choose mood stabilizers or antipsychotics from the medication choices provided by the report.

Fourth, for each biomarker in the panel, we also have a list of existing psychiatric medications that modulate the expression of the biomarker in the direction of high mood. Each medication got a score of 1 or 0 whether it modulated a particular biomarker in the panel or not, and that score is multiplied with the risk score of the biomarker, i.e., 1 or 0.5 or 0. A medication can modulate more than one biomarker. We then calculated an average score for each medication based on its effects on all the biomarkers in the panel, and multiplied that by 100, resulting in a score of 0 to 100 for each medication. Thus, psychiatric medications are matched to the patient and ranked in order of impact on the panel.

Fifth, we used the biomarkers that were positive as high risk in the panel, to interrogate the CMAP and do individualized drug repurposing, identifying new non-psychiatric compounds that could be used in that particular patient to treat depression (Fig. 4).

## Results

In Step 1 Discovery, we identified candidate blood gene expression biomarkers that: (1) change in expression in blood between self-reported low-mood and high-mood states, (2) track the mood state across visits in a subject, and (3) track the mood state in multiple subjects. We used a visual analog measure for mood state (SMS-7). At a phenotypic level, the SMS-7 quantitates mood state at a particular moment in time, and normalizes mood measurements in each subject, comparing them to the lowest and highest mood that subject ever experienced. We then used a powerful within-subject and then across-subject design in a longitudinally followed cohort of subjects (*n* = 44 subjects, with 134 visits) who displayed at least a 50% change in the mood measure (from below 40/100 to above 60/100) between at least two consecutive testing visits, to identify differentially expressed genes that track mood state. Using our 33% of maximum raw score threshold (internal score of 2 pt) [4, 5], we had 11,620 unique probesets (corresponding to 9649 unique genes) from Affymetrix Absent/Present (AP) analyses and DE analyses (Fig. 1D). These were carried forward to the prioritization step. This represents approximately a fivefold enrichment of the 54,625 probesets on the Affymetrix array.

We also examined in the discovery cohort whether subtypes of low mood can be identified based on mental state at the time of low mood visits, using two-way hierarchical clustering with anxiety and psychosis measures. The mood state self-report may be more reliable in this cohort, as the subjects demonstrated the aptitude and willingness to report different, and diametric, mood states. We uncovered four potential subtypes of low mood/depression: high anxiety and low psychosis (anxious), high anxiety and high psychosis (combined), low anxiety and high psychosis (psychotic), low anxiety and low psychosis (pure low mood) (Fig. S3). These subtypes need to be tested in future studies in independent cohorts for practical utility, diagnostic and therapeutic.

In Step 2 Prioritization, we used a CFG approach to prioritize the candidate biomarkers identified in the discovery step (33% cutoff, internal score of ≥2 pt.) by using published literature evidence (genetic, gene expression and proteomic), from human and animal model studies, for involvement in mood disorders (Fig. 1E and Table S2). There were 6370 probesets (corresponding to 4960 unique genes) that had a total score (combined discovery score and prioritization CFG score) of 6 and above. These were carried forward to the validation step. This represents approximately a tenfold enrichment of the probesets on the Affymetrix array.

In Step 3 Validation, we validated for change in clinically severe mood disorders (depression, mania) these prioritized biomarkers, in a demographically matched cohort of (*n* = 30 clinically severe depression, n = 17 clinically severe mania), by assessing which markers were stepwise changed in expression: from clinically severe depression in validation cohort, to low mood in discovery cohort, to high mood in discovery cohort, and to clinically severe mania in the validation cohort (Fig. 1). Four thousand six hundred thirty-three probesets were not stepwise changed, and 1737 were stepwise changed. Of these, 291 probesets (corresponding to 283 unique genes) were nominally significant. This represents approximately a 188-fold enrichment of the probesets on the Affymetrix array.

Adding the scores from the first three steps into an overall CFE score (Fig. 1), we ended up with a list of 26 top candidate biomarkers (*n* = 23 genes, 26 probesets), that had a CFE score as good as or better than SLC6A4, which serves as a de facto positive control and which we decided to use as an empirical cutoff. This represents approximately an over 2000-fold enrichment of the probesets on the Affymetrix array.

The list of 23 genes (26 probesets) (Fig. 1) is composed of genes increased in expression in high mood (TMEM161B, GLO1, PRPS1, SMAD7, ANK3, OGT, CD47, GLS, TMEM106B, RPL3, FANCF, HNRNPDL, DOCK10, CALM1), and genes decreased in expression in high mood (NRG1, OLFM1, SPECC1, SORT1, TPH1, GSK3B, MARCKS, NR3C1, and SLC6A4). These 26 top candidate biomarkers were carried forward into analyses for understanding biological underpinnings. Last but not least, they were tested for predictive ability and clinical utility in additional independent cohorts.

### Biological understanding

#### Biological pathways

We conducted biological pathway analyses using the top candidate biomarkers for mood (*n* = 23 genes, 26 probesets), which suggest that circadian, neurotrophic, and cell differentiation functions are involved, along with serotonergic and glutamatergic signaling, supporting a view of mood as reflecting activity and growth (Table 2A). Reassuringly, depression, along with weight gain, were the top diseases identified by the pathway analyses using DAVID, and Ingenuity identified neurological and psychological disorders as the top diseases.

#### Circadian

A number of top candidate biomarkers identified by us have biological roles that are related to the circadian clock (8 out of 23 genes) (Supplementary Information- Pathways, Predictions and Reproducibility). Circadian clock abnormalities are related to mood disorders [14, 15].

#### Networks and interactions

We conducted STRING analyses of the top candidate biomarkers that revealed groups of interacting proteins. In particular, NR3C1 ((Nuclear Receptor Subfamily 3, Group C, Member 1 (Glucocorticoid Receptor)) is at the overlap of a network containing SLC6A4 and TPH1, and one centered on GSK3B that also contains OGT and CALM1 (Fig. S3). A third network includes CALM1, GLO1, and MARCKS. These networks may have biological significance and could be targeted therapeutically. The first network is involved in reactivity (serotonin and stress response), the second one in activity (energy metabolism and growth), and the third one in connectivity (calcium intracellular signaling).

### Testing for clinical utility

In Step 4 Testing, we examined in completely independent cohorts from the ones used for discovery or validation whether the 26 top candidate biomarkers can assess low-mood state (*n* = 190 subjects), depression state (*n* = 226 subjects), as well as predict of future psychiatric hospitalizations due to depression (*n* = 170 subjects) (Fig. 2 and Table 3), using electronic medical records follow-up data of our study subjects (up to 11.27 years from initial visit at the time of the analyses) (Fig. 1, Table 1, and Table S1). The gene expression data in the test cohorts were normalized (*Z*-scored) across genders and various psychiatric diagnoses, before those different demographic groups were combined. We used biomarker levels information cross-sectionally, as well as expanded longitudinal information about biomarker levels at multiple visits, as predictors. We tested the biomarkers in all subjects in the independent test cohort, as well as in a more personalized fashion by gender and psychiatric diagnosis.

For low mood state assessment across all subjects in the independent test cohort, the best biomarker was NRG1, increased in expression in low mood, with an AUC of 62 % (*p* = 6.8E−03), and 64% (*p* = 3.5E−02) for assessing clinical depression state. NRG1 also had a Cox regression OR of 1.17 (*p* = 2.5E−02) for predicting all future hospitalizations for depression, and an AUC of 87% (*p* = 1.1E−03) for predicting first year hospitalizations for depression in females. Moreover, in the opposite direction, for assessing high-mood state across all subjects, NRG1 has a modest AUC of 58% (*p* = 1.4E−02), but is a robust predictor of all future hospitalizations for mania in patients with psychotic disorders (OR of 2.7 (*p* = 3.3E−02). Consistent with our findings, NRG1 (neuregulin 1) has prior evidence as a biomarker for mood disorders, increased in expression in blood in depression, and decreased in expression after antidepressant treatment [16]. Interestingly, it is increased in expression in blood in our previous biomarker studies on suicidality [6], stress [9], pain [8], and psychosis [17], as well as increased in expression in blood in aging [18], all co-morbidities associated with depression. NRG1 is a membrane glycoprotein that mediates cell–cell signaling and plays a critical role in the activity, growth and development of multiple organ systems. It is a direct ligand for ERBB3 and ERBB4 tyrosine kinase receptors, resulting in ligand-stimulated tyrosine phosphorylation and activation of the ERBB receptors. Activity and trophicity of tissues may be key functions of mood [19].

For assessment of clinical depression state in the independent test cohort, DOCK10, decreased in expression in low mood, had an AUC of 73% (*p* = 1.17E−03) across all subjects, and 75% (p = 1.05E−03) in males, surviving Bonferroni correction for all 26 biomarkers tested. It also had an AUC of 95% (*p* = 1.52E−02) for males with PTSD. DOCK10 had a Cox regression OR of 1.9 (*p* = 3.93E−02) for predicting all future hospitalizations for depression in females. Moreover, in the opposite direction, for assessing high mood state, it has an AUC of 70% in females (*p* = 2.63E−02), and 100% (*p* = 9.18E−04) in females with bipolar disorder (Table 3). DOCK10 (dedicator of cytokinesis 10) has some prior human evidence in human blood from bipolars [20], and is decreased in expression in brain in an animal model of depression [21]. DOCK10 is also decreased in expression in human brains and blood in aging [18], as well as decreased in brain in an animal model of stress-induced depression, as described by Nestler and colleagues [22]. Interestingly, it is increased in expression in brain in animal models upon physical and cognitive stimulation [23]. There is human genetic evidence implicating this gene in longevity [24]. The link between depression, stress, aging and longevity is an area of active interest for our group [25, 26]. DOCK10 is a guanine nucleotide-exchange factor that activates CDC42 and RAC1 by exchanging bound GDP for free GTP. It is essential for dendritic spine morphogenesis in Purkinje cells and in hippocampal neurons, via a CDC42-mediated pathway.

SLC6A4 is an example of a previously well-known gene reproduced in this study. For clinical depression state assessment in the independent test cohort across all subjects, SLC6A4, increased in expression in low mood, had an AUC of 61% (*p* = 1.1E−02) if measured cross-sectionally, and AUC of 66% (*p* = 1.78E−02) if measured longitudinally. It has even better accuracy in females: an AUC of 78% (*p* = 1.8E−02) if measured cross-sectionally, and an AUC of 98% (*p* = 1.1E−02) if measured longitudinally. Moreover, in the opposite direction, for predicting future hospitalizations for mania in the first year, across all subjects, SLC6A4 had an AUC of 74% (*p* = 3.3E−03), and an even better accuracy in male bipolars, with an AUC of 77% (*p* = 1.3E−02). The product of this gene is the serotonin transporter, which is the target of serotonin reuptake inhibitors used to treat depression, as well as anxiety and stress disorders. Of note, it is known that individuals with bipolar disorder treated with SSRIs, especially in monotherapy, can switch into mania.

As exemplified above, we also conducted analyses looking at the ability of the 26 top candidate biomarkers to assess high mood/mania states, and predict future hospitalizations for mania (Table 3B,C, and see Supplementary Information—Pathways, Predictions and Reproducibility).

We also tested an algorithm combining as predictors BioM26, along with mood (SMS-7, Fig. S1) and with a measure of clinical severity of bipolar disorder (CFI-BP, Fig. S2), with modest synergistic effects (Table S1). Of note, CFI-BP was a good predictor of all future hospitalizations for mania in all (Cox regression OR of 2.9 (*p* = 2.5E−04)), and an even better predictor in males with bipolar disorder (OR of 3.2 (*p* = 8.3E−05)).

### Convergent functional evidence (CFE)

For the top candidate biomarkers (*n* = 26), we tabulated into a CFE score all the evidence from discovery (up to 6 points), CFG prioritization (up to 12 points), validation (up to 6 points), and testing (state low mood, state clinical depression, trait first year hospitalization with depression, trait all future hospitalizations with depression, as well as state high mood, state clinical mania, trait first year hospitalization with mania, trait all future hospitalizations with mania—up to three points each if it significantly predicts in all subjects, two points if in gender, one points if in gender/diagnosis). The total score can be up to 48 points: 36 from our empirical data, and 12 from literature data used for CFG. We weigh our new empirical data more than the literature data, as it is functionally related to mood in three independent cohorts (discovery, validation, and testing). The goal is to highlight, based on the totality of our data and of the evidence in the field to date, biomarkers that have all around evidence: track mood, have convergent evidence for involvement in mood disorders, and predict mood state and future clinical events (Table 3). Such biomarkers merit priority evaluation in future clinical trials.

The 6 top blood biomarkers with the strongest overall CFE for tracking and predicting both depression and mania, hence bipolar mood disorders, after the first four steps were NRG1, DOCK10, GLS, PRPS1, TMEM161B, and SLC6A4 (Table 3B). For example, NRG1 (neuregulin 1) decreased in expression in high mood, survived discovery, prioritization and validation. It seems to be a better predictor for low mood/depression, especially personalized by gender and diagnosis, than for high mood/mania (Table 3).

### Targeted therapeutics

#### Pharmacogenomics

A number of individual top biomarkers are known to be modulated by medications in current clinical use for treating depression, such as by lithium (NRG1, PRPS1, CD47), antidepressants (SLC6A4, DOCK10, NRG1, CD47) and the nutraceutical omega-3 fatty acids (GLO1, SLC6A4, CD47, GLS, HNRNPDL) (Fig. S4 and Table 3 and S4). In particular, NRG1 is at the overlap of lithium and antidepressants, and CD47, which is involved in cell survival, is at the overlap of all three treatments (Fig. S4). This is of potential utility in patient stratification and pharmacogenomics approaches. Omega-3 fatty acids may be a widely deployable preventive treatment, with minimal side-effects, including in women who are or may become pregnant.

#### New drug discovery/repurposing

Bioinformatic analyses using the gene expression signature of panels of top biomarkers for low mood/depression (Table 4) identified new potential therapeutics for depression, such as the beta-blocker and serotonin 5HT1A presynaptic receptor antagonist pindolol, the PPAR-alpha activator and lipid lowering agent ciprofibrate, the PPAR-γ activator and anti-diabetic pioglitazone, and the anticholinergic and antispasmodic adiphenine. It also identified the natural compounds asiaticoside and chlorogenic acid. The last three had also been identified by our previous suicidality studies. Asiaticoside is a triterpenoid component derived from Centella asiatica (Gotu Kola), used in antioxidant, anti-inflammatory, immunomodulatory, and wound healing applications. Chlorogenic acid is an antioxidant polyphenol found in coffee.

## Discussion

We describe a novel and comprehensive effort to discover and validate blood biomarkers of relevance to mood disorders, including testing them in independent cohorts to evaluate predictive ability and clinical utility. These biomarkers also open a window into understanding the biology of mood disorders in general, and of depression and bipolar disorders in particular, as well as indicate new and more precise therapeutic approaches. We provide support for the view that, while mood is a continuum from low to high mood, with some of the best predictive biomarkers for low mood/depression and high mood/mania being shared (with changes in opposite direction), some biomarkers are stronger predictors for clinical depression and others for clinical mania, not surprising given the different co-morbidities associated with those conditions.

### Current clinical practice and the need for biomarkers

A convergence of methods assessing the persons’ internal subjective feelings and thoughts, along with more objective external ratings of actions and behaviors, is used de facto in clinical practice to assess mood and diagnose clinical mood disorders, such as depression and bipolar disorders. Such an approach is insufficient, and lagging behind those used in other medical specialties. Moreover, ~70% of patients with bipolar I disorder (BP-I) are initially misdiagnosed, with a mean delay of 5–10 years between illness onset and diagnosis. Most commonly patients are misdiagnosed with major depressive disorder (MDD) [27, 28]. Blood biomarkers related to mood would provide a critical objective measurement to inform clinical assessments and treatment decisions (Fig. 4).

### Brain–blood

Blood biomarkers offer real-world clinical practice advantages. As the brain cannot be readily biopsied in live individuals, and CSF is less easily accessible than blood, we have endeavored over the years to identify blood biomarkers for neuropsychiatric disorders. A whole-blood approach facilitates field deployment of sample collection. The assessment of gene expression changes focuses our approach on immune cells. The ability to identify peripheral gene expression changes that reflect brain activities is likely due to the fact that the brain and immune system have developmental commonalities, marked by shared reactivity and ensuing gene expression patterns. There is also a bi-directional interaction between the brain and immune system. Not all changes in expression in peripheral cells are reflective of or germane to brain activity. By carefully tracking a phenotype with our within-subject design in the discovery step, and then using CFG prioritization, we are able to extract the peripheral changes that do track and are relevant to the brain activity studied, in this case mood state, and its disorders.

Subsequent validation and testing in independent cohorts narrow the list to the best markers. In the end, we do not expect to recapitulate in the blood all that happens in the brain. We just want to have good accessible peripheral biomarkers—“liquid biopsies”, as they are called in cancer.

### Comprehensive approach

In this current work, we carried out extensive blood gene expression studies in male and female subjects with major psychiatric disorders, an enriched population in terms of co-morbidity with mood disorders and variability of mood. The potential molecular-level co-morbidity between other psychiatric disorders and mood disorders is underlined by the fact that mood medications (antidepressants, mood stabilizers) are used to treat PTSD and schizoaffective disorders, and antipsychotics are used to treat mood disorders. Our goal is first and foremost to discover and validate biomarkers for mood, that are transdiagnostic, in the spirit of RDoC. Second, we aim to understand their universality vs. their specificity by gender and psychiatric diagnosis.

Our studies were stacked in an innovative and comprehensive fashion. First, we endeavored to discover blood gene expression biomarkers for mood using a longitudinal design, looking at differential expression of genes in the blood of male and female subjects with major psychiatric disorders (bipolar disorder, MDD, schizophrenia/schizoaffective, and post-traumatic stress disorder (PTSD)), high risk populations prone to mood disorders, which constitute and enriched pool in which to look for biomarkers. We compared low-mood states to high-mood states using a powerful within-subject design [3–5, 29], to generate a list of differentially expressed genes. Second, we used a comprehensive CFG approach with the whole body of knowledge in the field to date to prioritize from the list of differentially expressed genes/biomarkers of relevance to mood. CFG integrates multiple independent lines of evidence—genetic, gene expression, and protein data, from brain and periphery, from human and animal model studies, as a Bayesian strategy for identifying and prioritizing findings, reducing the false-positives and false-negatives inherent in each individual approach. Third, we examined if the expression levels of the top biomarkers identified by us as tracking mood state is changed even more dramatically in blood samples from an independent cohort of subjects with severe depression and with severe mania, to validate these biomarkers. Fourth, the markers thus discovered, prioritized, and validated were tested in corresponding independent cohorts of psychiatric subjects. Fifth, we used the biomarkers to match to existing psychiatric medications, as well as to identify and potentially repurpose new drugs for mood disorders treatment using bioinformatics analyses. The series of studies was a systematic approach to move the field forward toward precision medicine.

### Power considerations

The current work is more comprehensive and powerful in design, and larger in size, than our previous studies [1]. We used a systematic discovery, prioritization, validation, and testing approach, as we have done more recently for suicide and other disorders [3–5, 8–10]. For discovery, we used a hard to accomplish but powerful within-subject design, with an *N* of 44 subjects with 134 visits. A within-subject design factors out genetic variability, as well as some medications, lifestyle, and demographic effects on gene expression, permitting identification of relevant signal with Ns as small as 1 [29]. Another benefit of a within-subject design may be accuracy/consistency of self-report of psychiatric symptoms (“phene expression”), similar in rationale to the signal detection benefits it provides in gene expression.

Based on our work for the last two decades in genetics and gene expression, along with the results of others in the field, we estimate that the within-subject longitudinal design, by factoring out all genetic and some environmental variability, is up to three orders of magnitude more powerful than an inter-subject case-control cross-sectional design. Moreover, gene expression, by integrating the effects of many SNPs and environment, is up to three orders of magnitude more powerful than a genetic study. Combined, our approach may be up to six orders of magnitude more powerful than a GWAS study, even prior to the CFG literature-based prioritization step. As such, it is at least comparable in power to the largest GWAS to date.

### Reproducibility

Besides our top biomarkers, deeper down in our datasets, we reproduced and expanded our earlier findings of GRK3 (Niculescu et al. Physiological Genomics 2000) [30] and FGFR1 (Le-Niculescu et al. Molecular Psychiatry 2009) [1] as blood biomarkers tracking and predicting mood.

In addition, there is reproducibility with findings generated by independent large scale genetic studies that came out after our analyses were completed, and were thus not included in our CFG approach. A number of their top findings were present in our candidate gene expression biomarkers for mood list that had survived our initial whole-genome, unbiased, within-subject discovery step, before any CFG literature prioritization: 15 out of their 36 top genes for bipolar disorder (Stahl et.al., their Table 1) [31], 187 out of 553 genes for depression (Coleman et.al, their Table S4) [32], 128 out of 268 genes for depression (Howard et al., their Table S9) [33], 487 out of 1291 genes for depression (Chan et al., their Tables S2, S3, S14, S17) [34], 491 out of 819 genes involved in antidepressant response [35], and 79 out of 223 genes for depression (Levey et al. 2020 Medrxiv.org, their Supplementary Tables 1 and 3) (see Supplementary Information- Pathways, Predictions and Reproducibility file). This independent reproducibility of findings between our studies and the genetic studies, which are done in independent cohorts from ours, with independent methodologies, is reassuring, and provides strong convergent evidence for the validity and relevance of our approach and of their genetic approaches. Our work also provides functional evidence for some of their top genetic hits.

### Pathophysiological insights

A number of top candidate biomarkers identified by us have biological roles that are related to the circadian clock (Table S3). To be able to ascertain all the genes in our dataset that were circadian and do estimates for enrichment, we compiled from the literature a database of all the known circadian genes, numbering a total of 1468 genes. Using an estimate of about 21,000 genes in the human genome, that gives about 7% of genes having some circadian pattern. Out of our 23 top candidate biomarker genes, eight had circadian evidence (35%), suggesting a fivefold enrichment for circadian genes. This provides a molecular underpinning for the epidemiological data between disrupted sleep and mood disorders, and for the clinical phenomenology of seasonal components to mood disorders.

The majority of top blood biomarkers we have identified have prior evidence in postmortem brain datasets from mood disorders, which indicates their relevance to the pathophysiology of mood disorders (Table S2). The co-directionality of blood changes in our work and brain changes reported in the literature needs to be interpreted with caution, as it may depend on brain region, and on disease stage.

The top biomarkers also had prior evidence of involvement in other psychiatric and related disorders (Table S3), providing a molecular basis for co-morbidity, and the possible precursor effects of some these disorders on mood, and conversely, the precursor role of mood in some of them. In particular, a majority of them have an overlap with suicide (92%), as well as stress (92%), aging (83%) and dementia/Alzheimer (75%), consistent with them being part of the effects of stress on aging and the “life switch”, as described in a previous study by us [26]. The primary medications used to treat stress disorders are serotonin reuptake inhibitors (SSRIs).

#### Phenomenology (phenomics)

The mood SMS-7 consists of seven items (Supplementary Fig. S1A). Our clustering analysis revealed the structure of mood symptoms (Supplementary Fig. S1C). Mood and Motivation to do things were the most closely related, followed by Movement activity and Thinking activity. Self-esteem and Interest in pleasurable activities are more distant, and related to each other. Appetite is the most distant, and least related to other items on the scale. Mood reflects and underlies, in essence, if an individual is motivated to get on with life/activities, or not, and if they are happy with themselves. Germane to that, we show that SMS-7 shows good correlation with items of a newly developed visual analog scale for Life Satisfaction (Happiness, Hope, Meaning) (Supplementary Fig. S1D).

### Diagnostics

For the biomarkers identified by us, combining all the available evidence from this current work into a CFE score, brings to the fore biomarkers that have clinical utility for objective assessment and risk prediction for depression, bipolar disorder, and mania (Table 3A–C). These biomarkers should be tested individually as well as tested as polygenic panels of biomarkers in future clinical studies and practical clinical applications in the field. They may permit to distinguish, upon an initial clinical presentation of depression, whether the person is in fact bipolar (Fig. 4). The integration of phenomic data, such as repeated measures of SMS-7 (perhaps via a phone app in a daily fashion), and our CFI-BP score, can further substantiate and elucidate the diagnoses of depression, bipolar disorder, and distinguishing between the two.

In general, our predictive results with biomarkers were stronger in females than in males, by an order of 10–20% on AUCs. While some of it may be biological, in terms of brain–immune  interplay being perhaps higher in women, it is also possible that men are not as accurate as women in terms of self-reporting mood symptoms (affecting our results on state predictions), and do not seek help as much (affecting our results on future hospitalizations predictions). If so, this under-reporting makes the use of objective biomarker tests in men even more necessary.

In regards to how our biomarker discoveries might be applied in clinical laboratory settings, we suggest that panels of top biomarkers, such as BioM12 + 1, be used (Fig. 4). In practice, every new patient tested would be normalized against the database of similar patients already tested, and compared to them for ranking and risk prediction purposes, regardless if a platform like microarrays, RNA sequencing, or a more targeted one like PCR is used in the end clinically. As databases get larger, normative population levels can and should be established, similar to any other laboratory measures. Moreover, longitudinal monitoring of changes in biomarkers within an individual, measuring most recent slope of change, maximum levels attained, and maximum slope of change attained, may be even more informative than simple cross-sectional comparisons of levels within an individual with normative populational levels, as we have shown in our studies. For future point of care approaches, research and development should focus on top individual biomarkers, including at a protein level in accessible fluids such as saliva. One might look at both the best universal biomarkers (that are predictive in all), for reliability, and at the best personalized biomarkers (that are predictive by gender and diagnosis), for higher accuracy.

### Treatment

Biomarkers may also be useful for matching patients to medications and measuring response to treatment (pharmacogenomics) (Fig. 4, Table 3 and S4), as well as new drug discovery and repurposing (Table 4).

## Conclusions

Overall, this work is a major step forward towards understanding, diagnosing, and treating mood disorders. We hope that our trait biomarkers for future risk may be useful in preventive approaches, before the full-blown disorder manifests itself (or re-occurs). Prevention could be accomplished with social, psychological, or biological interventions (i.e., early targeted use of medications or nutraceuticals). Given the fact that 1 in 4 people will have a clinical mood disorder episode in their lifetime, that mood disorders can severely affect quality of life, sometimes leading to suicides, and that not all patients respond to current treatments, the need for and importance of efforts such as ours cannot be overstated.

## Supplementary information

Supplementary Information - Figures S1-S4 and Tables S1- S4

Supplementary Information- Pathways, Predictions, and Reproducibility
