# Supplementary material for: Precision medicine for mood disorders: objective assessment, risk prediction, pharmacogenomics, and repurposed drugs
Source: Mol Psychiatry. 2021 Apr 8;26(7):2776–804. doi: 10.1038/s41380-021-01061-w (PMC8505261; doi:10.1038/s41380-021-01061-w)
Supplement: Supplementary file 1 — Supplementary Information - Figures S1-S4 and Tables S1- S4 [file 41380_2021_1061_MOESM1_ESM.docx]

**Supplementary Information**

**Figure S1:**

1. **Visual Analog Scale for Measuring Mood State.** Mood Subscale (SMS, Simplified Mood Scale), of the Simplified Affective State Scale (SASS) (Niculescu et al. 2006, 2015). Score is average of 7 items.

For each item, mark the scale with a vertical line where you think you are at this moment in time, compared to lowest and highest you ever remember being:

**1) Mood**

How good is your mood right now?

**[------------------------------------------------------------------------]**

**Lowest Highest**

**2) Motivation to do things**

How is your motivation, your drive, your determination to do things right now?

**[------------------------------------------------------------------------]**

**Lowest Highest**

**3) Movement activity**

How high is your physical energy and the amount of moving about that you feel like doing right now?

**[------------------------------------------------------------------------]**

**Lowest Highest**

**4) Thinking activity**

How high is your mental energy and thinking activity going on in your mind right now?

**[------------------------------------------------------------------------]**

**Lowest Highest**

**5) Self-esteem**

How good do you feel about yourself and your accomplishments right now?

**[------------------------------------------------------------------------]**

**Lowest Highest**

**6) Interest in pleasurable activities**

How high is your interest to do things that are fun and enjoyable right now?

**[------------------------------------------------------------------------]**

**Lowest Highest**

**7) Appetite**

How high is your appetite and desire for food right now?

**[------------------------------------------------------------------------]**

**Lowest Highest**

1. **Correlation between HAMD and SMS7 in the whole population used in this study (n=794 testing visits).**
2. **The Structure of Mood- PhenoChipping Clustering of items of the SMS-7 for measuring mood state.** Analyzing quantitative phenomic data as we would gene expression data. Two-way unsupervised hierarchical clustering using the Discovery cohort data (n=134 visits, from 44 subjects). Red- increased values/expression. Blue-decreased values/expression. Mood is most closely related to motivation to do things, followed by movement activity and thinking activity. Self-esteem and Interest in pleasurable activities are more distant, and related to each other. Appetite is the most distant, and least related to other items.


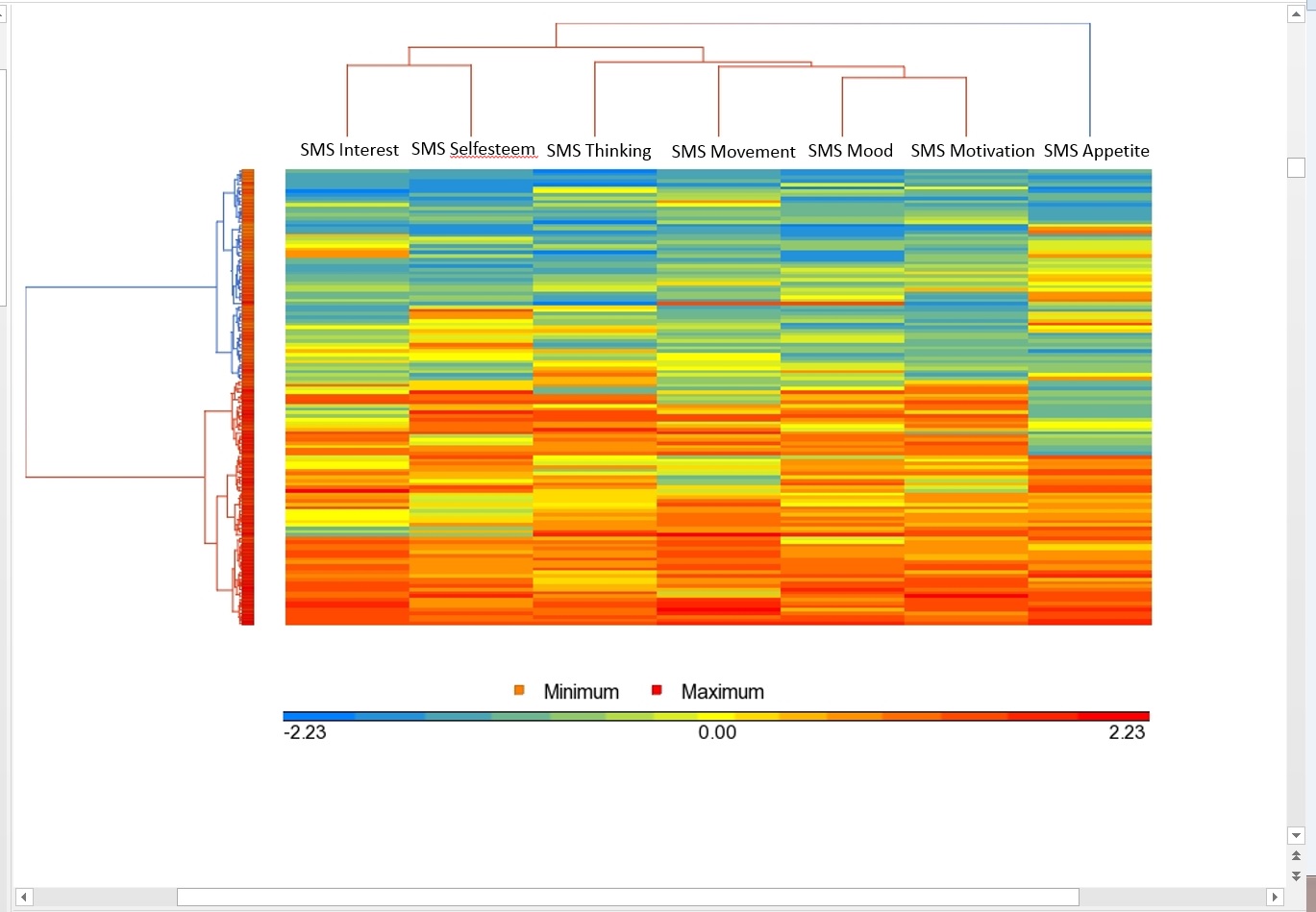


1. **Correlation between Happiness, Hope and Meaning with SMS7 in a subset of the population used in this study on which we had visual analog scales for Happiness, Hope and Meaning (n=84 testing visits).**

Life Satisfaction Scale

For each item, score where you think you are at this moment in time, compared to lowest and highest you ever remember it being:

**1)** How happy are you right now?

**Lowest [-----------------------------------------------------------------------] Highest**

**0 100**

**2)** How hopeful are you about the future right now?

**Lowest [-----------------------------------------------------------------------] Highest**

**0 100**

**3)** How full of meaning is your life right now?

**Lowest [-----------------------------------------------------------------------] Highest**

**0 100**

| Pearson Correlation  R2 (all p<0.001) | Happy | Hope | Meaning |
| --- | --- | --- | --- |
| SMS7 | 0.62 | 0.62 | 0.51 |

**E. Subtypes of low mood.** Low mood visits (n=63) in the 44 subjects in the Discovery cohort. Data Z-scored by gender and diagnosis. Blue- lower values, Red-higher values. X-axis are subject visits. Y-axis are anxiety and psychosis measures.

**
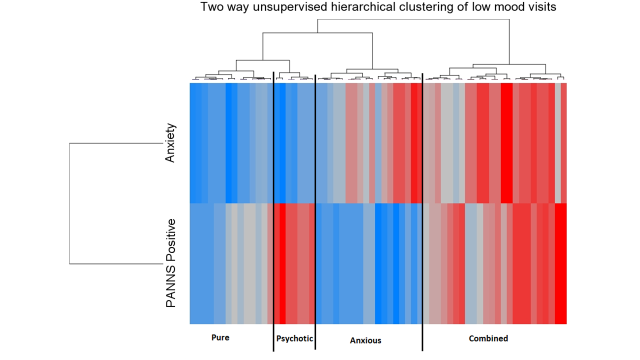
**

**Figure S2.**

**CFI-BP (Convergent Functional Information of Bipolar Disorder Severity)**

1-10 Score

Medications

M1. Tried on more than two different mood stabilizing medications (1 pt.)

M2. Was/is on lithium or divalproex (1 pt.)

M3. Was/is on antipsychotics (1 pt.)

Medication Score (0-3): ________________

Severity of illness

I1. Multiple hospitalizations on inpatient psychiatric units (1 pt.)

I2. Hospitalized in a State Hospital, or for more than 21 days (1 pt.)

I3. YMRS greater than I5 or HAM-D greater than 20 at time of testing (1 pt.)

I4. No history of enrollment in substance abuse programs or treatments (1 pt.)

Severity Score (0-4): ________________

Social functioning

F1. On 100% disability (1 pt.)

F2. Was/is on commitment (has conservator/payee) (1 pt.)

F3. Has been in bankruptcy or lost home to foreclosure or married more than 3 times (1 pt.)

Social Functioning Score (0-3): ________________

**Total Score (0-10): ________________**

**Figure S3:** [STRING Interaction Network](http://version10.5.string-db.org/newstring_cgi/show_network_section.pl?limit=0&targetmode=proteins&caller_identity=gene_cards&network_flavor=evidence&identifiers=9606.ENSP00000414303%0d%0a9606.ENSP00000397297%0d%0a9606.ENSP00000304669%0d%0a9606.ENSP00000339007%0d%0a9606.ENSP00000353483%0d%0a9606.ENSP00000260227%0d%0a9606.ENSP00000401303%0d%0a9606.ENSP00000340698%0d%0a9606.ENSP00000263967%0d%0a9606.ENSP00000265164%0d%0a9606.ENSP00000244007%0d%0a9606.ENSP00000269141%0d%0a9606.ENSP00000358525%0d%0a9606.ENSP00000274335%0d%0a9606.ENSP00000314458%0d%0a9606.ENSP00000178640%0d%0a9606.ENSP00000321209%0d%0a9606.ENSP00000250559%0d%0a9606.ENSP00000352157%0d%0a9606.ENSP00000264554%0d%0a9606.ENSP00000269305%0d%0a9606.ENSP00000348786%0d%0a9606.ENSP00000431418%0d%0a9606.ENSP00000356056%0d%0a9606.ENSP00000308938%0d%0a9606.ENSP00000364995%0d%0a) for nominally significant predictive biomarkers for low mood/depression/hospitalizations across all subjects in the independent test cohort (n= 23 genes, 26 probesets)

**
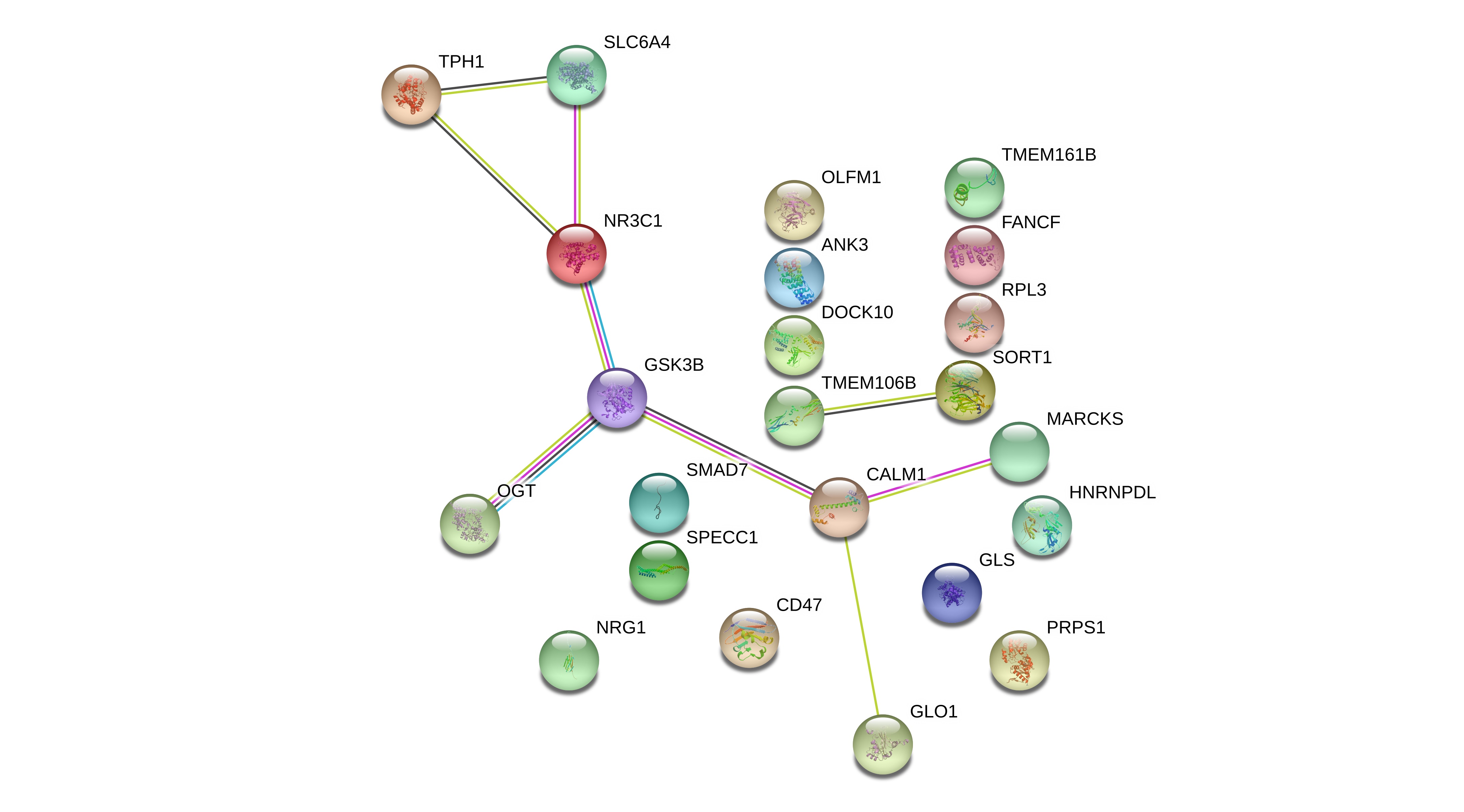
**

**Figure S4. Pharmacogenomics for Depression (BioM12).** Top biomarkers for depression that have evidence for being modulated by existing drugs in the opposite direction to depression/low mood. See also Table S4. Blue- increased in expression in low mood, red -increased in expression in high mood. Such biomarkers could be used to *target* treatments to different patients, and to measure *response* to that treatment. The higher the proportion/percentile of biomarkers for a certain drug/class, the more indicated that drug would be for treatment. When biomarkers for multiple different drug/classes are changed in an individual, a prioritization based on the proportion/percentile of biomarkers for each class could be used to choose the drug or combination of drugs (targeted rational polypharmacy). See Figure 4 for an example.

**
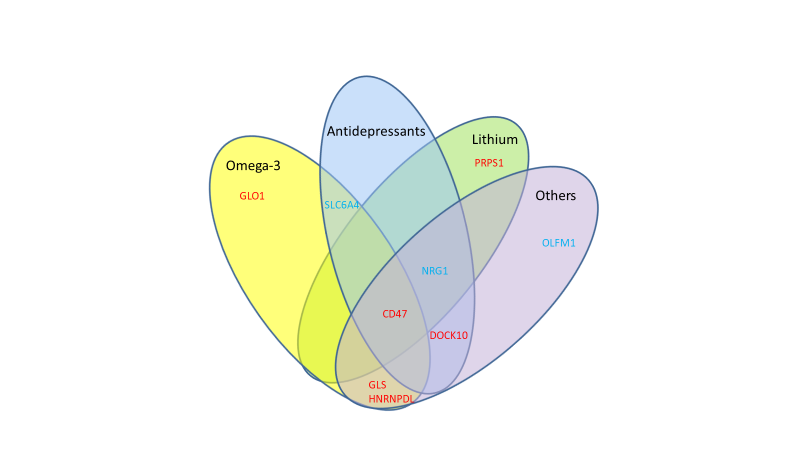
**

**Table S1 Combined Genomic and Phenomic Predictor (UP-Mood).** Predictions using an apriori algorithm combining as predictors BioM26 with mood (SMS7) and with clinical severity of bipolar disorder (CFI-BP). In All subjects in the independent test cohort. Cross-sectional analyses.

| **Universal Predictor Mood (UP-Mood)**  **(BioM26 + CFI-BP+ SMS7)** | Depression | Mania |
| --- | --- | --- |
| State  Clinically Severe | **AUC 69.1%**  **P=4.79E-04** | **AUC 71.5%**  **P=5E-02** |
| Trait  All Future Hospitalizations | **OR 1.2**  **P=1.57E-02** | **OR 1.6**  **P= 1.4E-03** |

**Table S2. CFG for Mood- used in Step 2 Prioritization for Top Biomarkers for Low Mood/ Depression (BioM12 Depression) and Mania (RLP3).** Red- increased in expression (I) in High Mood, Blue- decreased in expression in High Mood (D). DE- differential expression, AP-Absent/Present.

| **Gene Symbol/ Gene Name** | **Probesets** | **(Direction of Change) Method/ Score/ %** | **Prior Human Genetic Evidence** | **Prior Human Brain Tissue Evidence** | **Prior Human Peripheral Evidence** | **Prior Non-human Genetic Evidence** | **Prior Non-human Brain Tissue Evidence** | **Prior Non-human Peripheral Evidence** | **Step 2**  **Prioritization CFG Score For Mood** | **Step 1-4 CFE** |
| --- | --- | --- | --- | --- | --- | --- | --- | --- | --- | --- |
| **NRG1** neuregulin 1 | 208230_s_at | (D) DE/2 33.7% | **MDD** [^1^](#_ENREF_1)       **BP** [^2^](#_ENREF_2), [^3^](#_ENREF_3),[^4^](#_ENREF_4),[^5^](#_ENREF_5),  [^6^](#_ENREF_6), [^7^](#_ENREF_7) | (D)  CA3/2 Stratum oriens **BP** [^8^](#_ENREF_8)  (D) Hippocampus  **BP** [^8^](#_ENREF_8), [^9^](#_ENREF_9)  (D) prefrontal cortices **MDD** [^10^](#_ENREF_10)  (D) BA8/9 Female **MDD** [^11^](#_ENREF_11)  (D) Hippocampus **BP** [^9^](#_ENREF_9) | (I) PBMC MDD [^12^](#_ENREF_12)  (D) PBMC **Antidepressants** [^12^](#_ENREF_12)  (I) peripheral blood mononuclear cells BP [^13^](#_ENREF_13) |  | (I)  AMY **MDD** [^14^](#_ENREF_14), [^15^](#_ENREF_15) |  | 10 | 26 |
| **DOCK10** dedicator of cytokinesis 10 | 219279_at | (I) DE/2 41.5% | **BP**  [^16^](#_ENREF_16) Linkage | (I)  Ventral Subiculum Female **MDD** [^11^](#_ENREF_11) | (I)  Blood  **BP** [^17^](#_ENREF_17) |  | (D)  PFC  **MDD** [^18^](#_ENREF_18) |  | 10 | 24 |
| **GLS** Glutaminase | 203159_at | (I) DE/4 53.7% |  | (I) BA46 **MDD** [^19^](#_ENREF_19)  (D) Brain **BP** [^20^](#_ENREF_20)  FPC differentially expressed genes MDD [^21^](#_ENREF_21)    (D) FTPFC MDD[^21^](#_ENREF_21)  (I)Anterior PFC BP [^22^](#_ENREF_22) | (D) Lymphoblastoid Cell Lines  **BP, MDD**  [^23^](#_ENREF_23) |  | (D) Hippocampus **MDD**[^15^](#_ENREF_15)  (I) AMY (males) **BP** [^24^](#_ENREF_24) |  | 8 | 24 |
| **PRPS1** Phosphoribosyl Pyrophosphate Synthetase 1 | 209440_at | (I) DE/4 57.3% | Linkage | (D) AMY **MDD** [^25^](#_ENREF_25)    (D) BA 10  **MDD** [^26^](#_ENREF_26)  Brain  (D) DLPFC **BP** [^20^](#_ENREF_20)  (D) **MDD** [^27^](#_ENREF_27)  (D) PFC **MDD**[^26^](#_ENREF_26)    (I) PFC **MDD** [^25^](#_ENREF_25) | Whole blood DNA Differentially methylated  **BP**  [^28^](#_ENREF_28) |  | (I) PFC **MDD** [^26^](#_ENREF_26) |  | 9 | 24 |
| **TMEM161B** Transmembrane Protein 161B | 227861_at | (I) AP/4 62.1% | **Depression** [^29^](#_ENREF_29)  **Depression** [^30^](#_ENREF_30) | (D) BA8/9 Male **MDD** [^11^](#_ENREF_11) | (I) L neurons **BP** [^31^](#_ENREF_31) |  | (I) AMY **MDD** [^15^](#_ENREF_15) |  | 10 | 24 |
| **GLO1** Glyoxalase I | 200681_at | (I) DE/2 41.5% | MDD [^1^](#_ENREF_1) | (D) Brain **BP** [^20^](#_ENREF_20)  (D) Hippocampus **BP** [^32^](#_ENREF_32)  (I) BA11, Subic Female **MDD** [^11^](#_ENREF_11)  (I) Anterior PFC **BP ,MDD** [^22^](#_ENREF_22) | (D) Peripheral white blood cells **BP** [^33^](#_ENREF_33) | **Depression-related**[^34^](#_ENREF_34) | (D) Hippocampus, mPFC **Antidepressants** [^34^](#_ENREF_34)  (D) Cortex **Fluoxetine** [^35^](#_ENREF_35) | (D) Hippocampus, mPFC **Antidepressants** [^34^](#_ENREF_34) | 11.50 | 22.5 |
| **FANCF** Fanconi Anemia Complementation Group F | 218689_at | (I) DE/4 54.9% |  | (D) Brain **BP** [^20^](#_ENREF_20)  (I) BA11, BA25, Subic Female **MDD** [^11^](#_ENREF_11) | (D) Blood **MDD** [^25^](#_ENREF_25) |  | (D) AMY,PFC (males) **BP**  [^24^](#_ENREF_24) |  | 8 | 22 |
| **HNRNPDL** Heterogeneous Nuclear Ribonucleoprotein D Like | 212454_x_at | (I) DE/2 35.4% |  | (D) AMY and cingulate cortex **MDD** [^36^](#_ENREF_36)    (I) NAC Male **MDD** [^11^](#_ENREF_11) | (I) Fibroblast **MDD** [^37^](#_ENREF_37) |  | (D) Cerebral Cortex (right) **Lithium**  [^38^](#_ENREF_38)    (I) AMY (males) **BP** [^24^](#_ENREF_24) | (D) Lymphocytes (males) **BP** [^24^](#_ENREF_24) | 10 | 22 |
| **NRG1** Neuregulin 1 | 208232_x_at | (D) AP/4 60.7% | **MDD** [^1^](#_ENREF_1)       **BP** [^2^](#_ENREF_2), [^3^](#_ENREF_3),[^4^](#_ENREF_4),[^5^](#_ENREF_5),  [^6^](#_ENREF_6), [^39^](#_ENREF_39) | (D)  CA3/2 Stratum oriens **BP** [^8^](#_ENREF_8)  (D) Hippocampus  **BP** [^8^](#_ENREF_8), [^9^](#_ENREF_9)  (D) prefrontal cortices **MDD** [^10^](#_ENREF_10)  (D) BA8/9 Female **MDD** [^11^](#_ENREF_11)  (D) Hippocampus **BP** [^9^](#_ENREF_9) | (I) PBMC MDD [^12^](#_ENREF_12)  (D) PBMC **Antidepressants** [^12^](#_ENREF_12)  (I) peripheral blood mononuclear cells BP [^13^](#_ENREF_13)  (D) peripheral blood mononuclear cells **MDD** [^12^](#_ENREF_12) |  | (I)  AMY **MDD** [^14^](#_ENREF_14), [^15^](#_ENREF_15) |  | 10 | 22 |
| **CD47** CD47 Molecule | 213856_at | (I) AP/4 66.7% |  | (I) AMY and cingulate cortex **MDD** [^36^](#_ENREF_36) | (D) Peripheral venous blood **MDD**  [^40^](#_ENREF_40) |  | (D) Hippocampus **MDD**  [^41^](#_ENREF_41) |  | 8 | 21 |
| **OLFM1** Olfactomedin 1 | 210924_at | (D) DE/2 33.7% | BP [^42^](#_ENREF_42) | (D) Brain **BP** [^20^](#_ENREF_20)  (D) Cerebral cortex **BP**  [^43^](#_ENREF_43) | (D) NT2.D1 cells **Valproate** [^44^](#_ENREF_44) |  | (D) NAC **MDD**  [^18^](#_ENREF_18) | (D) Blood **MDD**  [^45^](#_ENREF_45) | 10.00 | 21 |
| **SMAD7** SMAD Family Member 7 | 204790_at | (I) DE/2 42.7% (I) AP/4 54% |  | (I) DLPFC (BA46) **BP** [^46^](#_ENREF_46)  (D) Anterior Insula Male **MDD**  [^11^](#_ENREF_11) | (D) Blood **MDD** [^47^](#_ENREF_47)  (I) Blood **Antidepressants**  [^48^](#_ENREF_48) |  | (D) Dentate Gyrus **Antidepressants, Fluoxetine** [^14^](#_ENREF_14) |  | 9 | 21 |
| **SLC6A4** solute carrier family 6 (neurotransmitter transporter), member 4 | 242009_at | (D) DE/4 64.1% | **Affective Disorder** [^49^](#_ENREF_49),[^50^](#_ENREF_50)  **BP**  [^51^](#_ENREF_51), [^52^](#_ENREF_52),[^53^](#_ENREF_53),[^54^](#_ENREF_54),[^55^](#_ENREF_55)  **Depression** [^56^](#_ENREF_56)  **MDD**  [^57^](#_ENREF_57),[^58^](#_ENREF_58),  [^59^](#_ENREF_59),[^60^](#_ENREF_60),[^61^](#_ENREF_61),[^62^](#_ENREF_62),[^63^](#_ENREF_63)  **Mood Disorders NOS**  [^64^](#_ENREF_64)  **Lithium** [^65^](#_ENREF_65)  Linkage | (D)  **BP** [^66^](#_ENREF_66)    (D) PFC  **MDD** [^67^](#_ENREF_67)  (I)  Ventral Subiculum Female **MDD** [^11^](#_ENREF_11)  (D) midbrain, caudate **BP** [^68^](#_ENREF_68)  (D)  Thalamus **MDD** [^57^](#_ENREF_57) | (D) **MDD** [^69^](#_ENREF_69)  (I) PBMC  **Antidepressants** [^12^](#_ENREF_12)  (D)  Neural progenitor cells (NPCs) **Antidepressants** [^70^](#_ENREF_70)  (I) placenta **MDD** [^71^](#_ENREF_71)  (I) PBMC cells **MDD** [^12^](#_ENREF_12)  (I) Blood **MDD**[^72^](#_ENREF_72) |  | (I)  PFC  **MDD** [^73^](#_ENREF_73)  (I)  Embryonic hippocampal ,PFC neurons  **MDD** [^74^](#_ENREF_74)  (I) Cortex **Fluoxetine** [^35^](#_ENREF_35) |  | 10 | 20 |
| Biomarker for Mania | | | | | | | | | | |
| **RPL3** Ribosomal Protein L3 | 212039_x_at | (I) DE/4 50% |  | (I)  BA11, (D) NAC Female **MDD** [^11^](#_ENREF_11)  (I)  BA25, BA8/9 Male **MDD** [^11^](#_ENREF_11) | (I)  Blood  **MDD**  [^75^](#_ENREF_75) |  | (D) Ventral Hippocampus **MDD**  27181059  (I) PFC **MDD, Ventral medial hippocampus** [^18^](#_ENREF_18) |  | 8 | 21 |

**Table S3. Evidence for Involvement in Other Disorders for Top Biomarkers for Low Mood/ Depression (BioM12 Depression).** Red- increased in expression (I) in High Mood, Blue- decreased in expression in High Mood (D).

| **Gene Symbol/ Gene Name** | **Probesets** | **(Direction of Change) Method/ Score/ %** | **Prior Human Genetic Evidence** | **Prior Human Brain Tissue Evidence** | **Prior Human Peripheral Evidence** | **Prior Non-human Genetic Evidence** | **Prior Non-human Brain Tissue Evidence** | **Prior Non-human Peripheral Evidence** | **CFG Score For**  **Other Disorders** | **Step 1-4**  **CFE** |
| --- | --- | --- | --- | --- | --- | --- | --- | --- | --- | --- |
| **DOCK10** dedicator of cytokinesis 10 | 219279_at | (I) DE/2 41.5% | **Aging** [^76^](#_ENREF_76) | (D) Cerebellum **Aging**  [^77^](#_ENREF_77)  (D) Superior frontal cortex **Alcohol** [^78^](#_ENREF_78)  (D) Parietal Lobe **Dementia** [^79^](#_ENREF_79)  (D) OFC **PTSD** [^80^](#_ENREF_80) | (D) Lymphocyte**SZ** [^81^](#_ENREF_81)  (D) Blood **Aging** [^77^](#_ENREF_77)  (D) Blood **Female Suicide** [^82^](#_ENREF_82)  (D) Blood **Suicide** [^83^](#_ENREF_83)  (D) Blood **Stress** [^84^](#_ENREF_84) |  | (D) Nac **Social Defeat** [^85^](#_ENREF_85)  (D) Female Nac **Stress** [^11^](#_ENREF_11) |  | 10 | 24 |
| **GLS** Glutaminase | 203159_at | (I) DE/4 53.7% | **Aging** [^86^](#_ENREF_86)  **SZ** [^87^](#_ENREF_87)  **SZ** [^43^](#_ENREF_43)  **Suicide** [^88^](#_ENREF_88) | (D) Cerebral cortex **ASD**,**SZ** [^43^](#_ENREF_43)  (D) Temporal Cortex **Dementia** [^89^](#_ENREF_89)  (D) Hippocampus **Dementia** [^90^](#_ENREF_90)  (D) BA46 **Suicide** [^19^](#_ENREF_19)  (D) Frontopolar cortex **Suicide** [^21^](#_ENREF_21)  (D) AMY **SZ**  [^91^](#_ENREF_91)  (D) DLPFC **SZ** [^23^](#_ENREF_23)  (D) Amygdala **SZ**  [^91^](#_ENREF_91)  (D) Prefrontal Cortex **SZ** [^92^](#_ENREF_92)  (D) DLPFC, Hippocampus, Associative striatum **SZ** [^93^](#_ENREF_93) | (D) Blood **Female Suicide** [^82^](#_ENREF_82)  (D) Blood **Suicide** [^83^](#_ENREF_83)  (D) Blood **Pain** [^94^](#_ENREF_94)  (D) PBMC **PTSD** [^95^](#_ENREF_95)  (D) Blood **Memory retention** [^96^](#_ENREF_96) |  | (D) CP (paradigm 2) **Alcohol** [^97^](#_ENREF_97)  (D) Hippocampus **Anxiety** [^98^](#_ENREF_98)  (D) MPFC **PTSD** [^99^](#_ENREF_99) |  | 8 | 24 |
| **PRPS1** Phosphoribosyl Pyrophosphate Synthetase 1 | 209440_at | (I) DE/4 57.3% |  | (D) Forebrain neural progenitor cells **SZ**  [^100^](#_ENREF_100)  (D) Frontal and temporal cortex, Cerebral cortex **Autism, SZ** [^43^](#_ENREF_43)^,^[^101^](#_ENREF_101)  (D) Hippocampus **Alzheimer's Disease** [^90^](#_ENREF_90)  (D) Hippocampus, Associative striatum , **SZ** [^93^](#_ENREF_93) | (D) PBMCs **ER Trauma survivors** [^95^](#_ENREF_95)  (D)  Blood **Aging** [^77^](#_ENREF_77)  (D) Blood **High Stress State** [^84^](#_ENREF_84)  (D) Blood **Female Suicide** [^82^](#_ENREF_82)    (D) Blood **Male Suicide** [^102^](#_ENREF_102)  (D) Blood **Suicide**[^83^](#_ENREF_83) |  |  |  | 9 | 24 |
| **TMEM161B** Transmembrane Protein 161B | 227861_at | (I) AP/4 62.1% | **Alcohol** [^103^](#_ENREF_103)  **Sleep** [^104^](#_ENREF_104)  **ASD, Neurological** [^105^](#_ENREF_105) |  | (D) Blood **Female Suicide** [^82^](#_ENREF_82)  (D) Blood **Suicide** [^83^](#_ENREF_83)  (D) Blood **Stress** [^84^](#_ENREF_84)  (D) Blood **Memory retention** [^96^](#_ENREF_96) |  | (D) PFC **Stress** [^106^](#_ENREF_106)  (I) NAC, PFC **Female Stress** [^11^](#_ENREF_11) |  | 10 | 24 |
| **GLO1** Glyoxalase I | 200681_at | (I) DE/2 41.5% | **Anxiety** [^107^](#_ENREF_107)  **Sleep** [^104^](#_ENREF_104)  **Panic** [^108^](#_ENREF_108) | (D) Frontal cortex **ASD** [^109^](#_ENREF_109)  (D) Hippocampus **Dementia** [^90^](#_ENREF_90)  (D) Parietal Lobe **Dementia** [^79^](#_ENREF_79)  (D) Hippocampus **SZ**  [^93^](#_ENREF_93)  (D) Dorsolateral prefrontal cortex **SZ** [^110^](#_ENREF_110)  (I) Anterior PFC **SZ ,SZA** [^22^](#_ENREF_22)  (D) Hippocampus **Alzheimer's Disease** [^111^](#_ENREF_111) | (D) Blood **Suicide** [^83^](#_ENREF_83)  (D) Lymphoblastoid cell lines (LCLs) **SZ** [^112^](#_ENREF_112)  (D) plasma **Aging** [^113^](#_ENREF_113) |  | (D) Hypothalamic PVN **Anxiety** [^114^](#_ENREF_114)  (D) AMY,CINGULATE CORTEX,HIPPOCAMPUS, ETC. **Anxiety** [^115^](#_ENREF_115)  (D) Cortex **Dementia** [^89^](#_ENREF_89)  (D) Hippocampus **Depression Susceptible** [^116^](#_ENREF_116) |  | 11.5 | 22.5 |
| **FANCF** Fanconi Anemia Complementation Group F | 218689_at | (I) DE/4 54.9% |  |  | (D) Blood **Stress** [^84^](#_ENREF_84) |  | (D) AMY (males) **Stress** [^24^](#_ENREF_24) |  | 8 | 22 |
| **HNRNPDL** Heterogeneous Nuclear Ribonucleoprotein D Like | 212454_x_at | (I) DE/2 35.4% |  | (D) Cerebral cortex **ASD** [^43^](#_ENREF_43)  (D) Hippocampus **Dementia** [^90^](#_ENREF_90)  (D) Parietal Lobe **Dementia** [^79^](#_ENREF_79)  (I) PFC **Suicide** [^117^](#_ENREF_117) | (D) PBMCs  **Aging** [^86^](#_ENREF_86)  (D) Blood **Aging** [^77^](#_ENREF_77)  (D) Blood **Female Suicide** [^82^](#_ENREF_82)  (D) Blood **Male Suicide** [^102^](#_ENREF_102)  (D) Blood **Suicide** [^83^](#_ENREF_83)  (D) Blood **PTSD** [^118^](#_ENREF_118)  (D) Blood **Stress** [^84^](#_ENREF_84)  (Differentially methylated)Whole blood DNA **SZ** [^28^](#_ENREF_28) |  | (D) AMY **Anxiety**  [^119^](#_ENREF_119)  (D) VT **Hallucinogens**  [^120^](#_ENREF_120) (D) PFC **Stress** [^121^](#_ENREF_121) | (D) Lymphocytes **Hallucinogens**  [^120^](#_ENREF_120)  (D) Spinal cord and DRG-RNA **Pain** [^122^](#_ENREF_122)  (D) Blood **Stimulants** [^123^](#_ENREF_123)  (I) Lymphocytes**Hallucinogens**  [^120^](#_ENREF_120) | 10 | 22 |
| **NRG1** Neuregulin 1 | 208232_x_at  208230_s_at | (D) AP/4 60.7%  (D) DE/2 33.7% | **Stimulants**  [^124^](#_ENREF_124)  **Aging** [^125^](#_ENREF_125)  **Anxiety** [^126^](#_ENREF_126)  **Psychosis** [^127^](#_ENREF_127)  **SZ** [^128^](#_ENREF_128) [^129^](#_ENREF_129)^,^[^130^](#_ENREF_130) [^2^](#_ENREF_2)^,^[^3^](#_ENREF_3)^,^[^131-138^](#_ENREF_131) [^139^](#_ENREF_139)^,^[^140^](#_ENREF_140) [^6^](#_ENREF_6)^,^[^141-145^](#_ENREF_141) [^146^](#_ENREF_146)  **Suicide** [^147^](#_ENREF_147) | (I) BA-9 PFC **SZ**  [^9^](#_ENREF_9)  (I) CA3/2 Stratum oriens **SZ** [^8^](#_ENREF_8)  (I) DLPFC **SZ** [^148^](#_ENREF_148)  (I) Hippocampus **SZ** [^149^](#_ENREF_149)  (I) PFC **SZ**  [^150^](#_ENREF_150)  (I) PFC **SZ**  [^151^](#_ENREF_151)  (I) PFC (BA-9) **SZ**  [^152^](#_ENREF_152)  (I) Brain **SZ** [^153^](#_ENREF_153)  (I) BA 9, Hippocampus **SZ**  [^9^](#_ENREF_9) | (Differential methylation) Blood **PTSD**  (I) **Aging** [^77^](#_ENREF_77)  (I) Blood **Suicide** [^83^](#_ENREF_83)  (I) Blood **Pain** [^94^](#_ENREF_94)  (I) Blood **Psychosis** [^154^](#_ENREF_154)  (I) glt8d1SH-SY5Y cells **Stimulants**[^155^](#_ENREF_155)  (I) Blood **Stress**  [^84^](#_ENREF_84)  (I) Peripheral blood monocytes **Stress** [^156^](#_ENREF_156)  (I) Leukocytes **SZ** [^141^](#_ENREF_141)  (I) Blood **SZ** [^157^](#_ENREF_157)  (I) Fibroblast **SZ** [^158^](#_ENREF_158)  (I) Lymphocyte **SZ** [^159^](#_ENREF_159) | **SZ**  [^160^](#_ENREF_160) [^161-164^](#_ENREF_161) | (I) mPFC, PL Cortex **Behavior** [^165^](#_ENREF_165)  (I) Prefrontal cortical tissue **Memory, SZ** [^166^](#_ENREF_166)  (I) AMY **Stress** [^167^](#_ENREF_167)  (I) Prefrontal cortex **SZ** [^168^](#_ENREF_168)  (I) Medial Prefrontal Cortex **SZ** [^162^](#_ENREF_162) | (I) Plasma **Aging** [^113^](#_ENREF_113) | 10 | 22  26 |
| **CD47** CD47 Molecule | 213856_at | (I) AP/4 66.7% | **Sleep** [^104^](#_ENREF_104) | (D) Entorhinal cortex **Dementia** [^169^](#_ENREF_169) | (D) Blood **Aging** [^77^](#_ENREF_77)  (D) Blood **Female Suicide** [^82^](#_ENREF_82)  (D) Blood **Male Suicide** [^102^](#_ENREF_102)  (D) Blood **Suicide** [^83^](#_ENREF_83)  (D) Blood **Pain** [^170^](#_ENREF_170)  (D) Peripheral blood monocytes **Stress** [^156^](#_ENREF_156)  (D) Neuroblastoma **SZ** [^171^](#_ENREF_171)  (D) Blood **SZ** [^172^](#_ENREF_172)  (D) Blood **Memory retention** [^96^](#_ENREF_96) |  |  |  | 8.00 | 21 |
| **OLFM1** Olfactomedin 1 | 210924_at | (D) DE/2 33.7% |  | (I) Brain, Orbitofrontal cortex **Suicide** [^173^](#_ENREF_173)  (I) Brain **Suicide** [^173^](#_ENREF_173)  (I) dlPFC (left hemisphere, Broadman area 46) **SZ** [^174^](#_ENREF_174)  (I) Frontal Lobe **Alcohol** [^175^](#_ENREF_175) | (I) Blood **PTSD** [^176^](#_ENREF_176)  (I) Blood **Aging** [^77^](#_ENREF_77)  (I) Blood **Memory retention** [^96^](#_ENREF_96) | **Alcohol**  [^177^](#_ENREF_177) | (I) NAC **Hallucinogens**  [^120^](#_ENREF_120)  (I) Cerebral Cortex **SZ**  [^178^](#_ENREF_178)  (I) PFC **SZ** [^179^](#_ENREF_179)  (I) Female NAC **Stress** [^11^](#_ENREF_11) |  | 10 | 21 |
| **SMAD7** SMAD Family Member 7 | 204790_at | (I) DE/2 42.7% (I) AP/4 54% | **Aging** [^125^](#_ENREF_125)  **SZ** [^43^](#_ENREF_43) | (D) Cerebellum **Aging** [^77^](#_ENREF_77)  (D) Parietal Cortex **Dementia** [^180^](#_ENREF_180)  (D) dlPFC **SZ** 31073119 | (I) Blood **Aging** [^77^](#_ENREF_77)  (D) Blood **Female Suicide** [^82^](#_ENREF_82)  (D) Blood **Stress** [^84^](#_ENREF_84) |  | (D) AMY **Stress**  [^181^](#_ENREF_181)  (D) Male Nac **Stress** [^11^](#_ENREF_11) |  | 9 | 21 |
| **SLC6A4** solute carrier family 6 (neurotransmitter transporter), member 4 | 242009_at | (D) DE/4 64.1% | **Alcohol** [^182-184^](#_ENREF_182)  [^185^](#_ENREF_185)  **Aging** [^186-188^](#_ENREF_186)  **Anxiety** [^189^](#_ENREF_189)^,^[^190^](#_ENREF_190)  [^191^](#_ENREF_191)^,^[^192^](#_ENREF_192)  **OCD**  [^193^](#_ENREF_193)^,^[^194^](#_ENREF_194)  [^195-197^](#_ENREF_195)  [^198^](#_ENREF_198)  [^199^](#_ENREF_199)  **ASD**  [^200^](#_ENREF_200)  [^43^](#_ENREF_43)^,^[^201^](#_ENREF_201)^,^[^202^](#_ENREF_202)  [^203^](#_ENREF_203)^,^[^204^](#_ENREF_204)  [^205^](#_ENREF_205)  [^206^](#_ENREF_206)^,^[^207^](#_ENREF_207)  **Behavior**[^208^](#_ENREF_208)  **Personality** [^209^](#_ENREF_209)^,^[^210^](#_ENREF_210)  **Pain** [^211^](#_ENREF_211)  [^212^](#_ENREF_212)  [^213-216^](#_ENREF_213)  **Panic** [^217-220^](#_ENREF_217)  **Stress** [^61^](#_ENREF_61)  [^221^](#_ENREF_221)  **PTSD** [^222^](#_ENREF_222)  **Suicide** [^59^](#_ENREF_59) [^209^](#_ENREF_209)^,^[^223-241^](#_ENREF_223) | (I) Hippocampus **Suicide** [^242^](#_ENREF_242) | (Hypermethylated) Blood **Stress** [^243^](#_ENREF_243)  (I) Blood **Stress, Anxiety** [^244^](#_ENREF_244)  (I) Blood **Stress** [^84^](#_ENREF_84)  (I) Blood **Pain** [^94^](#_ENREF_94)  (I) Lymphoblastoid **OCD**  [^245^](#_ENREF_245)  (I) Blood **Suicide** [^83^](#_ENREF_83)  (I) Blood **Female Suicide** [^82^](#_ENREF_82)  (I) Blood **Alcohol** [^246^](#_ENREF_246)  (I) PBMCs **Aging** [^86^](#_ENREF_86)  (Hypermethylation) Blood **Early Life Stress** [^243^](#_ENREF_243)    (D) CSF **Suicide** [^247^](#_ENREF_247)  PBMCs  **Aging** [^86^](#_ENREF_86) | **Alcoho**l [^177^](#_ENREF_177)  **ASD**  [^248^](#_ENREF_248) | (I) Hippocampus **Alcohol** [^249^](#_ENREF_249)  (I) Hippocampus **Anxiety** [^250^](#_ENREF_250) | (I) Lymphocytes**Hallucinogens**  [^120^](#_ENREF_120) | 10 | 20 |

**Table S4. Pharmacogenomics.Top Biomarkers for Low Mood/ Depression (BioM12 Depression).** Top biomarkers in our datasets that are targets of existing drugs and are modulated by them in **opposite direction to depression/same direction as high mood**. (I)- increased in expression, (D)- decreased in expression.

| **Gene Symbol/ Gene Name** | **Probesets** | **Discovery in Blood  (Direction of Change in High Mood) Method/ Score/ %  6pt** | **Omega-3** | **Antidepressants** | **Lithium and Other Mood Stabilizers** | **Anti-**  **psychotics** | **Others** | **Step 1-4 CFE Score** |
| --- | --- | --- | --- | --- | --- | --- | --- | --- |
| **NRG1** neuregulin 1 | 208230_s_at | (D) DE/2 33.7% |  | (D) PBMC **Antidepressants** [^12^](#_ENREF_12)  **Antidepressants** [^251^](#_ENREF_251) | (NA)  SH-SY5Y neuroblastoma cells  **Lithium**  [^252^](#_ENREF_252)  (D) Neuroblastoma cells **Valproate** [^253^](#_ENREF_253) | (D)blood  **Antipsychotic with weight gain in men**  [^254^](#_ENREF_254) |  | 26 |
| **DOCK10** dedicator of cytokinesis 10 | 219279_at | (I) DE/2 41.5% |  | (I)NAC  **Ketamine**[^85^](#_ENREF_85) |  |  | (I) HIP **Physical and Cognitive stimulation** [^255^](#_ENREF_255) | 24 |
| **GLS** glutaminase | 203159_at | (I) DE/4 53.7% | (I) HIP (males) **Omega-3 fatty acids** [^256^](#_ENREF_256) |  |  | (I) VT **Clozapine**  [^120^](#_ENREF_120)  (I)  Striatum  **Risperidone** [^93^](#_ENREF_93) | CB-839 | 24 |
| **PRPS1** Phosphoribosyl Pyrophosphate Synthetase 1 | 209440_at | (I) DE/4 57.3% |  |  | (I)Lymphoblastoid cells  **Lithium**  [^257^](#_ENREF_257) |  |  | 24 |
| **TMEM161B** Transmembrane Protein 161B | 227861_at | (I) AP/4 62.1% |  |  |  |  |  | 24 |
| **GLO1** glyoxalase I | 200681_at | (I) DE/2 41.5% | (I) Lymphocytes **Omega-3 fatty acids** [^256^](#_ENREF_256) |  |  |  |  | 22.5 |
| **FANCF** Fanconi Anemia Complementation Group F | 218689_at | (I) DE/4 54.9% |  |  |  |  |  | 22 |
| **HNRNPDL** heterogeneous nuclear ribonucleoprotein D like | 212454_x_at | (I) DE/2 35.4% | (I) NAC (females) **Omega-3 fatty acids** [^256^](#_ENREF_256) |  |  |  | (I) Lymphocytes **Diazepam** [^119^](#_ENREF_119) | 22 |
| **NRG1** neuregulin 1 Most Reproducible predictors | 208232_x_at | (D) AP/4 60.7% |  | (D) PBMC **Antidepressants** [^12^](#_ENREF_12)  **Antidepressants** [^251^](#_ENREF_251) | (NA)  SH-SY5Y neuroblastoma cells  **Lithium**  [^252^](#_ENREF_252)  (D) Neuroblastoma cells **Valproate** [^253^](#_ENREF_253) | (D)blood  **Antipsychotic with weight gain in men**  [^254^](#_ENREF_254) |  | 22 |
| **CD47** CD47 molecule | 213856_at | (I) AP/4 66.7% | (I) Lymphocytes (males) **Omega-3 fatty acids** [^256^](#_ENREF_256) | (I) Frontal Cortex **Venlafaxine** [^258^](#_ENREF_258) | (I)Corpus Collosum  **Lithium**[^259^](#_ENREF_259) | (I) Lymphocytes ,VT **Clozapine**  [^120^](#_ENREF_120) |  | 21 |
| **OLFM1** Olfactomedin 1 | 210924_at | (D) DE/2 33.7% |  |  | (D) NT2.D1 cells **Valproate** [^44^](#_ENREF_44) |  |  | 21 |
| **SMAD7** SMAD family member 7 | 204790_at | (I) DE/2 42.7% (I) AP/4 54.0% |  |  |  |  |  | 21 |
| **SLC6A4** solute carrier family 6 (neurotransmitter transporter), member 4 | 242009_at | (D) DE/4 64.1% | (D) Lymphocytes (females) **Omega-3 fatty acids** [^256^](#_ENREF_256) | (D) Neural progenitor cells (NPCs) **Imipramine, Citalopram** [^70^](#_ENREF_70) |  |  |  | 20 |

1. Hall, L.S. *et al.* Genome-wide meta-analyses of stratified depression in Generation Scotland and UK Biobank. *Transl Psychiatry* **8**, 9 (2018).

2. Yu, H. *et al.* A hypothesis-driven pathway analysis reveals myelin-related pathways that contribute to the risk of schizophrenia and bipolar disorder. *Prog Neuropsychopharmacol Biol Psychiatry* **51**, 140-5 (2014).

3. Walker, R.M. *et al.* Association analysis of Neuregulin 1 candidate regions in schizophrenia and bipolar disorder. *Neurosci Lett* **478**, 9-13 (2010).

4. Goes, F.S. *et al.* Family-based association study of Neuregulin 1 with psychotic bipolar disorder. *Am J Med Genet B Neuropsychiatr Genet* **150B**, 693-702 (2009).

5. Gutierrez-Fernandez, A. *et al.* Novel association of Neuregulin 1 gene with bipolar disorder but not with schizophrenia. *Schizophr Res* **159**, 552-3 (2014).

6. Thomson, P.A. *et al.* Association of Neuregulin 1 with schizophrenia and bipolar disorder in a second cohort from the Scottish population. *Mol Psychiatry* **12**, 94-104 (2007).

7. Biernacka, J.M. *et al.* Replication of genome wide association studies of alcohol dependence: support for association with variation in ADH1C. *PLoS One* **8**, e58798 (2013).

8. Benes, F.M., Lim, B. & Subburaju, S. Site-specific regulation of cell cycle and DNA repair in post-mitotic GABA cells in schizophrenic versus bipolars. *Proc Natl Acad Sci U S A* **106**, 11731-6 (2009).

9. Marballi, K., Cruz, D., Thompson, P. & Walss-Bass, C. Differential neuregulin 1 cleavage in the prefrontal cortex and hippocampus in schizophrenia and bipolar disorder: preliminary findings. *PLoS One* **7**, e36431 (2012).

10. Tochigi, M. *et al.* Gene expression profiling of major depression and suicide in the prefrontal cortex of postmortem brains. *Neurosci Res* **60**, 184-91 (2008).

11. Labonte, B. *et al.* Sex-specific transcriptional signatures in human depression. *Nat Med* **23**, 1102-1111 (2017).

12. Belzeaux, R. *et al.* Clinical variations modulate patterns of gene expression and define blood biomarkers in major depression. *J Psychiatr Res* **44**, 1205-13 (2010).

13. Begemann, M. *et al.* Episode-specific differential gene expression of peripheral blood mononuclear cells in rapid cycling supports novel treatment approaches. *Mol Med* **14**, 546-52 (2008).

14. Surget, A. *et al.* Corticolimbic transcriptome changes are state-dependent and region-specific in a rodent model of depression and of antidepressant reversal. *Neuropsychopharmacology* **34**, 1363-80 (2009).

15. Andrus, B.M. *et al.* Gene expression patterns in the hippocampus and amygdala of endogenous depression and chronic stress models. *Mol Psychiatry* **17**, 49-61 (2012).

16. Kataoka, M. *et al.* Exome sequencing for bipolar disorder points to roles of de novo loss-of-function and protein-altering mutations. *Mol Psychiatry* **21**, 885-93 (2016).

17. Beech, R.D. *et al.* Increased peripheral blood expression of electron transport chain genes in bipolar depression. *Bipolar Disord* **12**, 813-24 (2010).

18. Bagot, R.C. *et al.* Circuit-wide Transcriptional Profiling Reveals Brain Region-Specific Gene Networks Regulating Depression Susceptibility. *Neuron* **90**, 969-83 (2016).

19. Sequeira, A. *et al.* Global brain gene expression analysis links glutamatergic and GABAergic alterations to suicide and major depression. *PLoS One* **4**, e6585 (2009).

20. Chen, H. *et al.* Gene expression alterations in bipolar disorder postmortem brains. *Bipolar Disord* **15**, 177-87 (2013).

21. Zhurov, V. *et al.* Molecular pathway reconstruction and analysis of disturbed gene expression in depressed individuals who died by suicide. *PLoS One* **7**, e47581 (2012).

22. Gottschalk, M.G., Wesseling, H., Guest, P.C. & Bahn, S. Proteomic enrichment analysis of psychotic and affective disorders reveals common signatures in presynaptic glutamatergic signaling and energy metabolism. *Int J Neuropsychopharmacol* **18**(2014).

23. Martin, M.V. *et al.* Exon expression in lymphoblastoid cell lines from subjects with schizophrenia before and after glucose deprivation. *BMC Med Genomics* **2**, 62 (2009).

24. Le-Niculescu, H. *et al.* Phenomic, convergent functional genomic, and biomarker studies in a stress-reactive genetic animal model of bipolar disorder and co-morbid alcoholism. *Am J Med Genet B Neuropsychiatr Genet* **147B**, 134-66 (2008).

25. Forero, D.A., Guio-Vega, G.P. & Gonzalez-Giraldo, Y. A comprehensive regional analysis of genome-wide expression profiles for major depressive disorder. *J Affect Disord* **218**, 86-92 (2017).

26. Malki, K. *et al.* Identification of genes and gene pathways associated with major depressive disorder by integrative brain analysis of rat and human prefrontal cortex transcriptomes. *Transl Psychiatry* **5**, e519 (2015).

27. Kang, H.J. *et al.* Gene expression profiling in postmortem prefrontal cortex of major depressive disorder. *J Neurosci* **27**, 13329-40 (2007).

28. Dempster, E.L. *et al.* Disease-associated epigenetic changes in monozygotic twins discordant for schizophrenia and bipolar disorder. *Hum Mol Genet* **20**, 4786-96 (2011).

29. Howard, D.M. *et al.* Genome-wide meta-analysis of depression identifies 102 independent variants and highlights the importance of the prefrontal brain regions. *Nat Neurosci* **22**, 343-352 (2019).

30. Hyde, C.L. *et al.* Identification of 15 genetic loci associated with risk of major depression in individuals of European descent. *Nat Genet* **48**, 1031-6 (2016).

31. Kim, K.H. *et al.* Transcriptomic Analysis of Induced Pluripotent Stem Cells Derived from Patients with Bipolar Disorder from an Old Order Amish Pedigree. *PLoS One* **10**, e0142693 (2015).

32. Benes, F.M., Matzilevich, D., Burke, R.E. & Walsh, J. The expression of proapoptosis genes is increased in bipolar disorder, but not in schizophrenia. *Mol Psychiatry* **11**, 241-51 (2006).

33. Sun, L. [Clinical application of artificial synthetic hydroxyapatite in the obturation of cavities of the jaw]. *Zhonghua Kou Qiang Yi Xue Za Zhi* **24**, 340-2 (1989).

34. McMurray, K.M.J. *et al.* Identification of a novel, fast-acting GABAergic antidepressant. *Mol Psychiatry* **23**, 384-391 (2018).

35. Benton, C.S. *et al.* Evaluating genetic markers and neurobiochemical analytes for fluoxetine response using a panel of mouse inbred strains. *Psychopharmacology (Berl)* **221**, 297-315 (2012).

36. Gaiteri, C., Guilloux, J.P., Lewis, D.A. & Sibille, E. Altered gene synchrony suggests a combined hormone-mediated dysregulated state in major depression. *PLoS One* **5**, e9970 (2010).

37. Garbett, K.A. *et al.* Fibroblasts from patients with major depressive disorder show distinct transcriptional response to metabolic stressors. *Transl Psychiatry* **5**, e523 (2015).

38. McQuillin, A., Rizig, M. & Gurling, H.M. A microarray gene expression study of the molecular pharmacology of lithium carbonate on mouse brain mRNA to understand the neurobiology of mood stabilization and treatment of bipolar affective disorder. *Pharmacogenet Genomics* **17**, 605-17 (2007).

39. Takami, Y. *et al.* The activity of RhoA is correlated with lymph node metastasis in human colorectal cancer. *Dig Dis Sci* **53**, 467-73 (2008).

40. Jansen, R. *et al.* Gene expression in major depressive disorder. *Mol Psychiatry* **21**, 339-47 (2016).

41. Zubenko, G.S., Hughes, H.B., 3rd, Jordan, R.M., Lyons-Weiler, J. & Cohen, B.M. Differential hippocampal gene expression and pathway analysis in an etiology-based mouse model of major depressive disorder. *Am J Med Genet B Neuropsychiatr Genet* **165B**, 457-66 (2014).

42. Johnson, C., Drgon, T., McMahon, F.J. & Uhl, G.R. Convergent genome wide association results for bipolar disorder and substance dependence. *Am J Med Genet B Neuropsychiatr Genet* **150B**, 182-90 (2009).

43. Gandal, M.J. *et al.* Shared molecular neuropathology across major psychiatric disorders parallels polygenic overlap. *Science* **359**, 693-697 (2018).

44. Hill, E.J. *et al.* Effects of lithium and valproic acid on gene expression and phenotypic markers in an NT2 neurosphere model of neural development. *PLoS One* **8**, e58822 (2013).

45. Pajer, K. *et al.* Discovery of blood transcriptomic markers for depression in animal models and pilot validation in subjects with early-onset major depression. *Transl Psychiatry* **2**, e101 (2012).

46. Nakatani, N. *et al.* Genome-wide expression analysis detects eight genes with robust alterations specific to bipolar I disorder: relevance to neuronal network perturbation. *Hum Mol Genet* **15**, 1949-62 (2006).

47. Mamdani, F., Berlim, M.T., Beaulieu, M.M. & Turecki, G. Pharmacogenomic predictors of citalopram treatment outcome in major depressive disorder. *World J Biol Psychiatry* **15**, 135-44 (2014).

48. Hennings, J.M. *et al.* RNA expression profiling in depressed patients suggests retinoid-related orphan receptor alpha as a biomarker for antidepressant response. *Transl Psychiatry* **5**, e538 (2015).

49. Collier, D.A. *et al.* A novel functional polymorphism within the promoter of the serotonin transporter gene: possible role in susceptibility to affective disorders. *Mol Psychiatry* **1**, 453-60 (1996).

50. Lasky-Su, J.A., Faraone, S.V., Glatt, S.J. & Tsuang, M.T. Meta-analysis of the association between two polymorphisms in the serotonin transporter gene and affective disorders. *Am J Med Genet B Neuropsychiatr Genet* **133B**, 110-5 (2005).

51. Rotondo, A. *et al.* Catechol o-methyltransferase, serotonin transporter, and tryptophan hydroxylase gene polymorphisms in bipolar disorder patients with and without comorbid panic disorder. *Am J Psychiatry* **159**, 23-9 (2002).

52. Neves, F.S. *et al.* Is the 5-HTTLPR polymorphism associated with bipolar disorder or with suicidal behavior of bipolar disorder patients? *Am J Med Genet B Neuropsychiatr Genet* **147B**, 114-6 (2008).

53. Wang, T.Y. *et al.* Gender-specific association of the SLC6A4 and DRD2 gene variants in bipolar disorder. *Int J Neuropsychopharmacol* **17**, 211-22 (2014).

54. Neves, F.S. *et al.* Is the serotonin transporter polymorphism (5-HTTLPR) a potential marker for suicidal behavior in bipolar disorder patients? *J Affect Disord* **125**, 98-102 (2010).

55. Furlong, R.A. *et al.* Analysis and meta-analysis of two serotonin transporter gene polymorphisms in bipolar and unipolar affective disorders. *Am J Med Genet* **81**, 58-63 (1998).

56. Luykx, J.J. *et al.* Seasonal variation of serotonin turnover in human cerebrospinal fluid, depressive symptoms and the role of the 5-HTTLPR. *Transl Psychiatry* **3**, e311 (2013).

57. Ho, P.S. *et al.* Association study of serotonin transporter availability and SLC6A4 gene polymorphisms in patients with major depression. *Psychiatry Res* **212**, 216-22 (2013).

58. Lopez-Leon, S. *et al.* Meta-analyses of genetic studies on major depressive disorder. *Mol Psychiatry* **13**, 772-85 (2008).

59. Caspi, A. *et al.* Influence of life stress on depression: moderation by a polymorphism in the 5-HTT gene. *Science* **301**, 386-9 (2003).

60. Lopez de Lara, C. *et al.* STin2 variant and family history of suicide as significant predictors of suicide completion in major depression. *Biol Psychiatry* **59**, 114-20 (2006).

61. Kilpatrick, D.G. *et al.* The serotonin transporter genotype and social support and moderation of posttraumatic stress disorder and depression in hurricane-exposed adults. *Am J Psychiatry* **164**, 1693-9 (2007).

62. Muglia, P. *et al.* Genome-wide association study of recurrent major depressive disorder in two European case-control cohorts. *Mol Psychiatry* **15**, 589-601 (2010).

63. Lewis, C.M. *et al.* Genome-wide association study of major recurrent depression in the U.K. population. *Am J Psychiatry* **167**, 949-57 (2010).

64. Brezo, J. *et al.* Differences and similarities in the serotonergic diathesis for suicide attempts and mood disorders: a 22-year longitudinal gene-environment study. *Mol Psychiatry* **15**, 831-43 (2010).

65. Rybakowski, J.K. *et al.* Clinical and pathogenic aspects of candidate genes for lithium prophylactic efficacy. *J Psychopharmacol* **26**, 368-73 (2012).

66. Kato, T. & Iwamoto, K. Comprehensive DNA methylation and hydroxymethylation analysis in the human brain and its implication in mental disorders. *Neuropharmacology* **80**, 133-9 (2014).

67. Mann, J.J. *et al.* A serotonin transporter gene promoter polymorphism (5-HTTLPR) and prefrontal cortical binding in major depression and suicide. *Arch Gen Psychiatry* **57**, 729-38 (2000).

68. Hsu, J.W. *et al.* Association of thalamic serotonin transporter and interleukin-10 in bipolar I disorder: a SPECT study. *Bipolar Disord* **16**, 241-8 (2014).

69. Watanabe, S.Y. *et al.* Biological tests for major depressive disorder that involve leukocyte gene expression assays. *J Psychiatr Res* **66-67**, 1-6 (2015).

70. Lopez, J.P. *et al.* miR-1202 is a primate-specific and brain-enriched microRNA involved in major depression and antidepressant treatment. *Nat Med* **20**, 764-8 (2014).

71. Ponder, K.L. *et al.* Maternal depression and anxiety are associated with altered gene expression in the human placenta without modification by antidepressant use: implications for fetal programming. *Dev Psychobiol* **53**, 711-23 (2011).

72. Belzeaux, R., Azorin, J.M. & Ibrahim, E.C. Monitoring candidate gene expression variations before, during and after a first major depressive episode in a 51-year-old man. *BMC Psychiatry* **14**, 73 (2014).

73. Park, H. *et al.* Acupuncture stimulation at HT7 alleviates depression-induced behavioral changes via regulation of the serotonin system in the prefrontal cortex of maternally-separated rat pups. *J Physiol Sci* **62**, 351-7 (2012).

74. Hoyo-Becerra, C. *et al.* Concomitant interferon alpha stimulation and TLR3 activation induces neuronal expression of depression-related genes that are elevated in the brain of suicidal persons. *PLoS One* **8**, e83149 (2013).

75. Cordova-Palomera, A. *et al.* Genome-wide methylation study on depression: differential methylation and variable methylation in monozygotic twins. *Transl Psychiatry* **5**, e557 (2015).

76. Erikson, G.A. *et al.* Whole-Genome Sequencing of a Healthy Aging Cohort. *Cell* **165**, 1002-11 (2016).

77. Peters, M.J. *et al.* The transcriptional landscape of age in human peripheral blood. *Nat Commun* **6**, 8570 (2015).

78. Liu, J. *et al.* Patterns of gene expression in the frontal cortex discriminate alcoholic from nonalcoholic individuals. *Neuropsychopharmacology* **31**, 1574-82 (2006).

79. Patel, H., Dobson, R.J.B. & Newhouse, S.J. A Meta-Analysis of Alzheimer's Disease Brain Transcriptomic Data. *J Alzheimers Dis* **68**, 1635-1656 (2019).

80. Girgenti, M.J. *et al.* Transcriptomic Organization of Human Posttraumatic Stress Disorder. *bioRxiv*, 2020.01.27.921403 (2020).

81. Bowden, N.A. *et al.* Preliminary investigation of gene expression profiles in peripheral blood lymphocytes in schizophrenia. *Schizophr Res* **82**, 175-83 (2006).

82. Levey, D.F. *et al.* Towards understanding and predicting suicidality in women: biomarkers and clinical risk assessment. *Mol Psychiatry* **21**, 768-85 (2016).

83. Niculescu, A.B. *et al.* Precision medicine for suicidality: from universality to subtypes and personalization. *Mol Psychiatry* **22**, 1250-1273 (2017).

84. Le-Niculescu, H. *et al.* Towards precision medicine for stress disorders: diagnostic biomarkers and targeted drugs. *Mol Psychiatry* (2019).

85. Bagot, R.C. *et al.* Ketamine and Imipramine Reverse Transcriptional Signatures of Susceptibility and Induce Resilience-Specific Gene Expression Profiles. *Biol Psychiatry* **81**, 285-295 (2017).

86. Harris, S.E. *et al.* Age-related gene expression changes, and transcriptome wide association study of physical and cognitive aging traits, in the Lothian Birth Cohort 1936. *Aging (Albany NY)* **9**, 2489-2503 (2017).

87. Gulsuner, S. *et al.* Spatial and temporal mapping of de novo mutations in schizophrenia to a fetal prefrontal cortical network. *Cell* **154**, 518-29 (2013).

88. Coon, H. *et al.* Genome-wide significant regions in 43 Utah high-risk families implicate multiple genes involved in risk for completed suicide. *Mol Psychiatry* (2018).

89. Castillo, E. *et al.* Comparative profiling of cortical gene expression in Alzheimer's disease patients and mouse models demonstrates a link between amyloidosis and neuroinflammation. *Sci Rep* **7**, 17762 (2017).

90. Blalock, E.M. *et al.* Incipient Alzheimer's disease: microarray correlation analyses reveal major transcriptional and tumor suppressor responses. *Proc Natl Acad Sci U S A* **101**, 2173-8 (2004).

91. Chang, X. *et al.* RNA-seq analysis of amygdala tissue reveals characteristic expression profiles in schizophrenia. *Transl Psychiatry* **7**, e1203 (2017).

92. Hagihara, H., Ohira, K., Takao, K. & Miyakawa, T. Transcriptomic evidence for immaturity of the prefrontal cortex in patients with schizophrenia. *Mol Brain* **7**, 41 (2014).

93. Lanz, T.A. *et al.* Postmortem transcriptional profiling reveals widespread increase in inflammation in schizophrenia: a comparison of prefrontal cortex, striatum, and hippocampus among matched tetrads of controls with subjects diagnosed with schizophrenia, bipolar or major depressive disorder. *Transl Psychiatry* **9**, 151 (2019).

94. Niculescu, A.B. *et al.* Towards precision medicine for pain: diagnostic biomarkers and repurposed drugs. *Mol Psychiatry* **24**, 501-522 (2019).

95. Segman, R.H. *et al.* Peripheral blood mononuclear cell gene expression profiles identify emergent post-traumatic stress disorder among trauma survivors. *Mol Psychiatry* **10**, 500-13, 425 (2005).

96. Niculescu, A.B. *et al.* Blood biomarkers for memory: toward early detection of risk for Alzheimer disease, pharmacogenomics, and repurposed drugs. *Mol Psychiatry* (2019).

97. Rodd, Z.A. *et al.* Candidate genes, pathways and mechanisms for alcoholism: an expanded convergent functional genomics approach. *Pharmacogenomics J* **7**, 222-56 (2007).

98. Mehta, N.S., Wang, L. & Redei, E.E. Sex differences in depressive, anxious behaviors and hippocampal transcript levels in a genetic rat model. *Genes Brain Behav* **12**, 695-704 (2013).

99. Muhie, S. *et al.* Brain transcriptome profiles in mouse model simulating features of post-traumatic stress disorder. *Mol Brain* **8**, 14 (2015).

100. Roussos, P., Guennewig, B., Kaczorowski, D.C., Barry, G. & Brennand, K.J. Activity-Dependent Changes in Gene Expression in Schizophrenia Human-Induced Pluripotent Stem Cell Neurons. *JAMA Psychiatry* **73**, 1180-1188 (2016).

101. Parikshak, N.N. *et al.* Genome-wide changes in lncRNA, splicing, and regional gene expression patterns in autism. *Nature* **540**, 423-427 (2016).

102. Niculescu, A.B. *et al.* Understanding and predicting suicidality using a combined genomic and clinical risk assessment approach. *Mol Psychiatry* **20**, 1266-85 (2015).

103. Muench, C. *et al.* The major depressive disorder GWAS-supported variant rs10514299 in TMEM161B-MEF2C predicts putamen activation during reward processing in alcohol dependence. *Transl Psychiatry* **8**, 131 (2018).

104. Jansen, P.R. *et al.* Genome-wide analysis of insomnia in 1,331,010 individuals identifies new risk loci and functional pathways. *Nat Genet* **51**, 394-403 (2019).

105. Nowakowska, B.A. *et al.* Severe mental retardation, seizures, and hypotonia due to deletions of MEF2C. *Am J Med Genet B Neuropsychiatr Genet* **153B**, 1042-51 (2010).

106. van Heerden, J.H. *et al.* Parallel changes in gene expression in peripheral blood mononuclear cells and the brain after maternal separation in the mouse. *BMC Res Notes* **2**, 195 (2009).

107. Donner, J. *et al.* An association analysis of murine anxiety genes in humans implicates novel candidate genes for anxiety disorders. *Biol Psychiatry* **64**, 672-80 (2008).

108. Politi, P., Minoretti, P., Falcone, C., Martinelli, V. & Emanuele, E. Association analysis of the functional Ala111Glu polymorphism of the glyoxalase I gene in panic disorder. *Neurosci Lett* **396**, 163-6 (2006).

109. Junaid, M.A. *et al.* Proteomic studies identified a single nucleotide polymorphism in glyoxalase I as autism susceptibility factor. *Am J Med Genet A* **131**, 11-7 (2004).

110. Bowen, E.F.W., Burgess, J.L., Granger, R., Kleinman, J.E. & Rhodes, C.H. DLPFC transcriptome defines two molecular subtypes of schizophrenia. *Transl Psychiatry* **9**, 147 (2019).

111. Zahid, S., Oellerich, M., Asif, A.R. & Ahmed, N. Differential expression of proteins in brain regions of Alzheimer's disease patients. *Neurochem Res* **39**, 208-15 (2014).

112. Sanders, A.R. *et al.* Transcriptome study of differential expression in schizophrenia. *Hum Mol Genet* **22**, 5001-14 (2013).

113. Lehallier, B. *et al.* Undulating changes in human plasma proteome profiles across the lifespan. *Nat Med* **25**, 1843-1850 (2019).

114. Kromer, S.A. *et al.* Identification of glyoxalase-I as a protein marker in a mouse model of extremes in trait anxiety. *J Neurosci* **25**, 4375-84 (2005).

115. Hovatta, I. *et al.* Glyoxalase 1 and glutathione reductase 1 regulate anxiety in mice. *Nature* **438**, 662-6 (2005).

116. Tang, M. *et al.* Hippocampal proteomic changes of susceptibility and resilience to depression or anxiety in a rat model of chronic mild stress. *Transl Psychiatry* **9**, 260 (2019).

117. Kekesi, K.A. *et al.* Altered functional protein networks in the prefrontal cortex and amygdala of victims of suicide. *PLoS One* **7**, e50532 (2012).

118. Breen, M.S. *et al.* PTSD Blood Transcriptome Mega-Analysis: Shared Inflammatory Pathways across Biological Sex and Modes of Trauma. *Neuropsychopharmacology* **43**, 469-481 (2018).

119. Le-Niculescu, H. *et al.* Convergent functional genomics of anxiety disorders: translational identification of genes, biomarkers, pathways and mechanisms. *Transl Psychiatry* **1**, e9 (2011).

120. Le-Niculescu, H. *et al.* Towards understanding the schizophrenia code: an expanded convergent functional genomics approach. *Am J Med Genet B Neuropsychiatr Genet* **144B**, 129-58 (2007).

121. Malki, K. *et al.* Comparative mRNA analysis of behavioral and genetic mouse models of aggression. *Am J Med Genet B Neuropsychiatr Genet* **171B**, 427-36 (2016).

122. Sandercock, D.A. *et al.* Transcriptomics Analysis of Porcine Caudal Dorsal Root Ganglia in Tail Amputated Pigs Shows Long-Term Effects on Many Pain-Associated Genes. *Front Vet Sci* **6**, 314 (2019).

123. Le-Niculescu, H. *et al.* Identifying blood biomarkers for mood disorders using convergent functional genomics. *Mol Psychiatry* **14**, 156-74 (2009).

124. Uhl, G.R. *et al.* Genome-wide association for methamphetamine dependence: convergent results from 2 samples. *Arch Gen Psychiatry* **65**, 345-55 (2008).

125. Levine, M.E. & Crimmins, E.M. A Genetic Network Associated With Stress Resistance, Longevity, and Cancer in Humans. *J Gerontol A Biol Sci Med Sci* **71**, 703-12 (2016).

126. Dina, C. *et al.* Fine mapping of a region on chromosome 8p gives evidence for a QTL contributing to individual differences in an anxiety-related personality trait: TPQ harm avoidance. *Am J Med Genet B Neuropsychiatr Genet* **132B**, 104-8 (2005).

127. Bousman, C.A. *et al.* Effects of NRG1 and DAOA genetic variation on transition to psychosis in individuals at ultra-high risk for psychosis. *Transl Psychiatry* **3**, e251 (2013).

128. Mostaid, M.S. *et al.* Elevated peripheral expression of neuregulin-1 (NRG1) mRNA isoforms in clozapine-treated schizophrenia patients. *Transl Psychiatry* **7**, 1280 (2017).

129. Stefansson, H. *et al.* Association of neuregulin 1 with schizophrenia confirmed in a Scottish population. *Am J Hum Genet* **72**, 83-7 (2003).

130. Zhao, X. *et al.* A case control and family based association study of the neuregulin1 gene and schizophrenia. *J Med Genet* **41**, 31-4 (2004).

131. Rethelyi, J.M. *et al.* Association study of NRG1, DTNBP1, RGS4, G72/G30, and PIP5K2A with schizophrenia and symptom severity in a Hungarian sample. *Am J Med Genet B Neuropsychiatr Genet* **153B**, 792-801 (2010).

132. Bakker, S.C. *et al.* Neuregulin 1: genetic support for schizophrenia subtypes. *Mol Psychiatry* **9**, 1061-3 (2004).

133. Yang, J.Z. *et al.* Association study of neuregulin 1 gene with schizophrenia. *Mol Psychiatry* **8**, 706-9 (2003).

134. So, H.C. *et al.* Identification of neuroglycan C and interacting partners as potential susceptibility genes for schizophrenia in a Southern Chinese population. *Am J Med Genet B Neuropsychiatr Genet* **153B**, 103-13 (2010).

135. Mata, I. *et al.* A neuregulin 1 variant is associated with increased lateral ventricle volume in patients with first-episode schizophrenia. *Biol Psychiatry* **65**, 535-40 (2009).

136. Shi, T.Y. *et al.* DNA polymerase zeta as a potential biomarker of chemoradiation resistance and poor prognosis for cervical cancer. *Med Oncol* **30**, 500 (2013).

137. Williams, N.M. *et al.* Support for genetic variation in neuregulin 1 and susceptibility to schizophrenia. *Mol Psychiatry* **8**, 485-7 (2003).

138. Greenwood, T.A. *et al.* Analysis of 94 candidate genes and 12 endophenotypes for schizophrenia from the Consortium on the Genetics of Schizophrenia. *Am J Psychiatry* **168**, 930-46 (2011).

139. Papiol, S. *et al.* A phenotype-based genetic association study reveals the contribution of neuregulin1 gene variants to age of onset and positive symptom severity in schizophrenia. *Am J Med Genet B Neuropsychiatr Genet* **156B**, 340-5 (2011).

140. van Schijndel, J.E. *et al.* Three-cohort targeted gene screening reveals a non-synonymous TRKA polymorphism associated with schizophrenia. *J Psychiatr Res* **43**, 1195-9 (2009).

141. Petryshen, T.L. *et al.* Support for involvement of neuregulin 1 in schizophrenia pathophysiology. *Mol Psychiatry* **10**, 366-74, 328 (2005).

142. Hong, L.E., Wonodi, I., Stine, O.C., Mitchell, B.D. & Thaker, G.K. Evidence of missense mutations on the neuregulin 1 gene affecting function of prepulse inhibition. *Biol Psychiatry* **63**, 17-23 (2008).

143. Hatzimanolis, A. *et al.* Multiple variants aggregate in the neuregulin signaling pathway in a subset of schizophrenia patients. *Transl Psychiatry* **3**, e264 (2013).

144. Lachman, H.M. *et al.* Analysis of polymorphisms in AT-rich domains of neuregulin 1 gene in schizophrenia. *Am J Med Genet B Neuropsychiatr Genet* **141B**, 102-9 (2006).

145. Georgieva, L. *et al.* Support for neuregulin 1 as a susceptibility gene for bipolar disorder and schizophrenia. *Biol Psychiatry* **64**, 419-27 (2008).

146. Hanninen, K. *et al.* Interleukin-1 beta gene polymorphism and its interactions with neuregulin-1 gene polymorphism are associated with schizophrenia. *Eur Arch Psychiatry Clin Neurosci* **258**, 10-5 (2008).

147. Sokolowski, M., Wasserman, J. & Wasserman, D. Polygenic associations of neurodevelopmental genes in suicide attempt. *Mol Psychiatry* **21**, 1381-90 (2016).

148. Hashimoto, R. *et al.* Expression analysis of neuregulin-1 in the dorsolateral prefrontal cortex in schizophrenia. *Mol Psychiatry* **9**, 299-307 (2004).

149. Sheng, G., Demers, M., Subburaju, S. & Benes, F.M. Differences in the circuitry-based association of copy numbers and gene expression between the hippocampi of patients with schizophrenia and the hippocampi of patients with bipolar disorder. *Arch Gen Psychiatry* **69**, 550-61 (2012).

150. Hahn, C.G. *et al.* Altered neuregulin 1-erbB4 signaling contributes to NMDA receptor hypofunction in schizophrenia. *Nat Med* **12**, 824-8 (2006).

151. Chong, V.Z. *et al.* Elevated neuregulin-1 and ErbB4 protein in the prefrontal cortex of schizophrenic patients. *Schizophr Res* **100**, 270-80 (2008).

152. Tkachev, D. *et al.* Oligodendrocyte dysfunction in schizophrenia and bipolar disorder. *Lancet* **362**, 798-805 (2003).

153. Law, A.J. *et al.* Neuregulin 1-ErbB4-PI3K signaling in schizophrenia and phosphoinositide 3-kinase-p110delta inhibition as a potential therapeutic strategy. *Proc Natl Acad Sci U S A* **109**, 12165-70 (2012).

154. Kurian, S.M. *et al.* Identification of blood biomarkers for psychosis using convergent functional genomics. *Mol Psychiatry* **16**, 37-58 (2011).

155. Fernandez-Castillo, N. *et al.* Transcriptomic and genetic studies identify NFAT5 as a candidate gene for cocaine dependence. *Transl Psychiatry* **5**, e667 (2015).

156. Miller, G.E. *et al.* A functional genomic fingerprint of chronic stress in humans: blunted glucocorticoid and increased NF-kappaB signaling. *Biol Psychiatry* **64**, 266-72 (2008).

157. Vawter, M.P., Philibert, R., Rollins, B., Ruppel, P.L. & Osborn, T.W. Exon Array Biomarkers for the Differential Diagnosis of Schizophrenia and Bipolar Disorder. *Mol Neuropsychiatry* **3**, 197-213 (2018).

158. Brennand, K.J. *et al.* Modelling schizophrenia using human induced pluripotent stem cells. *Nature* **473**, 221-5 (2011).

159. Middleton, F.A. *et al.* Gene expression analysis of peripheral blood leukocytes from discordant sib-pairs with schizophrenia and bipolar disorder reveals points of convergence between genetic and functional genomic approaches. *Am J Med Genet B Neuropsychiatr Genet* **136B**, 12-25 (2005).

160. Chen, Y.J. *et al.* Type III neuregulin-1 is required for normal sensorimotor gating, memory-related behaviors, and corticostriatal circuit components. *J Neurosci* **28**, 6872-83 (2008).

161. Stefansson, H. *et al.* Neuregulin 1 and susceptibility to schizophrenia. *Am J Hum Genet* **71**, 877-92 (2002).

162. Papaleo, F. *et al.* Behavioral, Neurophysiological, and Synaptic Impairment in a Transgenic Neuregulin1 (NRG1-IV) Murine Schizophrenia Model. *J Neurosci* **36**, 4859-75 (2016).

163. O'Tuathaigh, C.M. *et al.* Phenotypic characterization of spatial cognition and social behavior in mice with 'knockout' of the schizophrenia risk gene neuregulin 1. *Neuroscience* **147**, 18-27 (2007).

164. Long, L.E. *et al.* Distinct neurobehavioural effects of cannabidiol in transmembrane domain neuregulin 1 mutant mice. *PLoS One* **7**, e34129 (2012).

165. Chen, Y.H. *et al.* ErbB4 signaling in the prelimbic cortex regulates fear expression. *Transl Psychiatry* **7**, e1168 (2017).

166. Olaya, J.C., Heusner, C.L., Matsumoto, M., Shannon Weickert, C. & Karl, T. Schizophrenia-relevant behaviours of female mice overexpressing neuregulin 1 type III. *Behav Brain Res* **353**, 227-235 (2018).

167. Zhang, L. *et al.* Mitochondria-focused gene expression profile reveals common pathways and CPT1B dysregulation in both rodent stress model and human subjects with PTSD. *Transl Psychiatry* **5**, e580 (2015).

168. Elfving, B. *et al.* Differential expression of synaptic markers regulated during neurodevelopment in a rat model of schizophrenia-like behavior. *Prog Neuropsychopharmacol Biol Psychiatry* **95**, 109669 (2019).

169. Patel, H. *et al.* Transcriptomic analysis of probable asymptomatic and symptomatic alzheimer brains. *Brain Behav Immun* **80**, 644-656 (2019).

170. Jin, E.H. *et al.* Genome-wide expression profiling of complex regional pain syndrome. *PLoS One* **8**, e79435 (2013).

171. Cameron, D., Blake, D.J., Bray, N.J. & Hill, M.J. Transcriptional Changes following Cellular Knockdown of the Schizophrenia Risk Gene SETD1A Are Enriched for Common Variant Association with the Disorder. *Mol Neuropsychiatry* **5**, 109-114 (2019).

172. Kuzman, M.R., Medved, V., Terzic, J. & Krainc, D. Genome-wide expression analysis of peripheral blood identifies candidate biomarkers for schizophrenia. *J Psychiatr Res* **43**, 1073-7 (2009).

173. Thalmeier, A. *et al.* Gene expression profiling of post-mortem orbitofrontal cortex in violent suicide victims. *Int J Neuropsychopharmacol* **11**, 217-28 (2008).

174. Hakak, Y. *et al.* Genome-wide expression analysis reveals dysregulation of myelination-related genes in chronic schizophrenia. *Proc Natl Acad Sci U S A* **98**, 4746-51 (2001).

175. Lewohl, J.M. *et al.* Gene expression in human alcoholism: microarray analysis of frontal cortex. *Alcohol Clin Exp Res* **24**, 1873-82 (2000).

176. Mehta, D. *et al.* Childhood maltreatment is associated with distinct genomic and epigenetic profiles in posttraumatic stress disorder. *Proc Natl Acad Sci U S A* **110**, 8302-7 (2013).

177. Lo, C.L. *et al.* High Resolution Genomic Scans Reveal Genetic Architecture Controlling Alcohol Preference in Bidirectionally Selected Rat Model. *PLoS Genet* **12**, e1006178 (2016).

178. Ouchi, Y., Kubota, Y., Kuramasu, A., Watanabe, T. & Ito, C. Gene expression profiling in whole cerebral cortices of phencyclidine- or methamphetamine-treated rats. *Brain Res Mol Brain Res* **140**, 142-9 (2005).

179. Miller, B.H. *et al.* MicroRNA-132 dysregulation in schizophrenia has implications for both neurodevelopment and adult brain function. *Proc Natl Acad Sci U S A* **109**, 3125-30 (2012).

180. Mills, J.D. *et al.* RNA-Seq analysis of the parietal cortex in Alzheimer's disease reveals alternatively spliced isoforms related to lipid metabolism. *Neurosci Lett* **536**, 90-5 (2013).

181. Kim, K.S. & Han, P.L. Optimization of chronic stress paradigms using anxiety- and depression-like behavioral parameters. *J Neurosci Res* **83**, 497-507 (2006).

182. Feinn, R., Nellissery, M. & Kranzler, H.R. Meta-analysis of the association of a functional serotonin transporter promoter polymorphism with alcohol dependence. *Am J Med Genet B Neuropsychiatr Genet* **133B**, 79-84 (2005).

183. Guo, G., Wilhelmsen, K. & Hamilton, N. Gene-lifecourse interaction for alcohol consumption in adolescence and young adulthood: five monoamine genes. *Am J Med Genet B Neuropsychiatr Genet* **144B**, 417-23 (2007).

184. Seneviratne, C., Huang, W., Ait-Daoud, N., Li, M.D. & Johnson, B.A. Characterization of a functional polymorphism in the 3' UTR of SLC6A4 and its association with drinking intensity. *Alcohol Clin Exp Res* **33**, 332-9 (2009).

185. Bordukalo-Niksic, T., Stefulj, J., Matosic, A., Mokrovic, G. & Cicin-Sain, L. Combination of polymorphic variants in serotonin transporter and monoamine oxidase-A genes may influence the risk for early-onset alcoholism. *Psychiatry Res* **200**, 1041-3 (2012).

186. Druley, T.E. *et al.* Candidate gene resequencing to identify rare, pedigree-specific variants influencing healthy aging phenotypes in the long life family study. *BMC Geriatr* **16**, 80 (2016).

187. Li, P. *et al.* Promoter polymorphism in the serotonin transporter (5-HTT) gene is significantly associated with leukocyte telomere length in Han Chinese. *PLoS One* **9**, e94442 (2014).

188. Gondo, Y. *et al.* Contribution of an affect-associated gene to human longevity: prevalence of the long-allele genotype of the serotonin transporter-linked gene in Japanese centenarians. *Mech Ageing Dev* **126**, 1178-84 (2005).

189. Mizuno, T. *et al.* Gender difference in association between polymorphism of serotonin transporter gene regulatory region and anxiety. *J Psychosom Res* **60**, 91-7 (2006).

190. Costas, J. *et al.* Association study of 44 candidate genes with depressive and anxiety symptoms in post-partum women. *J Psychiatr Res* **44**, 717-24 (2010).

191. Forstner, A.J. *et al.* Further evidence for genetic variation at the serotonin transporter gene SLC6A4 contributing toward anxiety. *Psychiatr Genet* **27**, 96-102 (2017).

192. Wray, N.R. *et al.* Accurate, Large-Scale Genotyping of 5HTTLPR and Flanking Single Nucleotide Polymorphisms in an Association Study of Depression, Anxiety, and Personality Measures. *Biol Psychiatry* **66**, 468-76 (2009).

193. Denys, D., Van Nieuwerburgh, F., Deforce, D. & Westenberg, H.G. Association between serotonergic candidate genes and specific phenotypes of obsessive compulsive disorder. *J Affect Disord* **91**, 39-44 (2006).

194. Wendland, J.R. *et al.* A novel, putative gain-of-function haplotype at SLC6A4 associates with obsessive-compulsive disorder. *Hum Mol Genet* **17**, 717-23 (2008).

195. Ozaki, N. *et al.* Serotonin transporter missense mutation associated with a complex neuropsychiatric phenotype. *Mol Psychiatry* **8**, 933-6 (2003).

196. Lin, P.Y. Meta-analysis of the association of serotonin transporter gene polymorphism with obsessive-compulsive disorder. *Prog Neuropsychopharmacol Biol Psychiatry* **31**, 683-9 (2007).

197. Wendland, J.R., Kruse, M.R., Cromer, K.R. & Murphy, D.L. A large case-control study of common functional SLC6A4 and BDNF variants in obsessive-compulsive disorder. *Neuropsychopharmacology* **32**, 2543-51 (2007).

198. Voyiaziakis, E. *et al.* Association of SLC6A4 variants with obsessive-compulsive disorder in a large multicenter US family study. *Mol Psychiatry* **16**, 108-20 (2011).

199. Saiz, P.A. *et al.* Association study between obsessive-compulsive disorder and serotonergic candidate genes. *Prog Neuropsychopharmacol Biol Psychiatry* **32**, 765-70 (2008).

200. Kistner-Griffin, E. *et al.* Parent-of-origin effects of the serotonin transporter gene associated with autism. *Am J Med Genet B Neuropsychiatr Genet* **156**, 139-44 (2011).

201. Coutinho, A.M. *et al.* Evidence for epistasis between SLC6A4 and ITGB3 in autism etiology and in the determination of platelet serotonin levels. *Hum Genet* **121**, 243-56 (2007).

202. Ma, D.Q. *et al.* Association and gene-gene interaction of SLC6A4 and ITGB3 in autism. *Am J Med Genet B Neuropsychiatr Genet* **153B**, 477-483 (2010).

203. Zaboli, G. *et al.* Haplotype analysis confirms association of the serotonin transporter (5-HTT) gene with schizophrenia but not with major depression. *Am J Med Genet B Neuropsychiatr Genet* **147**, 301-7 (2008).

204. Hung, C.F. *et al.* Association between suicide attempt and a tri-allelic functional polymorphism in serotonin transporter gene promoter in Chinese patients with schizophrenia. *Neurosci Lett* **504**, 242-6 (2011).

205. Bayle, F.J. *et al.* 5HTTLPR polymorphism in schizophrenic patients: further support for association with violent suicide attempts. *Am J Med Genet B Neuropsychiatr Genet* **119B**, 13-7 (2003).

206. De Luca, V. *et al.* Association study between the novel functional polymorphism of the serotonin transporter gene and suicidal behaviour in schizophrenia. *Eur Neuropsychopharmacol* **16**, 268-71 (2006).

207. Lindholm Carlstrom, E. *et al.* Association between a genetic variant in the serotonin transporter gene (SLC6A4) and suicidal behavior in patients with schizophrenia. *Behav Brain Funct* **8**, 24 (2012).

208. Vassos, E., Collier, D.A. & Fazel, S. Systematic meta-analyses and field synopsis of genetic association studies of violence and aggression. *Mol Psychiatry* **19**, 471-7 (2014).

209. Perroud, N. *et al.* Rare genotype combination of the serotonin transporter gene associated with treatment response in severe personality disorder. *Am J Med Genet B Neuropsychiatr Genet* **153B**, 1494-7 (2010).

210. Sen, S., Burmeister, M. & Ghosh, D. Meta-analysis of the association between a serotonin transporter promoter polymorphism (5-HTTLPR) and anxiety-related personality traits. *Am J Med Genet B Neuropsychiatr Genet* **127B**, 85-9 (2004).

211. Offenbaecher, M. *et al.* Possible association of fibromyalgia with a polymorphism in the serotonin transporter gene regulatory region. *Arthritis Rheum* **42**, 2482-8 (1999).

212. Cui, W., Yu, X. & Zhang, H. The serotonin transporter gene polymorphism is associated with the susceptibility and the pain severity in idiopathic trigeminal neuralgia patients. *J Headache Pain* **15**, 42 (2014).

213. Tour, J. *et al.* Gene-to-gene interactions regulate endogenous pain modulation in fibromyalgia patients and healthy controls-antagonistic effects between opioid and serotonin-related genes. *Pain* **158**, 1194-1203 (2017).

214. James, S. Human pain and genetics: some basics. *Br J Pain* **7**, 171-8 (2013).

215. Zorina-Lichtenwalter, K., Meloto, C.B., Khoury, S. & Diatchenko, L. Genetic predictors of human chronic pain conditions. *Neuroscience* **338**, 36-62 (2016).

216. Treister, R. *et al.* Association between polymorphisms in serotonin and dopamine-related genes and endogenous pain modulation. *J Pain* **12**, 875-83 (2011).

217. Maron, E. *et al.* Associations between serotonin-related gene polymorphisms and panic disorder. *Int J Neuropsychopharmacol* **8**, 261-6 (2005).

218. Lonsdorf, T.B. *et al.* The symptomatic profile of panic disorder is shaped by the 5-HTTLPR polymorphism. *Prog Neuropsychopharmacol Biol Psychiatry* **33**, 1479-83 (2009).

219. Gyawali, S. *et al.* Association of a polyadenylation polymorphism in the serotonin transporter and panic disorder. *Biol Psychiatry* **67**, 331-8 (2010).

220. Strug, L.J. *et al.* Panic disorder is associated with the serotonin transporter gene (SLC6A4) but not the promoter region (5-HTTLPR). *Mol Psychiatry* **15**, 166-76 (2010).

221. Lee, H.J. *et al.* Influence of the serotonin transporter promoter gene polymorphism on susceptibility to posttraumatic stress disorder. *Depress Anxiety* **21**, 135-9 (2005).

222. Zhang, K. *et al.* An overview of posttraumatic stress disorder genetic studies by analyzing and integrating genetic data into genetic database PTSDgene. *Neurosci Biobehav Rev* **83**, 647-656 (2017).

223. Bondy, B., Erfurth, A., de Jonge, S., Kruger, M. & Meyer, H. Possible association of the short allele of the serotonin transporter promoter gene polymorphism (5-HTTLPR) with violent suicide. *Mol Psychiatry* **5**, 193-5 (2000).

224. Cicchetti, D., Rogosch, F.A., Sturge-Apple, M. & Toth, S.L. Interaction of child maltreatment and 5-HTT polymorphisms: suicidal ideation among children from low-SES backgrounds. *J Pediatr Psychol* **35**, 536-46 (2010).

225. Courtet, P. *et al.* Serotonin transporter gene may be involved in short-term risk of subsequent suicide attempts. *Biol Psychiatry* **55**, 46-51 (2004).

226. Gaysina, D., Zainullina, A., Gabdulhakov, R. & Khusnutdinova, E. The serotonin transporter gene: polymorphism and haplotype analysis in Russian suicide attempters. *Neuropsychobiology* **54**, 70-4 (2006).

227. Dell'osso, L. *et al.* Temperamental and genetic predictors of suicide attempt and self-mutilation. *Neuropsychobiology* **68**, 250-7 (2013).

228. Joiner, T.E., Jr., Johnson, F. & Soderstrom, K. Association between serotonin transporter gene polymorphism and family history of attempted and completed suicide. *Suicide Life Threat Behav* **32**, 329-32 (2002).

229. Li, D. & He, L. Meta-analysis supports association between serotonin transporter (5-HTT) and suicidal behavior. *Mol Psychiatry* **12**, 47-54 (2007).

230. Campi-Azevedo, A.C., Boson, W., De Marco, L., Romano-Silva, M.A. & Correa, H. Association of the serotonin transporter promoter polymorphism with suicidal behavior. *Mol Psychiatry* **8**, 899-900 (2003).

231. Wasserman, D. *et al.* Association of the serotonin transporter promotor polymorphism with suicide attempters with a high medical damage. *Eur Neuropsychopharmacol* **17**, 230-3 (2007).

232. Hranilovic, D. *et al.* Serotonin transporter gene promoter (5-HTTLPR) and intron 2 (VNTR) polymorphisms in Croatian suicide victims. *Biol Psychiatry* **54**, 884-9 (2003).

233. Akar, T. *et al.* Investigation of serotonin transporter gene promoter (5-HTTLPR) and intron 2 (variable number of tandem repeats) polymorphisms with suicidal behavior in a Turkish population. *DNA Cell Biol* **29**, 429-34 (2010).

234. Saiz, P.A. *et al.* Role of serotonergic-related systems in suicidal behavior: Data from a case-control association study. *Prog Neuropsychopharmacol Biol Psychiatry* **35**, 1518-24 (2011).

235. Anguelova, M., Benkelfat, C. & Turecki, G. A systematic review of association studies investigating genes coding for serotonin receptors and the serotonin transporter: II. Suicidal behavior. *Mol Psychiatry* **8**, 646-53 (2003).

236. Gibb, B.E., McGeary, J.E., Beevers, C.G. & Miller, I.W. Serotonin transporter (5-HTTLPR) genotype, childhood abuse, and suicide attempts in adult psychiatric inpatients. *Suicide Life Threat Behav* **36**, 687-93 (2006).

237. Roy, A., Hu, X.Z., Janal, M.N. & Goldman, D. Interaction between childhood trauma and serotonin transporter gene variation in suicide. *Neuropsychopharmacology* **32**, 2046-52 (2007).

238. Bah, J. *et al.* Serotonin transporter gene polymorphisms: effect on serotonin transporter availability in the brain of suicide attempters. *Psychiatry Res* **162**, 221-9 (2008).

239. Clayden, R.C., Zaruk, A., Meyre, D., Thabane, L. & Samaan, Z. The association of attempted suicide with genetic variants in the SLC6A4 and TPH genes depends on the definition of suicidal behavior: a systematic review and meta-analysis. *Transl Psychiatry* **2**, e166 (2012).

240. Jernej, B. *et al.* Intronic polymorphism of tryptophan hydroxylase and serotonin transporter: indication for combined effect in predisposition to suicide. *J Neural Transm (Vienna)* **111**, 733-8 (2004).

241. Schneider, E., El Hajj, N., Muller, F., Navarro, B. & Haaf, T. Epigenetic Dysregulation in the Prefrontal Cortex of Suicide Completers. *Cytogenet Genome Res* **146**, 19-27 (2015).

242. Gross-Isseroff, R., Israeli, M. & Biegon, A. Autoradiographic analysis of tritiated imipramine binding in the human brain post mortem: effects of suicide. *Arch Gen Psychiatry* **46**, 237-41 (1989).

243. Peng, H. *et al.* Childhood Trauma, DNA Methylation of Stress-Related Genes, and Depression: Findings From Two Monozygotic Twin Studies. *Psychosom Med* **80**, 599-608 (2018).

244. Azadmarzabadi, E., Haghighatfard, A. & Mohammadi, A. Low resilience to stress is associated with candidate gene expression alterations in the dopaminergic signalling pathway. *Psychogeriatrics* **18**, 190-201 (2018).

245. Hu, X.Z. *et al.* Serotonin transporter promoter gain-of-function genotypes are linked to obsessive-compulsive disorder. *Am J Hum Genet* **78**, 815-826 (2006).

246. Seneviratne, C. & Johnson, B.A. Serotonin transporter genomic biomarker for quantitative assessment of ondansetron treatment response in alcoholics. *Front Psychiatry* **3**, 23 (2012).

247. Samuelsson, M., Jokinen, J., Nordstrom, A.L. & Nordstrom, P. CSF 5-HIAA, suicide intent and hopelessness in the prediction of early suicide in male high-risk suicide attempters. *Acta Psychiatr Scand* **113**, 44-7 (2006).

248. Jones, K.L. *et al.* Combined effect of maternal serotonin transporter genotype and prenatal stress in modulating offspring social interaction in mice. *Int J Dev Neurosci* **28**, 529-36 (2010).

249. de Almeida Magalhaes, T., Correia, D., de Carvalho, L.M., Damasceno, S. & Brunialti Godard, A.L. Maternal separation affects expression of stress response genes and increases vulnerability to ethanol consumption. *Brain Behav* **8**, e00841 (2018).

250. Zhang, S., Amstein, T., Shen, J., Brush, F.R. & Gershenfeld, H.K. Molecular correlates of emotional learning using genetically selected rat lines. *Genes Brain Behav* **4**, 99-109 (2005).

251. Biernacka, J.M. *et al.* The International SSRI Pharmacogenomics Consortium (ISPC): a genome-wide association study of antidepressant treatment response. *Transl Psychiatry* **5**, e553 (2015).

252. Gow, M., Mirembe, D., Longwe, Z. & Pickard, B.S. A gene trap mutagenesis screen for genes underlying cellular response to the mood stabilizer lithium. *J Cell Mol Med* **17**, 657-63 (2013).

253. Warburton, A. *et al.* Molecular signatures of mood stabilisers highlight the role of the transcription factor REST/NRSF. *J Affect Disord* **172**, 63-73 (2015).

254. Sainz, J., Prieto, C. & Crespo-Facorro, B. Sex differences in gene expression related to antipsychotic induced weight gain. *PLoS One* **14**, e0215477 (2019).

255. Huttenrauch, M., Salinas, G. & Wirths, O. Effects of Long-Term Environmental Enrichment on Anxiety, Memory, Hippocampal Plasticity and Overall Brain Gene Expression in C57BL6 Mice. *Front Mol Neurosci* **9**, 62 (2016).

256. Le-Niculescu, H. *et al.* Convergent functional genomic studies of omega-3 fatty acids in stress reactivity, bipolar disorder and alcoholism. *Transl Psychiatry* **1**, e4 (2011).

257. Chen, H., Wang, N., Burmeister, M. & McInnis, M.G. MicroRNA expression changes in lymphoblastoid cell lines in response to lithium treatment. *Int J Neuropsychopharmacol* **12**, 975-81 (2009).

258. Tamasi, V. *et al.* Transcriptional evidence for the role of chronic venlafaxine treatment in neurotrophic signaling and neuroplasticity including also Glutamatergic [corrected] - and insulin-mediated neuronal processes. *PLoS One* **9**, e113662 (2014).

259. Akkouh, I.A. *et al.* Exploring lithium's transcriptional mechanisms of action in bipolar disorder: a multi-step study. *Neuropsychopharmacology* (2019).
